# How has Acceptability Been Defined and Measured in Cancer Screening Research? A Scoping Review

**DOI:** 10.1002/pon.70578

**Published:** 2026-08-20

**Authors:** Ninian Schmeising‐Barnes, Christina Derksen, Charlotte Kelley Jones, Pranay Ruparelia, Ruth E. C. Evans, Evangelos Katsampouris, Emma Lidington, Lorna McWilliams, Sarah Hindmarch, Anthony Tsang, Adriana B. Akbar, Asha V. E. Parmar, Suzanne E. Scott, Jo Waller, Lauren Gatting

**Affiliations:** ^1^ Wolfson Institute of Population Health Queen Mary University of London London UK; ^2^ Guy's, King's and St Thomas' School of Medical Education King's College London London UK; ^3^ Division of Psychology and Mental Health, School of Health Sciences, Faculty of Biology, Medicine and Health University of Manchester Manchester UK; ^4^ The London School of Medicine and Dentistry, Faculty of Medicine and Dentistry Queen Mary University of London London UK; ^5^ Health Innovation West of England Royal United Hospitals Bath UK

**Keywords:** acceptability, cancer, conceptualisation, definition, methodology, oncology, operationalisation, review, screening

## Abstract

**Objectives:**

Cancer screening is crucial for early detection and improved outcomes globally. Screening must be “acceptable” to health professionals and the public. However, acceptability definitions and measures have not always been made explicit. This scoping review aimed to explore the definitions, theories, and methods used by researchers investigating acceptability.

**Methods:**

We conducted a term‐based scoping review of published literature in six databases. Two independent reviewers screened citations for inclusion. Study characteristics (publication year, country, study design, sample size, population, methodological characteristics), characteristics of acceptability (definition, theory, test or device), and screening context (cancer type, screening procedure) were extracted by one author and checked independently by a second author. Results were reported descriptively. Definitions and operationalisations of acceptability were analysed through content analysis.

**Results:**

We identified 393 studies, largely published after 2013. Most examined patient or public attitudes towards self‐sampling for cervical screening. Few studies cited theory (*n* = 55, 14%) or provided a definition of acceptability (*n* = 17, 4.3%). Acceptability was typically defined as a multidimensional construct or as perceptions or attitudes towards screening. Studies operationalised acceptability most often as physical side‐effects (*n* = 68, 42.8%), willingness to attend screening (*n* = 59, 29.4%), screening method preference (*n* = 55, 27.4%), ease of use (*n* = 52, 25.9%), and general attitudes (*n* = 51, 25.4%).

**Conclusions:**

Research focussed on acceptability in cancer screening has not been based on theory and uses a range of operationalisations, including loosely related constructs such as uptake. A specific definition of acceptability in cancer screening and reporting guidelines for interdisciplinary research are needed to support evidence‐based decision‐making.

## 1 Background

Globally, cancer continues to be a leading cause of morbidity and mortality [1]. Cancer screening programmes are crucial for early detection and improved prognosis [2]. With technological and computational advances, the tests and processes that can be used for cancer screening programmes are becoming more clinically effective and accessible on a global scale [3], but their acceptability needs to be assessed in parallel.

The concept of acceptability has been growing in popularity as a key evaluation criterion of healthcare interventions, generally, and in the context of cancer screening, specifically. Major international healthcare and research bodies including the World Health Organization, International Agency for Research on Cancer, and the US National Cancer Institute, similarly recommend evaluating acceptability when developing cancer screening programmes [4, 5, 6]. The UK National Screening Committee (UKNSC) is the key advisory organisation making recommendations to government about the introduction, modification or discontinuation of screening programmes. One criterion upon which the UKNSC evaluates screening (including cancer screening) is whether “the complete screening programme (test, diagnostic procedures, treatment/intervention) is clinically, socially and ethically *acceptable* to health professionals and the public” [7]. The UK's Medical Research Council's (MRC) framework for designing and evaluating complex interventions includes acceptability as a key evaluation criterion [8]. The CanTest Framework for evaluating strategies for the early detection of cancer includes acceptability as an outcome to be included in any evaluation of cancer tests and diagnostic approaches [9].

However, such frameworks do not provide a definition of acceptability or guidance on specific factors that must be considered and measured when seeking to assess the acceptability of a cancer screening programme. If the concept of “acceptability” is not consistently defined for all stakeholders (e.g., healthcare professionals, patients, policy makers and third sector organisations), there is a risk that decision‐making about screening programmes is not evidence‐based and programmes therefore do not actually meet key evaluation criteria, despite a lot of research being conducted [10, 11].

Sekhon and colleagues (2017) proposed a general definition and theoretical framework for assessing acceptability of healthcare interventions, based on a review of systematic reviews followed by a consensus activity [12]. Despite this, cancer screening is arguably a fairly unique healthcare intervention. Unlike most healthcare interventions, it is typically offered to asymptomatic participants. In addition, cancer screening is unusual in that it occurs only sporadically, and that it has several components such as the invitation, booking, initial test, results communication, follow up procedures, and final diagnosis [13]. Populations and healthcare contexts differ greatly across screening approaches—with differing processes, pressures, and priorities [14]. For example, issues related to the acceptability of a new type of screening for people at higher risk of cancer might be very different to more general population‐based screening or changing screening intervals of existing programmes [15, 16, 17]. A generic definition of acceptability designed to apply across healthcare interventions may not be appropriate to capture the nuances cancer screening contexts present.

Additionally, different stakeholders have varying motivations for assessing acceptability. For example, screening programme evaluators may focus on uptake rate as a measure of public acceptability, since screening only delivers population‐level benefits with optimal uptake [18]. They may also need to ensure that tests are acceptable to healthcare providers to optimise delivery compliance [19, 20]. Alternatively, developers of a new screening test may prioritise how prospective users think and feel about a screening test, to uphold commitments to ensuring positive experience and wellbeing [21]. Therefore, conceptualisations of acceptability should be specific to stakeholder priorities and context.

A recent consensus survey identified key challenges of assessing acceptability in relation to cancer screening, including poor distinction between related constructs, disparities between hypothetical and real‐world acceptability, and lack of clarity about evaluation [22].

The aim of this scoping review was to assess how the concept “acceptability” has been defined and operationalised in research on cancer screening. We aimed to assess definitions and methods used by researchers when they *explicitly state* that they are investigating the acceptability of population‐based cancer screening tests, interventions, and processes, and how definitions might have changed over time.

Specific research questions were to identify:

1. What definitions are provided for acceptability?
2. What theories or models of acceptability have been used, and how has acceptability been operationalised?
3. What methodological approaches to assessing acceptability have researchers applied?

## 2 Methods

This scoping review is a term‐based review around the term “acceptability” in the context of cancer screening to: (1) understand how acceptability has been defined and measured in previous research, and, (2) explore what consequences this might potentially have had for evidence‐based decision‐making. It is reported in line with the PRISMA statement extension for scoping reviews (PRISMA‐ScR; [23]). The review protocol is available on OSF: https://osf.io/cqdr2/overview and was uploaded before the review was conducted. The PRISMA checklist can be found in the Supporting Information S1: Appendix A.

### 2.1 Data Sources and Search Strategy

Data sources were published primary research studies identified in database searches. We also examined reference lists from relevant evidence syntheses (including literature reviews, systematic reviews and meta‐analyses). All studies cited in eligible and accessible reviews were uploaded to Rayyan and their titles and abstracts were screened in line with inclusion criteria. A list of all reviews that references were pulled from is available in the Supporting Information S1: Appendix A. Searches were conducted on 14th August 2023 and updated on 26th February 2025 using PsycINFO (Ovid), MEDLINE (Ovid), Cochrane Central Register of Controlled Trials (CENTRAL), Science Citation Index Expanded (Web of Science), Social Sciences Citation Index (Web of Science), and CINAHL (EBSCO), limited to English papers. To create a balanced search, a combination of search techniques was employed including proximity searches and the use of subheadings/qualifiers in combination with medical subject headings (MeSH), including terms around “acceptability” (e.g., accept or acceptable) and “cancer screening” (e.g., neoplasm or tumour/tumour near screening/early detection).

All search strategies can be found in the Supporting Information S1: Appendix A.

### 2.2 Inclusion and Exclusion Criteria

We included primary research studies that explicitly used the term “acceptability” in the context of cancer screening. As the term acceptability would likely deviate in meaning when translated to other languages, we only included primary research studies published in the English language. We included papers regardless of study design, sample size, year of publication, and study country to include a wide range of conceptualisations and measures of acceptability across contexts and over time.

We used the PCC (Population/Concept/Context) framework [24] to inform our inclusion criteria regarding objectives and scope of papers (see Box 1).

**BOX 1 Inclusion criteria based on PCC scheme.**

1

**Table:** 

Population
Human populations across ages, genders, races and ethnicities since acceptability is an assessment criterion used across populations.
Concept
As the goal was to investigate how research was conducted when authors claimed to be investigating acceptability, studies were included in which the term “acceptability” was used at least once to describe primary or secondary outcomes. Variants such as acceptance, accepted, acceptable, accepting, accept, acceptors could be used in addition, but papers only using these terms and not “acceptability” were excluded.
Context
Any clinical test that is used as the initial test of a cancer screening pathway, either a new approach or an alteration to an existing approach, e.g., using a test, device, technology or tool as an initial test for cancer screening, a change to the intervals or eligibility criteria for cancer screening or changing screening procedures, e.g., restricting screening at end‐of‐life or combining two screening procedures.

Studies were not included if they used terms arguably related to acceptability (e.g., preferences, intentions, willingness, or uptake) but did not also use the term “acceptability” explicitly to describe what they assessed as these papers would not capture what was measured when the term “acceptability” was used. Studies were excluded if the related terms, for example, “accept” and “accepted” were present but “acceptability” absent on a similar basis. These terms were included in the search strategy to improve the sensitivity of the search to identify papers that state they are measuring “acceptability”, but we believe these terms to be sufficiently different from “acceptability” to consider them not interchangeable in this term‐based scoping review. Studies were also excluded if they focussed on communication interventions or behavioural interventions rather than clinical interventions, for example, education, decision aids or support, alternative communication methods, health literacy, or awareness raising campaigns. We also excluded studies that examined follow‐up tests after an initial screening test, for example, colposcopy following cervical screening or a biopsy following mammography screening. Interventions such as HPV vaccination which aim to prevent cancer were not included.

### 2.3 Procedure and Extraction

The search results identified in each database were exported and saved to EndNote Reference Manager for initial de‐duplication. Records were then exported to Rayyan Review software (https://www.rayyan.ai/) for the screening process. Two independent reviewers screened all titles and abstracts, with a third reviewer arbitrating any conflicts.

For full‐text screening, records were excluded if full texts could not be accessed through institutional access. All full texts were double screened and arbitrated by a third reviewer if necessary.

A Microsoft Forms data extraction sheet was developed by the research team to extract study characteristics (see Supporting Information S1: Appendix A). The extraction sheet was piloted by a subset of extractors on a proportion of randomly selected papers to assess suitability and usability. Extracted study characteristics included design elements (data collection method and analytic method), sample size(s), and population characteristics (age, race or ethnicity, and gender/sex). A range of characteristics of acceptability were extracted, including definition of acceptability and reference to acceptability theory or models. Source and methodological characteristics were extracted on a separate sheet such as author(s), title, year of publication, country of origin, type of cancer, and screening procedure, test or device and were combined into the final table including all extracted data.

To verify extraction reliability, two independent reviewers extracted data from 46 studies (12%). This highlighted 24%–26% disagreement on specific characteristics including: specific population, sample size used to assess acceptability, and specific methods (caused by inconsistencies in extracting attendance records vs. surveys for screening uptake). All data extraction was double‐checked by a third reviewer, with disagreements resolved through discussion (with the wider team if needed).

A table with all extracted data is provided in Supporting Information S2: Appendix B.

### 2.4 Data Analysis

We tabulated extracted data and provided frequencies of participant characteristics, acceptability characteristics, and methodology at an aggregated level and separately for different population groups (e.g., patients/the public and healthcare professionals). Trends over time were examined by grouping studies into decades (e.g., 2010–2019).

Research question 1: What definitions are provided for acceptability?

For definitions of acceptability, the frequency and proportion of studies that gave a definition was quantified including studies that created their own definition and those using a definition based on theory. We conducted an inductive content analysis of definitions and operationalisations of acceptability using NVivo 14. Two reviewers (CD and NSB) independently coded statements from all studies providing definitions and operationalisations. Differences were resolved by discussion with the study team.

Research question 2: What theories or models of acceptability have been used, and how has acceptability been operationalised?

The frequency and proportion of studies that used theory were quantified and grouped under different theories.

Research question 3: What methodological approaches to assessing acceptability have researchers applied?

For methodological characteristics, the counts and proportions of quantitative, qualitative, and mixed‐methods studies were reported together with specifications of their methods. Numbers and frequencies of studies were summarised for temporal characteristics (acceptability assessed before or during/after screening).

## 3 Results

### 3.1 Study Characteristics

**FIGURE 1:**
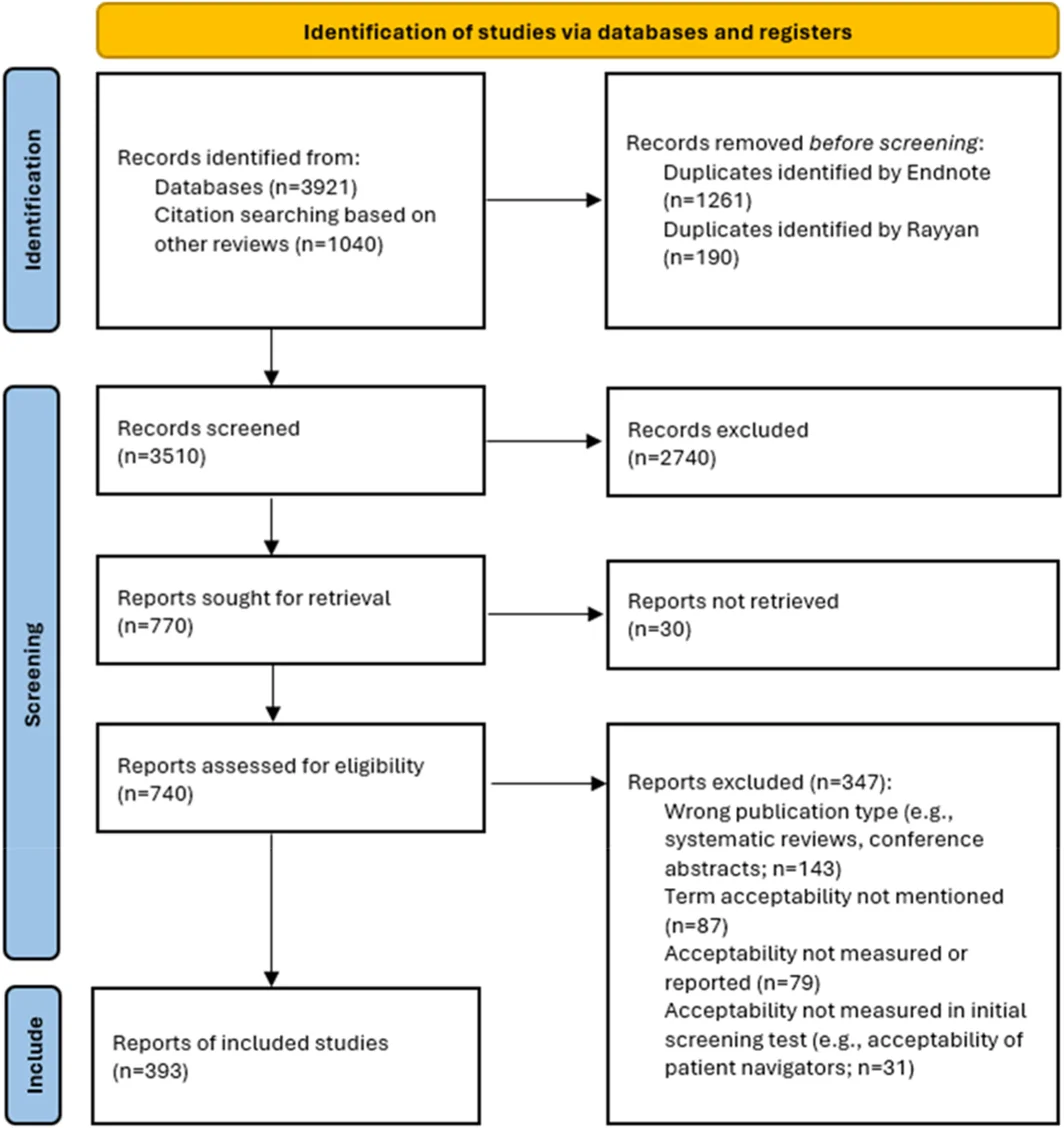
PRISMA flow diagram of study selection.

Of 3510 records screened, a total of 393 studies investigating acceptability in the context of cancer screening were included in the review (Figure 1). A full list of references (Supporting Information S1: Appendix A) and an overview of all included studies (Supporting Information S2: Appendix B) is provided.

Approximately half of the included studies were conducted in countries where English is the primary spoken language, including the United States (*n* = 74, 19.0%) and the United Kingdom (*n* = 56, 14.3%), followed by Australia (*n* = 30, 7.9%) and Canada (*n* = 22, 5.8%). Other commonly reported settings included Italy (*n* = 10, 2.4%), China and Uganda (each *n* = 9, 2.3%), Thailand and India (each *n* = 8, 2.1%), Cameroon, France, Nigeria and Tanzania (each *n* = 7, 1.8%), Mexico and Spain (each *n* = 6, 1.6%), and Brazil (*n* = 5, 1.3%). Seven studies were conducted across multiple countries (1.8%).

The included studies covered screening for a range of cancer types, with a majority addressing cervical screening (*n* = 248, 63.1%), of which *n* = 162 (65.3%) examined the acceptability of self‐sampling tests. Other cancer types included bowel, colon and colorectal (*n* = 45, 11.5%), breast (*n* = 38, 9.7%), anal (*n* = 15, 3.8%), lung (*n* = 12, 3.1%), upper gastrointestinal (*n* = 6, 1.5%), prostate (*n* = 6, 1.5%), skin (*n* = 5, 1.3%), ovarian (*n* = 5, 1.3%), endometrial (*n* = 3, 0.8%), head and neck (*n* = 3, 0.8%), and kidney (*n* = 3, 0.8%). Fifteen papers (3.8%) focussed on the acceptability of screening for more than one cancer type.

**FIGURE 2:**
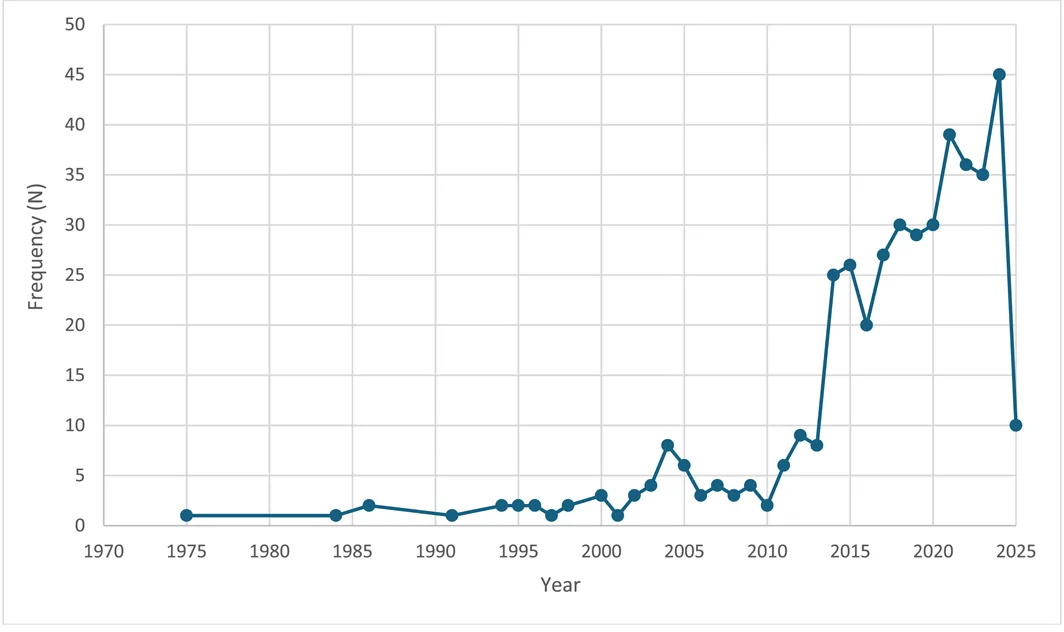
Frequency of studies published per year. Studies from 2025 only includes studies published until February 2025.

Included studies were published between 1975 and 2025, with a sharp increase in the annual number of publications since 2013 (Figure 2).

### 3.2 Study Populations

A full breakdown of the study characteristics is available in Supporting Information S2: Appendix B. Most studies focussed on acceptability within patients and public populations (*n* = 363, 92.4%; i.e. screening users or the general public). Studies also investigated acceptability among healthcare professionals (*n* = 55, 14.0%), policy makers (*n* = 10, 2.5%), researchers (*n* = 4, 1%) and other populations (*n* = 5, 1.3%). Forty‐three (10.9%) studies focussed on more than one population, for example, both healthcare professionals and patients and the public.

Overall, 68.4% of studies (269/393) explored acceptability in female participants (mostly for cervical screening) whilst only 14 studies (3.6%) focussed specifically on male participants (mostly for prostate cancer screening). A small number of studies focussed on other gender groups including transmasculine (*n* = 2), non‐binary (*n* = 1) and transgender men (*n* = 2). Around a quarter of studies (109; 27.7%) did not apply inclusion criteria for gender.

Studies investigated acceptability in a range of age groups aligning with age‐based screening eligibility criteria but also including those about to become eligible and those about to become ineligible for screening due to their age. Other studies explored acceptability across the adult population, for example from the age of 18 onwards. In 103 (26.2%) studies, the age of participants was not reported.

Some studies focussed on specific groups associated with inequalities in screening. Thirty‐nine studies specifically focussed on a certain ethnic group (9.9%), six focussed on people grouped by religious faith (1.5%), and 14 focussed on individuals living in economically deprived, disadvantaged and low‐income regions (3.5%).

### 3.3 Use of Theory to Inform Acceptability Research

Most studies (338/393; 86.0%) did not cite theory when exploring the acceptability of cancer screening. Among the 55 that did, 21 different theories were used, with 6/55 (10.9%) studies mentioning more than one model, theory or framework. Papers using theory cited individual/intrapersonal behaviour change theories (*n* = 36/55, 65.5%), implementation frameworks (*n* = 14/55, 25.5%), and other theories (*n* = 5/55, 9.1%). The most common single theory used was the Health Belief Model (HBM [25]; *n* = 15/55, 27.3%), followed by the Theoretical Framework of Acceptability (TFA^1^ [12]; *n* = 11/55, 20.0%) and the Consolidated Framework for Implementation Research (CFIR [26]; *n* = 8/55, 14.5%). A detailed breakdown of theory use is available in Table 1.

**TABLE 1:** Overview of theories used for acceptability research (among *n* = 55 studies using theory).

Theory^a^	*N*	%
Individual/intrapersonal behaviour change theories		
Health belief model (HBM)	15	27.3
Theoretical framework of acceptability (TFA)	11	20.0
Theory of planned behaviour (TPB)	4	7.2
Capability, opportunity, motivation, and behaviour (COM‐B)	2	3.6
Technology acceptance model (TAM)	1	1.8
Theoretical domains framework (TDF)	1	1.8
Social cognitive theory (SCT)	1	1.8
Self‐determination theory (SDT)	1	1.8
Learner verification and revision (LVR) model	1	1.8
Information motivation and behaviour model (IMB)	1	1.8
Extended parallel process model (EPPM)	1	1.8
Implementation frameworks		
Consolidated framework for implementation research (CFIR)	8	14.5
Proctor's outcomes for implementation research framework	4	7.2
Implementation outcomes framework (IOF)	1	1.8
Tailored implementation for chronic diseases checklist (TICD)	1	1.8
Diffusion of innovations theory	1	1.8
IMPaCT model	1	1.8
RE‐AIM framework	1	1.8
Other		
Socio‐ecological model (SEM)	2	3.6
Penchansky and Thomas theory of access	2	3.6
Thaddeus' and Maine's three‐delay model	1	1.8

^a^ some studies used more than one theory; hence the number of times each theory was used exceeds the overall number of studies that used theory.

### 3.4 Use of Theory Over Time

From 2020 to 2025, 41/193 (21.2%) studies cited a theory, representing an increase in use of theory compared to 2010 to 2019 (10/148, 6.8%), 2000 to 2009 (3/38, 7.9%), and 1990 to 1999 (1/10, 10.0%). No studies published before 1990 cited any theory, model or framework.

### 3.5 Definition and Operationalisation of Acceptability

All definitions used within papers included in the review are available in Supporting Information S1: Appendix A, Table S1. Only 17/393 (4.3%) studies provided a definition of acceptability within the text: six (6/17, 35.3%) defined acceptability according to the TFA [12], two (2/17, 11.8%) based on the Penchansky and Thomas' Access Framework [27], and two (2/7, 11.8%) based on Proctor's Outcomes for Implementation Research Framework [28]. Where theory was used, the definition of acceptability given in the study did not always align with the theory being cited. For example, one study cited the Penchansky and Thomas' Access Framework, which defines acceptability as “the relationship of clients' attitudes about personal and practice characteristics of providers to the actual characteristics of existing providers, as well as to provider attitudes about acceptable personal characteristics of clients” [29]. However, the study defined acceptability as “comfort with health care provider characteristics and perceptions of inferior treatment based on gender, ethnicity or social class” [30]. All six studies that used the TFA as a theoretical framework defined acceptability according to this theory, although one did not mention the multi‐component nature of acceptability per the TFA. Seven studies provided a definition of acceptability without reference to any theory or framework.

Content analysis of the 17 studies that provided a definition of acceptability grouped definitions into themes. Themes were not mutually exclusive in that some definitions were coded under several themes. The most common definitions of acceptability centred on perceptions and attitudes towards the screening test, including whether it is agreeable, cognitive/emotional reactions, and endorsement of the test (*n* = 11/17, 64.7%). Around a third of studies (*n* = 6/17; 35.3%) defined acceptability as a multi‐component construct, often based on the TFA. A small number of studies described acceptability as the willingness to undergo screening, or system and social factors (*n* = 3/17 each; 17.6%). One (*n* = 1/17; 5.9%) focussed on concepts around ethicality of the screening procedure independently from the TFA. A list of definitions and codes applied is available in Supporting Information S1: Appendix A, Table S1.

**FIGURE 3:**
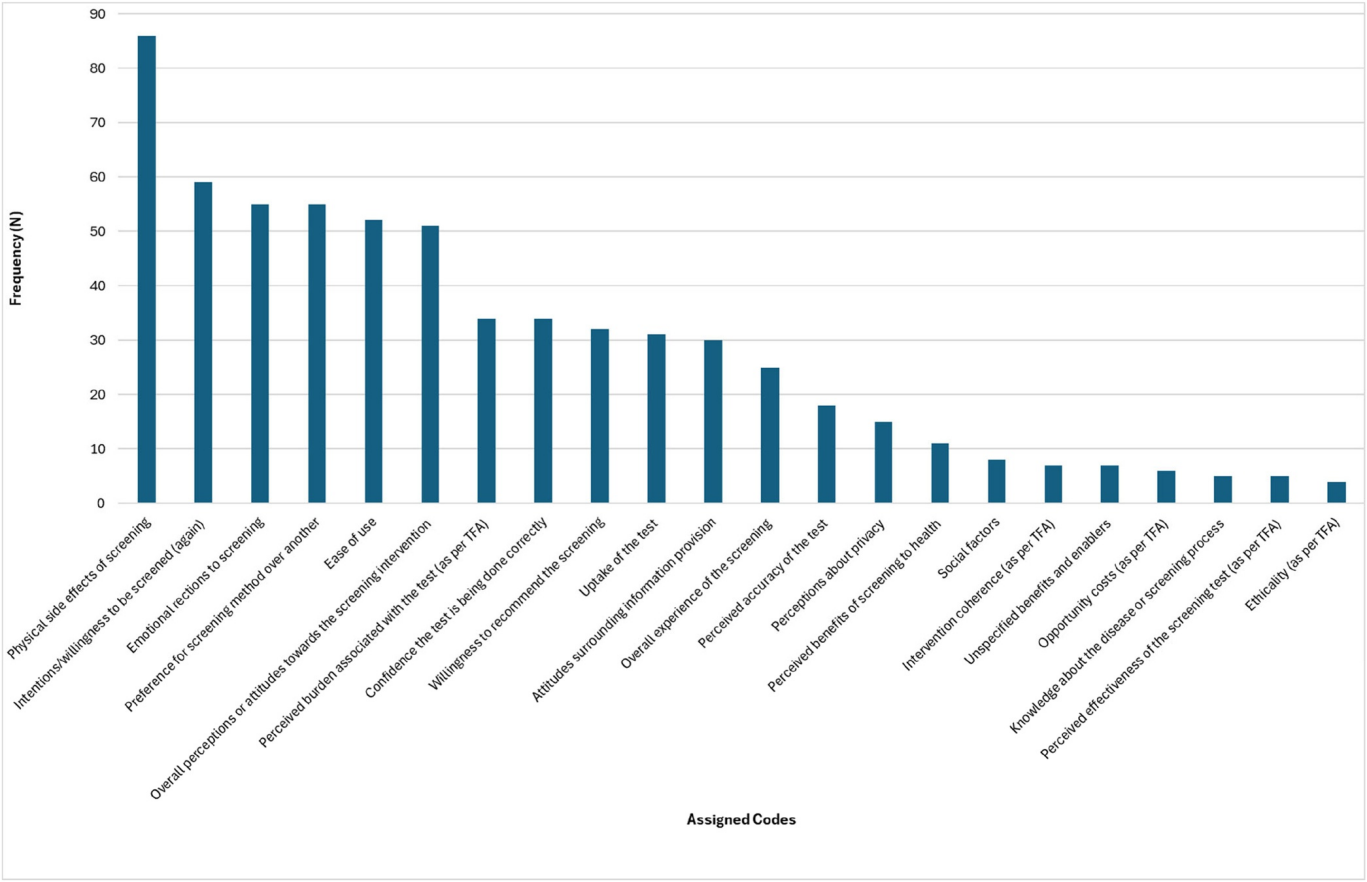
Frequency of assigned codes for operationalisations of acceptability (*n* = 201).

An overview of all operationalisations is provided in Supporting Information S1: Appendix A, Table S2, and in Figure 3. Whilst the use of theory and explicit definitions were infrequent, half of studies (201/393: 51.1%) provided information on how screening acceptability had been operationalised. These included measures such as uptake, as well as survey items that covered a broad range of related constructs. Using content analysis, the most frequent operationalisation centred around the physical side‐effects of screening, including pain and discomfort (*n* = 86/201, 42.8%). Between 20% and 30% of studies used aspects of acceptability such as willingness to be screened, emotional reactions such as fear and anxiety, screening test preferences (common in studies comparing self‐sampling to more traditional testing done by clinicians), ease of use, and perceptions and attitudes towards screening.

Fewer studies (between 10% and 20% for each operationalisation) assessed acceptability through measures of perceived burden or convenience of the screening; confidence about the screening being done correctly; willingness to recommend screening to friends, family members or others; attitudes and perceptions regarding the provided information; and experience of the screening. Thirty‐one studies (15.4%) simply used screening uptake as a marker of acceptability. Other operationalisations, for example, accuracy, unspecified benefits and enablers, social factors, and ethicality, were used in fewer than 10% of studies.

### 3.6 Methodology of Acceptability Research

Most studies used a quantitative approach to explore acceptability in cancer screening (*n* = 270/393, 68.7%) The remaining studies used qualitative approaches (*n* = 84/393, 21.4%) and mixed methods approaches (*n* = 39/393, 9.9%). Across all studies including mixed methods approaches, 278/393 investigated acceptability via surveys or questionnaires (70.7%) or reporting of uptake behaviour (e.g., using attendance records; *n* = 56/393, 14.2%). Fewer studies used qualitative methods such as interviews (*n* = 74/393, 18.8%) or focus groups (*n* = 57/393, 14.5%).

Most studies measured acceptability of cancer screening interventions during or after the intervention (*n* = 240, 61.1%). Almost a third of studies (122/393; 31.0%) measured the acceptability of hypothetical screening interventions before implementation, and 56 studies (14.2%) measured the acceptability of existing cancer screening interventions prior to a participant experiencing the intervention. A small proportion of studies (29/303; 7.4%) measured acceptability at more than one time point.

## 4 Discussion

This is the first study to have synthesised literature on what is measured when researchers explicitly used the term “acceptability” in cancer screening contexts. The number of studies (*N* = 393) synthesised in this scoping review underscores the growing importance of acceptability in cancer screening as a determinant of success alongside clinical effectiveness. Since 2013, research mentioning acceptability in the context of cancer screening has increased exponentially. Most studies examined patient or public perspectives, with healthcare professionals' views less often explored. Studies on acceptability of cervical screening dominated (63%), leading to more female than male participants across studies. Research on under‐screened groups was limited and mainly focussed on minoritised ethnic groups. Only 14% of studies referenced theoretical frameworks but these were seldom used to define acceptability, and explicit definitions were rare (4.6%), typically framing acceptability as a multidimensional construct or focussed on specific attitudes towards screening. About half of all studies described operationalisation of acceptability, predominantly through assessing physical side effects, emotional and cognitive responses, willingness to attend screening, and ease of use. Screening method preferences were commonly used as operationalisation in studies addressing cervical self‐sampling, indicating that operationalisation of the term “acceptability” likely varies across cancer types and tests. We suggest that robust and consistent definitions, operationalisations, and methodological standards are essential for future acceptability research in this context.

### 4.1 Integration With the Literature

Overall, acceptability was poorly conceptualised in studies that explicitly used the concept. Many studies conducted under the label “acceptability” did not provide an explicit definition or operationalisation of this construct. Several studies used physical side effects, willingness to be screened, emotional reactions, preferences for certain test modalities, ease of use, or general perceptions as a proxy for acceptability. The use of single measures limited to one facet may be too reductionist given that screening acceptability is influenced by multiple factors, including logistical barriers, social norms, health beliefs, and accessibility [31]. Similarly, the use of uptake does not provide sufficient information to fully understand all aspects of acceptability. Individuals may view cancer screening as acceptable but be unable to participate, while others may routinely participate despite finding it somewhat unacceptable (e.g., tolerating mammogram discomfort). Similarly, a large proportion of studies drew on consensus criteria for implementation such as screening‐related discomfort or physical side effects, outlined by Wilson and Jungner [32]. These operationalisations do not adequately capture the complexity of cancer screening acceptability.

Where acceptability was defined, definitions varied widely and incorporated broad constructs. This heterogeneity may reflect the diversity of disciplinary perspectives encompassed within this review, including behavioural science, implementation science, clinical and effectiveness trials, and feasibility studies. While this breadth of interest reflects the importance of acceptability research in cancer screening contexts, it underscores the need for an interdisciplinary conceptualisation. The lack of a unified conceptualisation or standard methods for assessment of acceptability in cancer screening research limits compatibility and the synthesis of findings [33]. Cancer screening researchers and evaluators are left to independently interpret the meaning of acceptability and develop methods for assessing acceptability for each new study [34]. A recent consensus survey among stakeholders of cancer screening acceptability found that differentiating acceptability from other implementation outcomes was the most frequently cited challenge, with 60% considering the lack of a standard definition for acceptability “very challenging” in this context [22].

The review's international scope may have further contributed to heterogeneity through linguistic and cultural variations. Terms such as “acceptability”, “acceptance”, or “tolerability” were often used interchangeably, highlighting the need for conceptual and terminological clarity [12, 35]. Furthermore, definitions of acceptability may vary across cultural contexts. For example, social acceptability of cancer screening may depend on family or spousal influence rather than individual choice [36, 37] or may vary across ethnic minority populations [38, 39].

Definitions of acceptability may be most useful when framed within theory [14]. Yet, the use of theory to guide the assessment of cancer screening acceptability was limited. When theories were cited, these tended to be models of health beliefs and behaviours or implementation theories, not specifically designed for evaluating acceptability [12, 22]. Although few theoretical frameworks explicitly address acceptability, the TFA is a notable exception. However, it was only cited by a small minority of studies and in many cases, it was unclear how it had been applied to research processes and outcomes. This gap between citation and application suggests limited theoretical engagement by those using the term “acceptability” when conducting related research in cancer screening contexts [40, 41], possibly due to the absence of a coherent, context‐specific definition and theoretical framework [22].

### 4.2 Implications (Clinical and Research)

Given the review findings and documented challenges experienced by those using the term “acceptability” to describe their research in the field of cancer screening, we propose that stakeholder consensus on a standardised definition of acceptability in cancer screening contexts is needed. This definition should encompass flexibility to accommodate the screening context and stakeholder perspectives. Although this may yield a high‐level definition, we believe that the consistent use of such a definition would advance the standardisation and transferability of acceptability research in cancer screening contexts. Furthermore, this will help to determine whether existing theoretical models of healthcare acceptability, such as the TFA, can be adapted to a cancer screening context. However, as this review indicates, there are multiple related constructs in the field of acceptability research that we did not explicitly identify and include, for example, intentions, preferences, or willingness to be screened. It is possible that research on these constructs has informed decision‐making about cancer screening, but this was not captured in this review. Therefore, researchers and stakeholders developing theory and consistent definitions of acceptability should review these related constructs and discuss their relationship with acceptability.

A theoretically informed definition of cancer screening acceptability may also improve reporting of acceptability research. Even when studies offered definitions and operationalisations of acceptability, these were often relegated to supplementary materials, indicating such measures may be viewed as peripheral to the aims and outcomes of clinical trials and effectiveness studies. Whilst the wealth of studies exploring acceptability, even as a secondary outcome, is a positive development, inadequate reporting standards may lead to the underutilisation of acceptability outcomes, as well as related constructs beyond the scope of this review.

To address the somewhat arbitrary conceptualisation and reporting of outcomes termed as “acceptability” highlighted in this review, we recommend reporting standards for research investigating the acceptability of cancer screening. These should include:

1. Inclusion of the term “acceptability” in the abstract and keywords when investigated within a study
2. Explicit definitions of acceptability in the methods section (ideally using a standardised, theory‐informed and consensus‐based definition), including boundaries and distinctions between the term “acceptability” and potentially related constructs
3. Where theory is used, researchers should define how this was applied to the assessment of acceptability in the methods section
4. Where theory is used, the usefulness of this theory should be reflected on within the discussion section

### 4.3 Strengths and Limitations

This large scoping review synthesised evidence from 393 studies that explicitly used the term “acceptability” to describe their outcomes. The scoping methodology was appropriate to examine how the term “acceptability” in cancer screening research has been measured and defined. Further methodological strengths include a systematic search strategy and independent screening by two authors. However, this review took a broad approach across cancer types and screening methods to capture overarching definitions and operationalisations of the term “acceptability” but did not examine these separately by cancer type or screening method (e.g., self‐sampling). As acceptability may be shaped by factors specific to a given test or cancer context, future research should differentiate between cancer types and screening methods to allow for a more nuanced understanding of acceptability in these contexts.

This review was limited in that we only looked at what researchers measured when using the term “acceptability” in regards to the screening tests and programmes themselves. There are several other elements of screening, including communication and behavioural interventions, that also influence participation. Limiting our review to the initial screening test or changes to the procedure and eligibility criteria disregards important interventions around screening, such as communication, that might improve acceptability. Future reviews considering other elements of screening would extend our understanding of how acceptability is conceptualised in screening contexts and provide a more holistic view of acceptability.

Additionally, we only included primary research studies. Authors of literature reviews might differ from authors of primary studies in how they define and operationalise acceptability, or they may include studies based on more elaborate search strategies. Literature reviews might be used more often by policymakers to inform evidence‐based decisions about screening programmes, and could include differing definitions of acceptability, not captured in this review. Hence, future research could examine how acceptability is defined and operationalised within literature reviews.

Restricting to English‐language publications may have reinforced Westernised perspectives on cancer screening acceptability and the exclusion of 30 inaccessible studies reduced coverage. Hence, definitions of acceptability in cancer screening that we found in this review are likely based on a non‐representative part of the population and could differ from theories used in other countries. Very few studies focussed specifically on acceptability within marginalised populations, even though screening uptake is often lower [38, 42]. This review did not identify any studies that considered screening acceptability in disabled populations, and very few were identified that focussed specially on other marginalised groups. Hence, future research is needed to specifically focus on acceptability in minority populations.

While the review benefitted from behavioural science expertise, acceptability is an interdisciplinary construct [12, 14, 22] and conceptualisations, definitions, and operationalisations may have been evaluated differently by other stakeholders.

As our objectives were to identify approaches for measuring acceptability when researchers explicitly stated acceptability was assessed, our inclusion criteria were restricted to articles including the term “acceptability” at least once. Despite this restriction, we have identified a high number of articles including diverse definitions and operationalisations. As a term‐based review, we decided not to include terms that could be seen as closely related to “acceptability”. This was done to avoid subjectivity and linguistical interpretation biasing our methodology. As such, this review excluded some research that could be classified as “acceptability” research despite not explicitly stating this. Future research could expand the search strategy to include additional related constructs such as intentions, willingness to be screened, and preferences for screening to further inform theory development.

A relatively high proportion of references to be screened (21%) was identified through alternative sources, that is, reference lists of reviews including potentially relevant articles. This number might be inflated as all references (including those from the background and discussion sections) of those reviews (*n* = 25) were included for title and abstract screening, rather than just the references that met the inclusion criteria of these reviews. We did not conduct a pre‐screening of titles at this stage, but directly uploaded all references.

## 5 Conclusions

Research using the term “acceptability” in cancer screening has largely been assessed without using theory, with few explicit definitions and varying operationalisations. The absence of a consensus‐based definition or reporting guidelines risks undermining evidence‐based decisions on screening programmes, with potential consequences for patients, professionals, and health systems. The identification or development of an interdisciplinary, flexible framework to guide how acceptability of cancer screening is defined and assessed should be prioritised.

## Author Contributions

L.G.: conceptualisation. L.G., N.S.‐B., C.D.: methodology. N.S.‐B., C.D.: formal analysis. N.S.‐B., C.D., C.K.J., P.R., R.E.C.E., E.K., E.L., L.M., S.H., A.T., A.B.A., A.V.E.P., L.G.: investigation. N.S.‐B., C.D., A.B.A., A.V.E.P.: data curation. N.S.‐B., C.D., C.K.J.: writing – original draft. R.E.C.E., E.K., E.L., L.M., S.H., A.T., S.E.S., J.W.: writing – review and editing. N.S.‐B.: visualization. J.W., S.E.S., L.G.: supervision. N.S.‐B., C.D., L.G.: project administration. S.E.S.: funding acquisition.

## Conflicts of Interest

The authors declare no conflicts of interest.

## Supporting information


Supporting Information S1

Supporting Information S2

## Data Availability

The authors have nothing to report.

## Endnotes

^1^
TFA: Theoretical Framework of Acceptability.
