# Supplementary material for: How has Acceptability Been Defined and Measured in Cancer Screening Research? A Scoping Review
Source: Psychooncology. 2026 Aug 20;35(8):e70578. doi: 10.1002/pon.70578 (PMC13492221; doi:10.1002/pon.70578)
Supplement: Supplementary file 1 [file PON-35-e70578-s001.docx]

# Preferred Reporting Items for Systematic reviews and Meta-Analyses extension for Scoping Reviews (PRISMA-ScR) Checklist

| **SECTION** | **ITEM** | **PRISMA-ScR CHECKLIST ITEM** | **REPORTED ON PAGE #** |
| --- | --- | --- | --- |
| **TITLE** | | | |
| Title | 1 | Identify the report as a scoping review. | 1 |
| **ABSTRACT** | | | |
| Structured summary | 2 | Provide a structured summary that includes (as applicable): background, objectives, eligibility criteria, sources of evidence, charting methods, results, and conclusions that relate to the review questions and objectives. | 2 |
| **INTRODUCTION** | | | |
| Rationale | 3 | Describe the rationale for the review in the context of what is already known. Explain why the review questions/objectives lend themselves to a scoping review approach. | 3-4 |
| Objectives | 4 | Provide an explicit statement of the questions and objectives being addressed with reference to their key elements (e.g., population or participants, concepts, and context) or other relevant key elements used to conceptualize the review questions and/or objectives. | 4 |
| **METHODS** | | | |
| Protocol and registration | 5 | Indicate whether a review protocol exists; state if and where it can be accessed (e.g., a Web address); and if available, provide registration information, including the registration number. | 4 |
| Eligibility criteria | 6 | Specify characteristics of the sources of evidence used as eligibility criteria (e.g., years considered, language, and publication status), and provide a rationale. | 5-6 |
| Information sources* | 7 | Describe all information sources in the search (e.g., databases with dates of coverage and contact with authors to identify additional sources), as well as the date the most recent search was executed. | 5 |
| Search | 8 | Present the full electronic search strategy for at least 1 database, including any limits used, such that it could be repeated. | Supplementary Material 1 |
| Selection of sources of evidence† | 9 | State the process for selecting sources of evidence (i.e., screening and eligibility) included in the scoping review. | 5-6 |
| Data charting process‡ | 10 | Describe the methods of charting data from the included sources of evidence (e.g., calibrated forms or forms that have been tested by the team before their use, and whether data charting was done independently or in duplicate) and any processes for obtaining and confirming data from investigators. | 6 |
| Data items | 11 | List and define all variables for which data were sought and any assumptions and simplifications made. | 6-7 |
| Critical appraisal of individual sources of evidence§ | 12 | If done, provide a rationale for conducting a critical appraisal of included sources of evidence; describe the methods used and how this information was used in any data synthesis (if appropriate). | N/A |
| Synthesis of results | 13 | Describe the methods of handling and summarizing the data that were charted. | 7 |
| **RESULTS** | | | |
| Selection of sources of evidence | 14 | Give numbers of sources of evidence screened, assessed for eligibility, and included in the review, with reasons for exclusions at each stage, ideally using a flow diagram. | 8 |
| Characteristics of sources of evidence | 15 | For each source of evidence, present characteristics for which data were charted and provide the citations. | 7 |
| Critical appraisal within sources of evidence | 16 | If done, present data on critical appraisal of included sources of evidence (see item 12). | N/A |
| Results of individual sources of evidence | 17 | For each included source of evidence, present the relevant data that were charted that relate to the review questions and objectives. | 7-14 |
| Synthesis of results | 18 | Summarize and/or present the charting results as they relate to the review questions and objectives. | 7-14 |
| **DISCUSSION** | | | |
| Summary of evidence | 19 | Summarize the main results (including an overview of concepts, themes, and types of evidence available), link to the review questions and objectives, and consider the relevance to key groups. | 14 |
| Limitations | 20 | Discuss the limitations of the scoping review process. | 16-17 |
| Conclusions | 21 | Provide a general interpretation of the results with respect to the review questions and objectives, as well as potential implications and/or next steps. | 16 |
| **FUNDING** | | | |
| Funding | 22 | Describe sources of funding for the included sources of evidence, as well as sources of funding for the scoping review. Describe the role of the funders of the scoping review. | 19 |

JBI = Joanna Briggs Institute; PRISMA-ScR = Preferred Reporting Items for Systematic reviews and Meta-Analyses extension for Scoping Reviews.

* Where *sources of evidence* (see second footnote) are compiled from, such as bibliographic databases, social media platforms, and Web sites.

† A more inclusive/heterogeneous term used to account for the different types of evidence or data sources (e.g., quantitative and/or qualitative research, expert opinion, and policy documents) that may be eligible in a scoping review as opposed to only studies. This is not to be confused with *information sources* (see first footnote).

‡ The frameworks by Arksey and O’Malley (6) and Levac and colleagues (7) and the JBI guidance (4, 5) refer to the process of data extraction in a scoping review as data charting*.*

§ The process of systematically examining research evidence to assess its validity, results, and relevance before using it to inform a decision. This term is used for items 12 and 19 instead of "risk of bias" (which is more applicable to systematic reviews of interventions) to include and acknowledge the various sources of evidence that may be used in a scoping review (e.g., quantitative and/or qualitative research, expert opinion, and policy document).

# Search strategy

The search was conducted on 14^th^ August 2023 and updated on 26^th^ February 2025.

**Ovid Medline (R)**

Acceptability.tw,kw. and ((*Mass Screenings/ and (neoplasm$ or tumo?r$ or cancer$).tw.) or *Early Detection of Cancer/ or *neoplasms/dg, di or cancer screening.kw or ((Cancer$ or tumo?r$ or neoplasm$) adj2 screening$).tw. or ((Early diagnosis or early detection) adj2 (neoplasm$ or tumo?r$ or cancer$)).tw. or (Screening tests adj6 early cancer).tw. or ((Cancer or tumo?r or neoplasm) adj detection).tw. or (cancer risk adj4 screening).tw.) or (((accept or accepted or acceptable or acceptance or accepting) adj7 ((Cancer$ or tumo?r$ or neoplasm$) adj2 screening$)) or ((accept or accepted or acceptable or acceptance or accepting) adj7 ((Early diagnosis or early detection) adj2 (neoplasm$ or tumo?r$ or cancer$))) or ((accept or accepted or acceptable or acceptance or accepting) adj7 ((Cancer or tumo?r or neoplasm) adj detection)) or ((accept or accepted or acceptable or acceptance or accepting) adj7 (cancer risk adj4 screening))).tw.

**APA PsycInfo**

Acceptability.ti,ab,id. and ((*"Cancer Screening"/ and (neoplasm* or tumo#r* or cancer*).ti,ab.) or *neoplasms/ or "cancer screening".id. or ((Cancer* or tumo#r* or neoplasm*) adj2 screening*).ti,ab. or (("Early diagnosis" or "early detection") adj2 (neoplasm* or tumo#r* or cancer*)).ti,ab. or ("Screening tests" adj6 "early cancer").ti,ab. or ((Cancer or tumo#r or neoplasm) adj detection).ti,ab. or ("cancer risk" adj4 screening).ti,ab.) or (((accept.ti,ab. OR accepted.ti,ab. OR acceptable.ti,ab. OR acceptance.ti,ab. OR accepting.ti,ab.) ADJ7 ((Cancer*.ti,ab. OR tumo#r*.ti,ab. OR neoplasm*.ti,ab.) ADJ2 screening*.ti,ab.)) OR ((accept.ti,ab. OR accepted.ti,ab. OR acceptable.ti,ab. OR acceptance.ti,ab. OR accepting.ti,ab.) ADJ7 (("Early diagnosis".ti,ab. OR "early detection".ti,ab.) ADJ2 (neoplasm#.ti,ab. OR tumo#r*.ti,ab. OR cancer*.ti,ab.))) OR ((accept.ti,ab. OR accepted.ti,ab. OR acceptable.ti,ab. OR acceptance.ti,ab. OR accepting.ti,ab.) ADJ7 ((Cancer.ti,ab. OR tumo#r.ti,ab. OR neoplasm.ti,ab.) ADJ detection.ti,ab.)) OR ((accept.ti,ab. OR accepted.ti,ab. OR acceptable.ti,ab. OR acceptance.ti,ab. OR accepting.ti,ab.) ADJ7 ("cancer risk".ti,ab. ADJ4 screening.ti,ab.)))

**Cochrane Central Register of Controlled Trials (CENTRAL)**

“Acceptability”:ti,ab,kw AND ([mh ^"Mass Screening"] AND (neoplasm*:ti,ab OR tumo?r*:ti,ab OR cancer*:ti,ab) OR "cancer screening":kw OR ((Cancer*:ti,ab OR tumo?r*:ti,ab OR neoplasm*:ti,ab) NEAR/2 screening*:ti,ab) OR (("Early diagnosis":ti,ab OR "early detection":ti,ab) NEAR/2 (neoplasm?:ti,ab OR tumo?r?:ti,ab OR cancer?:ti,ab)) OR ("Screening tests":ti,ab NEAR/6 "early cancer":ti,ab) OR ((Cancer:ti,ab OR tumo?r:ti,ab OR neoplasm:ti,ab) NEXT detection:ti,ab) OR ("cancer risk":ti,ab NEAR/4 screening:ti,ab)) OR “Acceptability”:ti,ab,kw AND ([mh ^neoplasms/di,dg] OR [mh ^"Early Detection of Cancer"]) OR (((accept:ti,ab OR accepted:ti,ab OR acceptable:ti,ab OR acceptance:ti,ab OR accepting:ti,ab) NEAR/7 ((Cancer*:ti,ab OR tumo?r*:ti,ab OR neoplasm*:ti,ab) NEAR/2 screening*:ti,ab)) OR ((accept:ti,ab OR accepted:ti,ab OR acceptable:ti,ab OR acceptance:ti,ab OR accepting:ti,ab) NEAR/7 (("Early diagnosis":ti,ab OR "early detection":ti,ab) NEAR/2 (neoplasm*:ti,ab OR tumo?r*:ti,ab OR cancer*:ti,ab))) OR ((accept:ti,ab OR accepted:ti,ab OR acceptable:ti,ab OR acceptance:ti,ab OR accepting:ti,ab) NEAR/7 ((Cancer:ti,ab OR tumo?r:ti,ab OR neoplasm:ti,ab) NEXT detection:ti,ab)) OR ((accept:ti,ab OR accepted:ti,ab OR acceptable:ti,ab OR acceptance:ti,ab OR accepting:ti,ab) NEAR/7 ("cancer risk":ti,ab NEAR/4 screening:ti,ab)))

**CINAHL (EBSCO)**

(TX acceptability) AND ((MM "Early Detection of Cancer") OR (MM neoplasms) OR (MM “cancer screening”) OR (((TI Cancer* OR AB Cancer*) OR (TI tumo#r* OR AB tumo#r*) OR (TI neoplasm* OR AB neoplasm*)) N2 (TI screening* OR AB screening*)) OR (((TI "Early diagnosis" OR AB "Early diagnosis") OR (TI "early detection" OR AB "early detection")) N2 ((TI neoplasm* OR AB neoplasm*) OR (TI tumo#r* OR AB tumo#r*) OR (TI cancer* OR AB cancer*)))) OR ((TI "Screening tests" OR AB "Screening tests") N6 (TI "early cancer" OR AB "early cancer")) OR (TX Acceptability) AND ((TI “cancer detection OR AB “cancer detection OR TI “tumo#r detection” OR AB “tumo#r detection” OR TI “neoplasm detection” OR AB “neoplasm detection”) OR ((TI "cancer risk" OR AB "cancer risk") N4 (TI screening OR AB screening))) OR ((((TI accept OR AB accept) OR (TI accepted OR AB accepted) OR (TI acceptable OR AB acceptable) OR (TI acceptance OR AB acceptance) OR (TI accepting OR AB accepting)) N7 (((TI Cancer* OR AB Cancer*) OR (TI tumo#r* OR AB tumo#r*) OR (TI neoplasm* OR AB neoplasm*)) N2 (TI screening* OR AB screening*))) OR (((TI accept OR AB accept) OR (TI accepted OR AB accepted) OR (TI acceptable OR AB acceptable) OR (TI acceptance OR AB acceptance) OR (TI accepting OR AB accepting)) N7 (((TI "Early diagnosis" OR AB "Early diagnosis") OR (TI "early detection" OR AB "early detection")) N2 ((TI neoplasm* OR AB neoplasm*) OR (TI tumo#r* OR AB tumo#r*) OR (TI cancer* OR AB cancer*)))) OR (((TI accept OR AB accept) OR (TI accepted OR AB accepted) OR (TI acceptable OR AB acceptable) OR (TI acceptance OR AB acceptance) OR (TI accepting OR AB accepting)) N7 (((TI Cancer OR AB Cancer) OR (TI tumo#r OR AB tumo#r) OR (TI neoplasm OR AB neoplasm)) W1 (TI detection OR AB detection))) OR (((TI accept OR AB accept) OR (TI accepted OR AB accepted) OR (TI acceptable OR AB acceptable) OR (TI acceptance OR AB acceptance) OR (TI accepting OR AB accepting)) N7 ((TI "cancer risk" OR AB "cancer risk") N4 (TI screening OR AB screening))))

**Web of Science Core Collection (Science Citation Index Expanded [1900-present] and Social Sciences Citation Index [1956-present])**

(AK=acceptability OR TI=acceptability OR AB=acceptability) AND ((TS="mass screening*" AND (TI=neoplasm* OR TI=tumo$r* OR TI=cancer*) OR (AK="Cancer screening") OR (TI=((Cancer* OR tumo$r* OR neoplasm*) NEAR/2 screening*)) OR AB=((Cancer* OR tumo$r* OR neoplasm*) NEAR/2 screening?)) OR (TI=(("Early diagnosis" OR "early detection") NEAR/2 (neoplasm? OR tumo$r? OR cancer?)) OR AB=(("Early diagnosis" OR "early detection") NEAR/2 (neoplasm? OR tumo$r? OR cancer?))) OR (TI=("Screening tests" NEAR/6 "early cancer")) OR (AB=("Screening tests" NEAR/6 "early cancer")) OR (TI=("cancer risk" NEAR/4 screening)) OR AB=("cancer risk" NEAR/4 screening) OR (AK=acceptability OR TI=acceptability OR AB=acceptability) AND (TI=((Cancer OR tumo$r OR neoplasm) NEAR/0 detection) OR AB=((Cancer OR tumo$r OR neoplasm) NEAR/0 detection))) OR (TI=(((accept OR accepted OR acceptable OR acceptance OR accepting) NEAR/7 ((Cancer? OR tumo$r? OR neoplasm?) NEAR/2 screening?)) OR ((accept OR accepted OR acceptable OR acceptance OR accepting) NEAR/7 (("Early diagnosis" OR "early detection") NEAR/2 (neoplasm? OR tumo$r? OR cancer?))) OR ((accept OR accepted OR acceptable OR acceptance OR accepting) NEAR/7 ((Cancer OR tumo$r OR neoplasm) NEAR/0 detection)) OR ((accept OR accepted OR acceptable OR acceptance OR accepting) NEAR/7 ("cancer risk" NEAR/4 screening))) OR AB=(((accept OR accepted OR acceptable OR acceptance OR accepting) NEAR/7 ((Cancer? OR tumo$r? OR neoplasm?) NEAR/2 screening?)) OR ((accept OR accepted OR acceptable OR acceptance OR accepting) NEAR/7 (("Early diagnosis" OR "early detection") NEAR/2 (neoplasm? OR tumo$r? OR cancer?))) OR ((accept OR accepted OR acceptable OR acceptance OR accepting) NEAR/7 ((Cancer OR tumo$r OR neoplasm) NEAR/0 detection)) OR ((accept OR accepted OR acceptable OR acceptance OR accepting) NEAR/7 ("cancer risk" NEAR/4 screening))))

# Data extraction sheet

| **Item** | **Response Options** **(extractor selected all that apply)** | **Follow up questions (if applicable)** |
| --- | --- | --- |
| Name of Paper |  |  |
| Sample sizes | # |  |
| What population(s) is acceptability being studied in? | Patient and public  Family members or relatives of patient  Informal carer of family (family members or relatives)  Another group acting on behalf of the patient (see question below)  Healthcare Professionals (nurses, doctors, formal carer of patient)  Policy maker - hospital  Policy maker - government  Researchers  Other - please state below | If other – state below |
| Did this study look at a particular population sex? | Yes/No | If yes, which sex? |
| Did this study look at a particular ethnic group or community? | Yes/No | If yes: what ethnic group/community was focussed on? |
| Did this study look at a particular age population? If yes: | Yes/No | If yes: What age range did the study specifically focus on? |
| Is acceptability defined in the text? | Yes/No | If yes: Definition of acceptability (free text) |
| Does the paper refer to any acceptability theories, models or frameworks? | Yes/No | If yes: Number of acceptability theories, models and/or frameworks referred to [#]  If yes: Which theory, model, or frameworks are mentioned? (Tick all that apply”)  If other: "Please specify which other theories, models and/or frameworks are mentioned:  If TFA: If the authors said they are using TFA concepts, which of these have they said they are using (Select from elements) |
| What type of acceptability are the authors capturing in the study? | Affective  Cognitive  Behavioural    Definitions:  Affective: expressing emotions and these being interpreted by the researchers as an indicator of acceptability, for example expressing fear, disgust, embarrassment  Cognitive: agreeing to statements or providing rationalisations relating to acceptability  Behavioural: carrying out a behaviour which is interpreted as indicating acceptability, e.g., attendance/attending screening |  |
| According to the authors, what context is acceptability being measured in? | Public acceptability  Individual or personal acceptability  Social acceptability  Financial acceptability  Other- see below  No specific context given |  |
| What are the temporal characteristics of acceptability being investigated? | Hypothetical 'intervention'  Prior to 'intervention'  During or after 'intervention' |  |
| What type of method was used for data collection? | Quantitative  Qualitative  Mixed |  |
| What qualitative data collection method(s) were used? | Interviews  Survey  Focus groups  Observational studies  Polls  Other | If other: what other? |
| What type of quantitative data collection method(s) were used? | Survey  Attendance records  Other | If other: what other? |
| If a quantitative measure was used, which were used? | Open text | If other: please state |
| (as above for quantitative/qualitative for mixed methods studies) | | |

# Table S1: Overview of definitions of acceptability

| **First author (year)** | **Definition of acceptability** | **Coded as** | **Theory used?** |
| --- | --- | --- | --- |
| *Adamsen et al., (1984)* | "that is, the willingness to undergo screening" | Willingness to be screened | None |
| *Akinyemiju et al., (2015)* | "acceptability refers to comfort with health care provider characteristics and perceptions of inferior treatment based on gender, ethnicity or social class" | System and social factors; ethicality | Penchansky and Thomas framework |
| *Aleshire et al., (2020)* | "Acceptability refers to the relationship of the patient’s and health care provider’s attitudes, preferences, and characteristics related to each other (e.g., age, gender, ethnicity, years of practice, comorbid conditions) and to the health care setting (e.g., type of facility, religious affiliation)." | System and social factors; | Penchansky and Thomas framework |
| *Alvarex-Gonzalez et al., (2019)* | "patient's willingness to use the same bowel preparation protocol in a hypothetical new procedure" | Intention/willingness to be screened | None |
| *Bakiewicz et al (2020)* | "By acceptability, we understand women’s perception/attitude towards HPV self-sampling" | Perceptions/attitudes | None |
| *Colling et al., (2024)* | Used the definitions for each of the seven domains in the TFA per Sekhon, 2017 | Multi-component construct | TFA (2017) |
| *Dunlop et al., (2021)* | "Acceptability in this setting refers to the perception that the use of additional risk factors and tailored screening advice is agreeable and in the target population's interest" | Perceptions/attitudes | Proctor Outcomes for Implementation Research framework |
| *Dunlop et al., (2024)* | "Acceptability refers to the perception that a change in practice is agreeable and in the target population’s interest" | Perceptions/attitudes | None |
| *Dunlop et al (2023)* | "Acceptability (i.e., satisfaction with various aspects of the innovation)" with seven dimensions: Guideline factors, individual health professional factors, patient factors, professional interactions, incentives and resources, capacity for organisational change, social, political and legal factors | Multi-component construct; system and social factors | Proctor Outcomes for Implementation Research framework |
| *Hill et al., (2020)* | "A multi-faceted construct which reflects the extent to which people delivering or receiving a healthcare intervention consider it to be appropriate, based on anticipated or experienced cognitive and emotional responses" | Multi-component construct; perceptions/attitudes | TFA (2017) |
| *Jenkinson et al., (2023)* | Definition of acceptability proposed by Sekhon et al [33]: “a multi-faceted construct that reflects the extent to which people receiving a healthcare intervention consider it to be appropriate, based on anticipated or experienced cognitive and emotional responses to the intervention.” | Multi-component construct; perceptions/attitudes | TFA (2017) |
| *Kaufman et al., (2020)* | "We defined acceptability as the extent to which individuals who experienced standardized screening procedures endorsed the integration of the same procedures into routine care" | Perceptions/attitudes | None |
| *Marlow et al., (2022)* | Acceptability “reflects the extent to which people delivering or receiving a healthcare intervention consider it to be appropriate, based on anticipated or experienced cognitive and emotional responses” | Perceptions/attitudes | TFA (2017) |
| *Mremi et al., (2021)* | "By acceptability, we mean the women's perception/attitude towards the HPV self-sampling procedure"; understanding of instructions, difficulty, experience of bleeding, pain, worry to hurt oneself, confidence in collecting sample, discomfort or embarrassment, worry about swab being unsafe/ contaminated, worry about dropping brush, recommendation to friend/ family, recommendation to use brush | Perceptions/attitudes | None |
| *Němec et al., (2022)* | "Multifaceted construct which reflects the extent to which people delivering or receiving a healthcare intervention consider it to be appropriate, based on anticipated or experienced cognitive and emotional responses" | Multi-component construct; perceptions/attitudes | TFA (2017) |
| *Tonge et al., (2018)* | ""Acceptability" was considered as positivity about the service in principle and expressed intent for use." | Perceptions/attitudes; intention/willingness to be screened | None |
| *Usher-Smith et al., (2024)* | "Acceptability is a multi-faceted construct that reflects the extent to which people delivering or receiving a healthcare intervention consider it to be appropriate, based on anticipated or experienced cognitive and emotional responses to the interventions" | Multi-component construct; perceptions/attitudes | TFA (2017) |

# Table S2: Overview of operationalisation of acceptability

| **Code^1^** | **N (%) of assigned codes (N=201 studies providing operationalisation)** | **Description** | **Example^2^** |
| --- | --- | --- | --- |
| Physical side effects of screening test | 86 (42.8%) | Measures assessing pain (n=54) and discomfort (n=64) caused by the screening intervention, as well as other physical reactions to the test (n=7), for example, bleeding, gagging, retching, the need for sedation and safety of the procedure. | “The questions on the acceptability were on the difficulties of sampling. The options were very easy, easy or difficult. In addition, the **participants were asked whether they experienced pain or bleeding** and if they were comfortable doing it” (Nairan et al., 2018). |
| Intentions/willingness to be screened (again) | 59 (29.4%) | Questions focusing on participants’ intentions to have the intervention, either in the future, or willingness to repeat screening. | “To capture overall acceptability, we assessed ease and comfort of using the self-swab and **willingness to use it again”** (Kohler et al., 2019). |
| Emotional reactions to screening | 55 (27.4%) | Emotional reactions to the screening programme, including fear, worry and anxiety (n=33), relaxation (n=7) and embarrassment (n=33). | "By acceptability, we mean the women's perception/attitude towards the HPV self-sampling procedure"; understanding of instructions, difficulty, experience of bleeding, pain, **worry to hurt oneself**, confidence in collecting sample, discomfort or embarrassment, **worry about swab being unsafe/ contaminated, worry about dropping brush**, recommendation to friend/ family, recommendation to use brush” (Mremi et al., 2021). |
| Preference for one screening method over another | 55 (27.4%) | Participants were asked to indicate a preference for one intervention over another, and the reasons given for preferences. | “Respondents were asked to rate the acceptability of each method using a five point Likert scale (‘very acceptable’ to ‘very unacceptable’) and **asked to state which of the three methods they thought was the best”** (Ellis et al., 2007). |
| Ease of use | 52 (25.9%) | Participants were asked how easy it was to do the intervention and things that made the intervention difficult. | “Participants were asked a series of closed-ended questions regarding different aspects of acceptability, including understanding of instructions, **ease of use,** degree of pain and bleeding associated with use, and willingness to use self-sample HPV testing regularly” (Montealegre et al., 2014). |
| Overall perceptions or attitudes towards the screening intervention | 51 (25.4%) | Measures that tried to capture how participants felt about an intervention overall or how satisfied they were with their decision. Included terms such as "acceptability", "impressions", "perceptions", "attitudes", "satisfaction", reasonable". | “The first was **a question on the overall acceptability of LCS [lung cancer screening],** namely how strongly they were in favor of or against the screening process” (Djruic et al., 2023). |
| Perceived burden associated with the test (as per TFA) | 34 (16.9%) | Perceived amount of effort required to take part in the intervention, studies measuring burden associated with screening, namely the convenience of screening (n=28). | “Participants were then asked to rate both test on characteristics of ease, pain, privacy, embarrassment, confidence that the test is performed properly, accuracy of the test, fear of harm, and **convenience**. This was then used to compute on overall acceptability score for each test. Each question for acceptability was rated on a scale of 1 to 4 (less to more)” (Molokwu et al., 2018). |
| Confidence the test is being done correctly | 34 (16.9%) | Included measures assessing self-efficacy to complete screening (especially in self-sampling), confidence in healthcare professionals' ability to perform the test and overall confidence. | “The second part assessed the acceptability of HPV self-sampling which included five indices: easy and convenient to perform, less embarrassment, not painful, **confidence in performing the procedure**, and would recommend self-sampling to a friend or relative.” |
| Willingness to recommend the screening | 32 (15.9%) | Measures asking participants about their willingness to recommend the intervention to a friend, family member, or others generally. | "Given the same medical circumstances, **would you advise a friend to undergo this test**?" (Ploysawang et al., 2023) |
| Uptake of the test | 31 (15.4%) | Studies where the number of people having, repeating or deciding not to have an intervention was used to assess acceptability, either self-reported or recorded in health records. | "a) the **proportion of women that returned the self-sample after one month of the screening visit** by trial arm " (Ibáñez et al., 2023) |
| Attitudes surrounding information provision | 30 (14.9%) | Included perceptions around the clarity of the instructions, how well the instructions were understood or whether there were difficulties understanding the instructions, satisfaction with the information that was provided, and how helpful instructions were. | “The acceptability of the screening procedure was measured using a series of questions about the waiting time, discomfort experienced during the test and the provision of privacy and **adequate explanation during the procedure**. (Seow et al., 1998).” |
| Overall experience of the screening | 25 (12.4%) | Measures asking participants about their specific experiences of the screening procedure. | “Participants' subjective feelings of the sampling process were evaluated using a visual analogue scale (VAS) shortly after the procedure and before endoscopy. **This scale has a range of 1–10, with higher scores indicating better experience and vice versa,** and a cartoon face was used to assist interpretation of this scale” (Gao et al., 2021). |
| Perceived accuracy of the test | 18 (9.0%) | Studies focusing on (beliefs about) the reliability, accuracy or trust in the results of cancer screening. | “Participants were then asked to rate both test on characteristics of ease, pain, privacy, embarrassment, confidence that the test is performed properly, **accuracy of the test**, fear of harm, and convenience. This was then used to compute on overall acceptability score for each test” (Molokwu et al., 2018). |
| Perceptions about privacy | 15 (7.5%) | Studies assessing acceptability by investigating respondents’ perceptions of privacy and invasiveness of the test. | "Acceptability measures included … **how well the respondents’ privacy was handled during the tests**" (Madhivinan et al., 2021). |
| Perceived benefits of screening to health | 11 (5.5%) | Studies that asked participants about the perceived benefits of screening to their health including perceived necessity (“I need to do it”), usefulness of screening and motivations to look after health through screening. | Acceptability was measured in a survey including items measuring constructs such as: ease of sample taking, perceived success of sample taking, discomfort from the procedure, embarrassment from the procedure, trust in the test result, willingness to recommend the test to a friend, **perceptions that the screening test is useful and would prevent cancer,** satisfaction with the user manual and information needs being met (Karjalainen et al., 2016). |
| Social factors | 8 (4.0%) | Included satisfaction with professional interactions, social support for screening decisions, subjective norms, need for help from others, and religious influence. | Under outcome measures: “Acceptability—(1) proportion satisfied with their initial visit decisions; **(2) proportion whose partner supported their treatment decisions**; and (3) proportion who successfully adhered to home-care instructions.” (Gaffikin et al., 2003). |
| Intervention coherence (as per TFA) | 7 (3.5%) | Participants’ understanding of how the test works, or why the test is beneficial. | Topic guide had questions surrounding intervention coherence such as: “**Do you feel it is clear to you why risk-based screening might be beneficial? Explain briefly; Do you agree that women with a higher risk of cervical cancer should be tested more frequently? Why?; Do you agree that women with a lower risk of cervical cancer should be tested less frequently? Why?”** (Remmel et al., 2024). |
| Unspecified benefits and enablers | 7 (3.5%) | Studies where participants were asked about the barriers and enablers of screening but the nature of these barriers and enablers were not specified. | "Acceptability is inferred to be willingness to engage in self-sampling HVP tests which may be determined by knowledge &/beliefs, perceived susceptibility & severity, **perceived benefits and barriers**" (Scarinci et al., 2013). |
| Opportunity costs (as per TFA) | 6 (3.0%) | The extent to which benefit, profits or values must be given up to engage in the intervention, including monetary costs. | Asked participants questions about their attitudes to self-sampling including whether it is: “more private”, “more convenient”, “easier to operate”, **“less costly”** and “less painful” (Li et al., 2022). |
| Knowledge about the disease or screening process | 5 (2.5%) | Studies assess acceptability through measures of screening or disease knowledge and awareness. | “This study designed its own questionnaire on the level of awareness, experience, acceptability and preference for self-sampling of HPV, which consisted of four parts with a total of 42 entries… The second section (Questions 16 to 22) addressed **the level of awareness about cervical cancer screening and HPV self-sampling”** (Song et al., 2024). |
| Perceived effectiveness of the screening test (as per TFA) | 5 (2.5%) | The extent to which the intervention is perceived as likely to achieve its purpose. | “Questions based on Theoretical Framework of Acceptability constructs namely: affective attitude, burden, **perceived effectiveness**, ethicality, intervention coherence, opportunity costs, and self-efficacy were administered to eligible participants” (Ninsiima et al., 2023). |
| Other | 5 (2.5%) | Willingness to pay, near-patient testing, capacity for organisational change, guideline factors. | Acceptability (i.e., satisfaction with various aspects of the innovation) **with seven dimensions: Guideline factors, individual health professional factors, patient factors, professional interactions, incentives and resources, capacity for organisational change, social, political and legal factors (**Dunlop et al., 2023). |
| Ethicality (as per the TFA) | 4 (2.0%) | The extent to which the intervention has good fit with an individual's value system. | “Questions based on Theoretical Framework of Acceptability constructs namely: affective attitude, burden, perceived effectiveness, **ethicality**, intervention coherence, opportunity costs, and self-efficacy were administered to eligible participants” (Ninsiima et al., 2023). |

^1^Data from content analysis conducted on all studies where an operationalisation of acceptability was provided.

^2^ Where quotation marks are used, there are direct quotes from the paper. Otherwise, the authors of the review paraphrased operationalisations.

# Full list of included studies

Abotchie, P.N. and Shokar, N.K. (2009). Cervical Cancer Screening Among College Students in Ghana: Knowledge and Health Beliefs. *International Journal of Gynecologic Cancer*, 19(3), pp.412–416. <https://doi.org/10.1111/igc.0b013e3181a1d6de>.

Abdullah, N. N., Daud, S., Wang, S. M., Mahmud, Z., Mohd Kornain, N. K., & Al-Kubaisy, W. (2018). Human Papilloma Virus (HPV) self-sampling: do women accept it?. *Journal of obstetrics and gynaecology*, *38*(3), 402–407. <https://doi.org/10.1080/01443615.2017.1379061>

Adamsen, S. and Kronborg, O. (1984). Acceptability and Compliance in Screening for Colorectal Cancer with Fecal Occult Blood Test. *Scandinavian Journal of Gastroenterology*, 19(4), pp.531–534. https://doi.org/10.1080/00365521.1984.12005765.

Adamson, P.C., Huchko, M.J., Moss, A.M., Kinkel, H.F. and Medina-Marino, A. (2015). Acceptability and Accuracy of Cervical Cancer Screening Using a Self-Collected Tampon for HPV Messenger-RNA Testing among HIV-Infected Women in South Africa. *PLOS ONE*, 10(9), p.e0137299. https://doi.org/10.1371/journal.pone.0137299.

Adcock, A., Cram, F., Lawton, B., Geller, S., Hibma, M., Sykes, P., MacDonald, E.J., Dallas‐Katoa, W., Rendle, B., Cornell, T., Mataki, T., Rangiwhetu, T., Gifkins, N. and Hart, S. (2019). Acceptability of self‐taken vaginal HPV sample for cervical screening among an under‐screened indigenous population. *Australian and New Zealand Journal of Obstetrics and Gynaecology*, [online] 59(2), pp.301–307. https://doi.org/10.1111/ajo.12933.

Adefuye, P.O., Dada, O.A., Adefuye, B.O., Shorunmu, T.O., Akinyemi, B.O. and Idowu-Ajiboye, B.O. (2014). Feasibility, acceptability, and effectiveness of visual inspection of the cervix with acetic acid and cryotherapy for dysplasia in Nigeria. *International Journal of Gynecology & Obstetrics*, 129(1), pp.62–66. https://doi.org/10.1016/j.ijgo.2014.10.032.

Adegboyega, A., Desmennu, A.T. and Dignan, M. (2021). Qualitative assessment of attitudes toward cervical cancer (CC) screening and HPV self-sampling among African American (AA) and Sub Saharan African Immigrant (SAI) women. *Ethnicity & Health*, pp.1–18. https://doi.org/10.1080/13557858.2021.1980771.

Adekunle, T.B., Arreola, A., Sathveka Sembian, Castro, R., Claure, L., Balian, L. and Rodriguez, N.M. (2023). Feasibility and anticipated acceptability of community health worker-facilitated HPV self-sampling for cervical cancer screening around Lake County, Indiana. *Journal of clinical and translational science*, 7(1). https://doi.org/10.1017/cts.2023.578.

Akinyemiju, T.F., McDonald, J.A. and Lantz, P.M. (2015). Health care access dimensions and cervical cancer screening in South Africa: analysis of the world health survey. *BMC Public Health*, 15(1). https://doi.org/10.1186/s12889-015-1686-5.

Alatise, O. I., Dare, A. J., Akinyemi, P. A., Abdulkareem, F. B., Olatoke, S. A., Knapp, G. C., ... & Tulloch, R. (2022). Colorectal cancer screening with fecal immunochemical testing: a community-based, cross-sectional study in average-risk individuals in Nigeria. The Lancet Global Health, 10(7), e1012-e1022. doi: <https://doi.org/10.1016/S2214-109X(22)00121-8>

Aleshire, M.E., Adegboyega, A., Escontrías, O.A., Edward, J. and Hatcher, J. (2020). Access to Care as a Barrier to Mammography for Black Women. *Policy, Politics, & Nursing Practice*, 22(1), p.152715442096553. https://doi.org/10.1177/1527154420965537.

Allende, G., Surriabre, P., Cáceres, L., Bellot, D., Ovando, N., Torrico, A., Calle, P., Ascarrunz, C., Alexander, S., Bossens, M., Fontaine, V. and Rodríguez, P. (2019). Evaluation of the self-sampling for cervical cancer screening in Bolivia. *BMC Public Health*, [online] 19(1). https://doi.org/10.1186/s12889-019-6401-5.

Almog, R., Ezra, G., Lavi, I., Rennert, G. and Hagoel, L. (2008). The public prefers fecal occult blood test over colonoscopy for colorectal cancer screening. *European Journal of Cancer Prevention*, 17(5), pp.430–437. https://doi.org/10.1097/cej.0b013e328305a0fa.

Alvarez-Gonzalez, M.A., Pantaleon, M.A., Flores-Le Roux, J.A., Zaffalon, D., Amorós, J., Bessa, X., Seoane, A. and Pedro-Botet, J. (2019). Randomized Clinical Trial: A Normocaloric Low-Fiber Diet the Day Before Colonoscopy Is the Most Effective Approach to Bowel Preparation in Colorectal Cancer Screening Colonoscopy. *Diseases of the Colon & Rectum*, 62(4), pp.491–497. https://doi.org/10.1097/dcr.0000000000001305.

Anderson, C., Breithaupt, L., Des Marais, A., Rastas, C., Richman, A., Barclay, L., Brewer, N.T. and Smith, J.S. (2017). Acceptability and ease of use of mailed HPV self-collection among infrequently screened women in North Carolina. *Sexually Transmitted Infections*, [online] 94(2), pp.131–137. https://doi.org/10.1136/sextrans-2017-053235.

Anhang, R., Nelson, J.M., Telerant, R., Mary Ann Chiasson and Wright, T.W. (2005). Acceptability of Self-Collection of Specimens for HPV DNA Testing in an Urban Population. 14(8), pp.721–728. https://doi.org/10.1089/jwh.2005.14.721.

Ansink, A.C., Tolhurst, R., Haque, R., Saha, S., Datta, S. and van den Broek, N.R. (2008). Cervical cancer in Bangladesh: community perceptions of cervical cancer and cervical cancer screening. *Transactions of the Royal Society of Tropical Medicine and Hygiene*, 102(5), pp.499–505. https://doi.org/10.1016/j.trstmh.2008.01.022.

Arriba, L.N., Enerson, C.L., Belinson, S., Novick, L. and Belinson, J. (2010). Mexican Cervical Cancer Screening Study II: acceptability of human papillomavirus self-sampler. *International Journal of Gynecological Cancer*, 20(8), pp.1415–23. https://doi.org/10.1111/igc.0b013e3181f58678.

Arrossi, S., Ramos, S., Straw, C., Thouyaret, L. and Orellana, L. (2016). HPV testing: a mixed-method approach to understand why women prefer self-collection in a middle-income country. *BMC Public Health*, 16(1). https://doi.org/10.1186/s12889-016-3474-2.

Atkin, W., Cross, A.J., Kralj-Hans, I., MacRae, E., Piggott, C., Pearson, S., Wooldrage, K., Brown, J., Lucas, F., Prendergast, A., Marchevsky, N., Patel, B., Pack, K., Howe, R., Skrobanski, H., Kerrison, R., Swart, N., Snowball, J., Duffy, S.W. and Morris, S. (2019). Faecal immunochemical tests versus colonoscopy for post-polypectomy surveillance: an accuracy, acceptability and economic study. *Health Technology Assessment*, 23(1), pp.1–84. https://doi.org/10.3310/hta23010.

Audet, C.M., Matos, C.S., Blevins, M., Cardoso, A., Moon, T.D. and Sidat, M. (2012). Acceptability of cervical cancer screening in rural Mozambique. *Health Education Research*, 27(3), pp.544–551. https://doi.org/10.1093/her/cys008.

Avian, A., Clemente, N., Mauro, E., Isidoro, E., Di Napoli, M., Dudine, S., Del Fabro, A., Morini, S., Perin, T., Giudici, F., Cammisuli, T., Foschi, N., Mocenigo, M., Montrone, M., Modena, C., Polenghi, M., Puzzi, L., Tomaic, V., Valenti, G. and Sola, R. (2022). Clinical validation of full HR-HPV genotyping HPV Selfy assay according to the international guidelines for HPV test requirements for cervical cancer screening on clinician-collected and self-collected samples. *Journal of Translational Medicine*, [online] 20, p.231. https://doi.org/10.1186/s12967-022-03383-x.

Baddou, R., Yacouti, A., Guennouni, M., got, A.E., Benider, A. and Mouallif, M. (2024). Study of Knowledge, Attitudes, Acceptability and Preference of Home-based HPV Self-Sampling Among a Population of Moroccan Women. *Journal of Cancer Education*. https://doi.org/10.1007/s13187-024-02503-z.

Bakiewicz, A., Rasch, V., Mwaiselage, J., & Linde, D. S. (2020). “The best thing is that you are doing it for yourself”–perspectives on acceptability and feasibility of HPV self-sampling among cervical cancer screening clients in Tanzania: a qualitative pilot study. *BMC Women's Health*, *20*(1), 65. doi: https://doi.org/10.1186/s12905-020-00917-7.

Bancroft, E., Saya, S., Brown, E., Thomas, S., Taylor, N., Rothwell, J., Pope, J., Chamberlain, A., Page, E., Benafif, S., Hanson, H., Dias, A., Christos Mikropoulos, Izatt, L., Side, L., Walker, L., Donaldson, A., Cook, J., Barwell, J. and Wiles, V. (2019). Psychosocial effects of whole-body MRI screening in adult high-risk pathogenic *TP53* mutation carriers: a case-controlled study (SIGNIFY). *Journal of Medical Genetics*, 57(4), pp.226–236. https://doi.org/10.1136/jmedgenet-2019-106407.

Banerjee, D., Mandal, R., Mandal, A., Ghosh, I., Mittal, S., Muwonge, R., Lucas, E. and Basu, P. (2020). A Prospective Randomized Trial to Compare Safety, Acceptability and Efficacy of Thermal Ablation and Cryotherapy in a Screen and Treat Setting. *Asian Pacific Journal of Cancer Prevention*, 21(5), pp.1391–1398. https://doi.org/10.31557/apjcp.2020.21.5.1391.

Bansil, P., Wittet, S., Lim, J.L., Winkler, J.L., Paul, P. and Jeronimo, J. (2014). Acceptability of self-collection sampling for HPV-DNA testing in low-resource settings: a mixed methods approach. *BMC Public Health*, 14(1). https://doi.org/10.1186/1471-2458-14-596.

Barbee, L., Kobetz, E., Menard, J., Cook, N., Blanco, J., Barton, B., Auguste, P. and McKenzie, N. (2009). Assessing the acceptability of self-sampling for HPV among Haitian immigrant women: CBPR in action. *Cancer Causes & Control*, 21(3), pp.421–431. https://doi.org/10.1007/s10552-009-9474-0.

Behnke, A.-L., Krings, A., Wormenor, C.M., Dunyo, P., Kaufmann, A.M. and Amuah, J.E. (2020). Female health-care providers’ advocacy of self-sampling after participating in a workplace program for cervical cancer screening in Ghana: a mixed-methods study. *Global Health Action*, 13(1), p.1838240. https://doi.org/10.1080/16549716.2020.1838240.

Belete, N., Tsige, Y. and Mellie, H. (2015). Willingness and acceptability of cervical cancer screening among women living with HIV/AIDS in Addis Ababa, Ethiopia: a cross sectional study. *Gynecologic Oncology Research and Practice*, [online] 2(1). https://doi.org/10.1186/s40661-015-0012-3.

Berner, A., Hassel, S.B., Tebeu, P.-M., Untiet, S., Kengne-Fosso, G., Navarria, I., Boulvain, M., Vassilakos, P. and Petignat, P. (2013). Human Papillomavirus Self-Sampling in Cameroon: Women’s Uncertainties Over the Reliability of the Method Are Barriers to Acceptance. *Journal of Lower Genital Tract Disease*, [online] 17(3), pp.235–241. https://doi.org/10.1097/LGT.0b013e31826b7b51.

Bertucci, M., Bonnet, E., Satger, L., Kreiche, A., Chappert, J. L., Loy-Morel, S., … Boulle, N. (2020). Acceptability of vaginal self-sampling with high-risk human papillomavirus testing for cervical cancer screening: a French questionnaire-based study. *Women & Health*, *61*(1), 83–94. https://doi.org/10.1080/03630242.2020.1831683

Biederman, E., Champion, V. and Zimet, G. (2020). A conjoint analysis study on self-sampling for human papillomavirus (HPV) testing characteristics among black women in Indiana. *BMC Women’s Health*, 20(1). https://doi.org/10.1186/s12905-020-00921-x.

Billette de Villemeur, A., Exbrayat, C., Garnier, A., Ancelle-Park, R., Ferley, J.-P. and Jestin, C. (2007). Evaluation of a combined screening programme for breast, cervical and colorectal cancers in France. *European Journal of Cancer Prevention*, 16(1), pp.26–35. https://doi.org/10.1097/01.cej.0000228399.30235.bc.

Bishop, E., Katz, M.L. and Reiter, P.L. (2019). Acceptability of Human Papillomavirus Self-Sampling Among a National Sample of Women in the United States. *BioResearch Open Access*, 8(1), pp.65–73. https://doi.org/10.1089/biores.2018.0040.

Blankenship, S.A., Debnath, P., Szlachta-McGinn, A., Maguire, K., Garcia, J.J., Aserlind, A., Lipshultz, E.R. and Potter, J. (2016). Knowledge and Acceptability of Anal Cytology Screening Among Women. *Journal of Lower Genital Tract Disease*, 20(1), pp.90–96. https://doi.org/10.1097/lgt.0000000000000151.

Blouin-Bougie, J., Amara, N. and Simard, J. (2021). Toward a Population-Based Breast Cancer Risk Stratification Approach? The Needs and Concerns of Healthcare Providers. *Journal of Personalized Medicine*, 11(6), p.540. https://doi.org/10.3390/jpm11060540.

Bobridge, A., Price, K., Gill, T.K. and Taylor, A.W. (2017). Influencing Cancer Screening Participation Rates—Providing a Combined Cancer Screening Program (a ‘One Stop’ Shop) Could Be a Potential Answer. *Frontiers in Oncology*, 7. https://doi.org/10.3389/fonc.2017.00308.

Bong, G. and McCool, J. (2011). Chinese peoples’ perceptions of colorectal cancer screening: a New Zealand perspective. *PubMed*, 124(1331), pp.29–38.

Bosgraaf, R.P., Verhoef, V.M.J., Massuger, L.F.A.G., Siebers, A.G., Bulten, J., de Kuyper-de Ridder, G.M., Meijer, C.J.M., Snijders, P.J.F., Heideman, D.A.M., IntHout, J., van Kemenade, F.J., Melchers, W.J.G. and Bekkers, R.L.M. (2014). Comparative performance of novel self-sampling methods in detecting high-risk human papillomavirus in 30,130 women not attending cervical screening. *International Journal of Cancer*, p.n/a-n/a. https://doi.org/10.1002/ijc.29026.

Bowen, D.J., Weiner, D., Samos, M. and Canales, M.K. (2014). Exploration of New England Native American Women’s Views on Human Papillomavirus (HPV), Testing, and Vaccination. *Journal of Racial and Ethnic Health Disparities*, 1(1), pp.45–51. https://doi.org/10.1007/s40615-014-0009-3.

Brandt, T., Wubneh, S.B., Handebo, S., Debalkie, G., Ayanaw, Y., Alemu, K., Jede, F., von Knebel Doeberitz, M. and Bussmann, H. (2019). Genital self-sampling for HPV-based cervical cancer screening: a qualitative study of preferences and barriers in rural Ethiopia. *BMC Public Health*, 19(1). https://doi.org/10.1186/s12889-019-7354-4.

Brett, T. (1998). An analysis of digital rectal examination and serum-prostate-specific antigen in the early detection of prostate cancer in general practice. *Family Practice*, 15(6), pp.529–533. https://doi.org/10.1093/fampra/15.6.529.

Brewer, N., Bartholomew, K., Grant, J., Maxwell, A., McPherson, G., Wihongi, H., Bromhead, C., Scott, N., Crengle, S., Foliaki, S., Cunningham, C., Douwes, J. and Potter, J.D. (2021). Acceptability of human papillomavirus (HPV) self-sampling among never- and under-screened Indigenous and other minority women: a randomised three-arm community trial in Aotearoa New Zealand. *The Lancet Regional Health - Western Pacific*, 16, p.100265. https://doi.org/10.1016/j.lanwpc.2021.100265.

Brewer, N., Foliaki, S., Bromhead, C., Viliamu-Amusia, I., Pelefoti-Gibson, L., Jones, T., Pearce, N., Potter, J. D., & Douwes, J. (2019). Acceptability of human papillomavirus self-sampling for cervical-cancer screening in under-screened Māori and Pasifika women: a pilot study. *The New Zealand medical journal*, *132*(1497), 21–31.

Brewster, S.F., Kemple, T., Maciver, A.G., Astley, J.P. And GINGELL, J.C. (1994). The Bristol prostate cancer pilot screening study — a 3‐year follow‐up. *British Journal of Urology*, 74(5), pp.556–558. https://doi.org/10.1111/j.1464-410x.1994.tb09183.x.

Brewster, W.R., Hubbell, F.A., Largent, J., Ziogas, A., Lin, F., Howe, S., Ganiats, T.G., Hoda Anton-Culver and Manetta, A. (2005). Feasibility of Management of High-Grade Cervical Lesions in a Single Visit. *JAMA*, 294(17), pp.2182–2182. https://doi.org/10.1001/jama.294.17.2182.

Bromhead, C., Wihongi, H., Sherman, S.M., Crengle, S., Grant, J., Martin, G., Maxwell, A., McPherson, G., Puloka, A., Reid, S., Scott, N. and Bartholomew, K. (2021). Human Papillomavirus (HPV) Self-Sampling among Never-and Under-Screened Indigenous Māori, Pacific and Asian Women in Aotearoa New Zealand: A Feasibility Study. *International Journal of Environmental Research and Public Health*, [online] 18(19), p.10050. https://doi.org/10.3390/ijerph181910050.

Broquet, C., Triboullier, D., Untiet, S., Schafer, S., Petignat, P. and Vassilakos, P. (2015). Acceptability of self-collected vaginal samples for HPV testing in an urban and rural population of Madagascar. *African Health Sciences*, 15(3), p.755. https://doi.org/10.4314/ahs.v15i3.8.

Brotzman, L.E., Zikmund-Fisher, B.J., Kerr, E.A., Kabeto, M. and Kullgren, J.T. (2024). Acceptability of Guidelines to Stop Colon Cancer Screening by Estimated Life Expectancy. *JAMA Network Open*, [online] 7(12), p.e2447802. https://doi.org/10.1001/jamanetworkopen.2024.47802.

Brown, L.R., Sullivan, F., Treweek, S., Haddow, A., Mountain, R., Selby, C. and Beusekom, M. van (2022). Increasing uptake to a lung cancer screening programme: building with communities through co-design. *BMC Public Health*, 22(1). https://doi.org/10.1186/s12889-022-12998-0.

Buadi, L.O., Odame, R., Samba, A., Kareem, M., Boateng, A.A., Diogo, C.A., Morrision, R.T., Kwakye, G.K. and Smith-Togobo, C. (2024). Integration and acceptability of cervical cancer screening as part of postnatal care among women attending postnatal clinics in the Accra Metropolis, Greater Accra Region, Ghana. *PLOS Global Public Health*, 4(12), pp.e0004070–e0004070. https://doi.org/10.1371/journal.pgph.0004070.

Bulliard, J.-L., De Landtsheer, J.-P. . and Levi, F. (2003). Results from the Swiss mammography screening pilot programme. *European Journal of Cancer*. https://doi.org/10.1016/s0959-8049(03)00238-7.

Burton-Jeangros, C., Fargnoli, V. and Petignat, P. (2015). To what extent will women accept HPV self-sampling for cervical cancer screening? A qualitative study conducted in Switzerland. *International Journal of Women’s Health*, p.883. https://doi.org/10.2147/ijwh.s90772.

Busingye, P., Nakimuli, A., Nabunya, E. and Mutyaba, T. (2012). Acceptability of cervical cancer screening via visual inspection with acetic acid or Lugol’s iodine at Mulago Hospital, Uganda. *International Journal of Gynecology & Obstetrics*, 119(3), pp.262–265. https://doi.org/10.1016/j.ijgo.2012.06.015.

Butame, S.A., Lawler, S., Hicks, J.T., Wilkerson, J.M., Hwang, L.-Y., Baraniuk, S., Ross, M.W., Chiao, E.Y. and Nyitray, A.G. (2017). A qualitative investigation among men who have sex with men on the acceptability of performing a self- or partner anal exam to screen for anal cancer. *Cancer Causes & Control*, 28(10), pp.1157–1166. https://doi.org/10.1007/s10552-017-0935-6.

Carcioppolo, N., Christy, K.R., Jensen, J.D., King, A.J., Goonewardene, J. and Raftery, D. (2018). Biomarker profiling for breast cancer detection: translational research to determine acceptance of a novel breast cancer screening technique. *Health Systems*, 8(1), pp.44–51. https://doi.org/10.1080/20476965.2017.1414740.

Carruthers, J., Wilson, J.M., Chamberlain, J., Husain, O.A., Patey, D.G., Richards, N.D., A Pennicott, Rogers, P., Catling, R., Meade, T.W., Saunders, J. and McEwan, P.J. (1975). Acceptability of the cytopipette in screening for cervical cancer. *Journal of epidemiology and community health*, 29(4), pp.239–248. https://doi.org/10.1136/jech.29.4.239.

Catarino, R., Vassilakos, P., Stadali-Ullrich, H., Royannez-Drevard, I., Guillot, C. and Petignat, P. (2015). Feasibility of At-Home Self-Sampling for HPV Testing as an Appropriate Screening Strategy for Nonparticipants in Switzerland. *Journal of Lower Genital Tract Disease*, 19(1), pp.27–34. https://doi.org/10.1097/lgt.0000000000000051.

Cavers, D., Nelson, M., Rostron, J., Robb, K.A., Brown, L.R., Campbell, C., Akram, A.R., Dickie, G., Mackean, M., van Beek, E.J.R., Sullivan, F., Steele, R.J., Neilson, A.R. and Weller, D. (2022). Optimizing the implementation of lung cancer screening in Scotland: Focus group participant perspectives in the LUNGSCOT study. *Health Expectations*. https://doi.org/10.1111/hex.13632.

Chatzistamatiou, K., Chatzaki, Ε., Constantinidis, Τ., Nena, E., Tsertanidou, A. and Agorastos, T. (2017). Self-collected cervicovaginal sampling for site-of-care primary HPV-based cervical cancer screening: a pilot study in a rural underserved Greek population. *Journal of Obstetrics and Gynaecology*, 37(8), pp.1059–1064. https://doi.org/10.1080/01443615.2017.1323197.

Chatzistamatiou, K., Vrekoussis, T., Tsertanidou, A., Moysiadis, T., Mouchtaropoulou, E., Pasentsis, K., Kitsou, A., Moschaki, V., Ntoula, M., Zempili, P., Halatsi, D., Truva, T., Piha, V., Agelena, G., Daponte, A., Vanakara, P., Paschopoulos, M., Stefos, T., Lymberis, V. and Kontomanolis, E.N. (2020). Acceptability of Self-Sampling for Human Papillomavirus-Based Cervical Cancer Screening. *Journal of Women’s Health*, 29(11), pp.1447–1456. https://doi.org/10.1089/jwh.2019.8258.

Chaw, L., Lee, S.H.F., Ja’afar, N.I.H., Lim, E. and Sharbawi, R. (2022). Reasons for non-attendance to cervical cancer screening and acceptability of HPV self-sampling among Bruneian women: A cross-sectional study. *PLOS ONE*, 17(3), p.e0262213. https://doi.org/10.1371/journal.pone.0262213.

Chino, Y., Onuma, T., Ito, T., Shinagawa, A., Kurokawa, T., Makoto Orisaka and Yoshida, Y. (2024). Human Papillomavirus Self-Sampling for Unscreened Women Aged 24 Years During the COVID-19 Pandemic. *Healthcare*, 12(21), pp.2160–2160. https://doi.org/10.3390/healthcare12212160.

Cho, A., Chaudhry, A., Minsky-Primus, L., Tso, A., Perez-Perez, G., Diehl, D., Marcus, S.G. and Gany, F.M. (2006). Acceptance of Repeat Esophagogastroduodenoscopy to Detect Gastric Cancer in a Chinese Immigrant Cohort. *Journal of Clinical Gastroenterology*, 40(7), pp.606–611. https://doi.org/10.1097/00004836-200608000-00009.

Cicconi, P., Wells, C., McCarthy, B., Wareing, S., Andersson, M.I., Fox, J., Lwanga, J., Pal, N., Burns, F., Woodward, C., Malek, R., Sabin, C.A. and Dorrell, L. (2022). Re‐valuation of annual cytology using HPV self‐sampling to upgrade prevention (REACH UP): A feasibility study in women living with HIV in the UK. *HIV Medicine*, 23(4), pp.390–396. https://doi.org/10.1111/hiv.13257.

Coady, M.S., Benson, E.A. and Hartley, M.N. (1993). Provision and acceptability of day case breast biopsy: an audit of current practice. *Annals of the Royal College of Surgeons of England*, *75*(4), 281.

Cockburn, J., Thomas, R.J.S., McLaughlin, S.J. and Reading, D. (1995). Acceptance of Screening for Colorectal Cancer by Flexible Sigmoidoscopy. *Journal of Medical Screening*, 2(2), pp.79–83. https://doi.org/10.1177/096914139500200205.

Colling, A.M., Creagh, N.S., Neha Gogia, Wyatt, K., Zammit, C., Julia and Nightingale, C.E. (2024). The acceptability of, and informational needs related to, self‐collection cervical screening among women of Indian descent living in Victoria, Australia: A qualitative study. *Health Expectations*, 27(1). https://doi.org/10.1111/hex.13961.

Condon, A., Graff, L., Elliot, L. and Ilnyckyj, A. (2008). Acceptance of Colonoscopy Requires more than Test Tolerance. *Canadian Journal of Gastroenterology*, 22(1), pp.41–47. https://doi.org/10.1155/2008/107467.

Cooper, C.P., Saraiya, M. and Sawaya, G.F. (2015). Acceptable and Preferred Cervical Cancer Screening Intervals Among U.S. Women. *American Journal of Preventive Medicine*, [online] 49(6), pp.e99–e107. https://doi.org/10.1016/j.amepre.2015.04.025.

Creagh, N.S., Zammit, C., Brotherton, J.M., Saville, M., McDermott, T., Nightingale, C. and Kelaher, M. (2021). Self‐collection cervical screening in the renewed National Cervical Screening Program: a qualitative study. *Medical Journal of Australia*, 215(8). https://doi.org/10.5694/mja2.51137.

Crespo, B.V., Neira, V.A., Ortíz S, J., Maldonado-Rengel, R., López, D., Gómez, A., Vicuña, M.J., Mejía, J., Benoy, I., Carreño, T.P. and Verhoeven, V. (2022). Evaluation of Urine and Vaginal Self-Sampling versus Clinician-Based Sampling for Cervical Cancer Screening: A Field Comparison of the Acceptability of Three Sampling Tests in a Rural Community of Cuenca, Ecuador. *Healthcare*, 10(9), p.1614. https://doi.org/10.3390/healthcare10091614.

Crofts, V., Flahault, E., Pierre Marie Tebeu, Untiet, S., Gisele Kengne Fosso, Boulvain, M., Vassilakos, P. and Petignat, P. (2015). Education efforts may contribute to wider acceptance of human papillomavirus self-sampling. pp.149–149. https://doi.org/10.2147/ijwh.s56307.

Cunningham, M.S., Skrastins, E., Fitzpatrick, R., Jindal, P., Oneko, O., Yeates, K., Booth, C.M., Carpenter, J. and Aronson, K.J. (2015). Cervical cancer screening and HPV vaccine acceptability among rural and urban women in Kilimanjaro Region, Tanzania. *BMJ Open*, 5(3), pp.e005828–e005828. https://doi.org/10.1136/bmjopen-2014-005828.

Dannecker, C., Siebert, U., Thaler, C.J., Kiermeir, D., Hepp, H. and Hillemanns, P. (2004). Primary cervical cancer screening by self-sampling of human papillomavirus DNA in internal medicine outpatient clinics. *Annals of Oncology*, 15(6), pp.863–869. https://doi.org/10.1093/annonc/mdh240.

Davies, J.C., Sargent, A., Pinggera, E., Carter, S., Gilham, C., Sasieni, P. and Crosbie, E.J. (2024). Urine high‐risk human papillomavirus testing as an alternative to routine cervical screening: A comparative diagnostic accuracy study of two urine collection devices using a randomised study design trial. *BJOG An International Journal of Obstetrics & Gynaecology*. https://doi.org/10.1111/1471-0528.17831.

Davis, M.M., Coury, J., Larson, J.H., Gunn, R., Towey, E.G., Ketelhut, A., Patzel, M., Ramsey, K. and Coronado, G.D. (2022). Improving colorectal cancer screening in rural primary care: Preliminary effectiveness and implementation of a collaborative mailed fecal immunochemical test pilot. *The Journal of Rural Health*. https://doi.org/10.1111/jrh.12685.

Degeling, C., Barratt, A., Aranda, S., Bell, R., Doust, J., Houssami, N., Hersch, J., Sakowsky, R., Entwistle, V. and Carter, S.M. (2018). Should women aged 70–74 be invited to participate in screening mammography? A report on two Australian community juries. *BMJ Open*, 8(6), p.e021174. https://doi.org/10.1136/bmjopen-2017-021174.

Dennison, R.A., Boscott, R.A., Thomas, R., Griffin, S.J., Harrison, H., John, S.D., Moorthie, S.A., Morris, S., Rossi, S.H., Stewart, G.D., Thomas, C.V. and Usher‐Smith, J.A. (2022). A community jury study exploring the public acceptability of using risk stratification to determine eligibility for cancer screening. *Health Expectations*, 25(4), pp.1789–1806. https://doi.org/10.1111/hex.13522.

Devotta, K., Vahabi, M., Prakash, V. and Lofters, A. (2023a). Reach and effectiveness of an HPV self-sampling intervention for cervical screening amongst under- or never-screened women in Toronto, Ontario Canada. *BMC Women’s Health*, 23(1). https://doi.org/10.1186/s12905-023-02174-w.

Devotta, K., Vahabi, M., Prakash, V. and Lofters, A.K. (2023b). Implementation of a Cervical Cancer Screening Intervention for Under- or Never-Screened Women in Ontario, Canada: Understanding the Acceptability of HPV Self-Sampling. *Current Oncology (Toronto, Ont.)*, [online] 30(7), pp.6786–6804. https://doi.org/10.3390/curroncol30070497.

Di Gennaro, G., Licata, F., Trovato, A. and Bianco, A. (2022). Does self-sampling for human papilloma virus testing have the potential to increase cervical cancer screening? An updated meta-analysis of observational studies and randomized clinical trials. *Frontiers in Public Health*, 10. https://doi.org/10.3389/fpubh.2022.1003461.

Djuric, O., Deandrea, S., Mantellini, P., Sardanelli, F., Venturelli, F., Montemezzi, S., Vecchio, R., Bucchi, L., Senore, C., Giordano, L., Paci, E., Bonifacino, A., Calabrese, M., Caumo, F., Degrassi, F., Sassoli, P., Battisti, F., Zappa, M., Pierpaolo Pattacini and Cinzia Campari (2024). Organizational impact of systemic implementation of digital breast tomosynthesis as a primary test for breast cancer screening in Italy. *La radiologia medica*, 129(8), pp.1156–1172. https://doi.org/10.1007/s11547-024-01849-0.

Djuric, O., Giorgi Rossi, P., Ivanciu, E.C., Cardellicchio, S., Cresci, C., Carozzi, L., Pistelli, F., Bessi, V., Gai, P., Galli, V., Lavacchini, G., Bricci, C., Gorini, G., Bosi, S. and Paci, E. (2023). Motivation, acceptability and attitudes toward lung cancer screening among persons who attend a tobacco cessation program: A multicenter survey in Italy. *Preventive Medicine Reports*, [online] 35, p.102272. https://doi.org/10.1016/j.pmedr.2023.102272.

Dodd, R.H., Sharman, A.R., Marshall, H.M., Mei Ling Yap, Stone, E., Rhee, J.J., McCullough, S. and Rankin, N.M. (2023). ‘What do I think about implementing lung cancer screening? It all depends on how.’ Acceptability and feasibility of lung cancer screening in Australia: The view of key stakeholders about health system factors. *plos one*, 18(4), pp.e0283939–e0283939. https://doi.org/10.1371/journal.pone.0283939.

Dodd, R.H., Sharman, A.R., Yap, M.L., Stone, E., Marshall, H., Rhee, J., McCullough, S. and Rankin, N.M. (2024). ‘We need to work towards it, whatever it takes.’—participation factors in the acceptability and feasibility of lung cancer screening in Australia: the perspectives of key stakeholders. *Translational Lung Cancer Research*, 13(2), pp.240–255. https://doi.org/10.21037/tlcr-23-623.

Dotson, M.E., Cuenca, E., Krieger, M., Ramanujam, N. and Garcia, P. (2021). Acceptability and Feasibility of Callascope Self-Imaging of the Cervix among Women in Ventanilla, Peru: A Mixed Methods Pilot Study. *PubMed*, 5(3).

Downham, L., Rol, M.L., Forestier, M., Romero, P., Mendoza, L., Mongelós, P., María Alejandra Picconi, María Celeste Colucci, Valeria Mariel Padin, Ana Paula Flores, Zúñiga, M., Ferrera, A., Cabrera, Y., Marcela Farfan Crispín, Arianis Tatiana Ramirez, Londiwe Cele, Halimatou Diop‐Ndiaye, Dianke Samaté, Manga, P. and Fadimatou Bintou Thiam (2024). Field experience with the 8‐HPV‐type oncoprotein test for cervical cancer screening among HPV‐positive women living with and without HIV in LMICs. *International journal of cancer*. https://doi.org/10.1002/ijc.34953.

Dunlop, K., Rankin, N.M., Smit, A.K., Salgado, Z., Newson, A.J., Keogh, L. and Cust, A.E. (2021). Acceptability of risk‐stratified population screening across cancer types: Qualitative interviews with the Australian public. *Health Expectations*, 24(4), pp.1326–1336. https://doi.org/10.1111/hex.13267.

Dunlop, K.L., Smit, A.K., Keogh, L.A., Newson, A.J., Rankin, N.M. and Cust, A.E. (2024). Acceptability of risk-tailored cancer screening among Australian GPs: a qualitative study. *British Journal of General Practice*, [online] 74(740), pp.e156–e164. https://doi.org/10.3399/BJGP.2023.0117.

Dunlop, K.L.A., Keogh, L.A., Smith, A.L., Aranda, S., Aitken, J., Watts, C.G., Smit, A.K., Janda, M., Mann, G.J., Cust, A.E. and Rankin, N.M. (2023). Acceptability and appropriateness of a risk-tailored organised melanoma screening program: Qualitative interviews with key informants. *PloS one*, [online] 18(12), pp.e0287591–e0287591. https://doi.org/10.1371/journal.pone.0287591.

Dyer, C.E.F., Jin, F., Roberts, J.M., Poynten, I.M., Farnsworth, A., McNally, L.P., Cunningham, P.H., Grulich, A.E. and Hillman, R.J. (2025). Self- versus clinician-collected swabs in anal cancer screening: A clinical trial. *PloS one*, [online] 20(1), p.e0312781. https://doi.org/10.1371/journal.pone.0312781.

Dzuba, I.G., Díaz, E.Y., Allen, B., Leonard, Y.F., Lazcano Ponce, E.C., Shah, K.V., Bishai, D., Lorincz, A., Ferris, D., Turnbull, B., Hernández Avila, M. and Salmerón, J. (2002). The acceptability of self-collected samples for HPV testing vs. the pap test as alternatives in cervical cancer screening. *Journal of Women’s Health & Gender-Based Medicine*, [online] 11(3), pp.265–275. https://doi.org/10.1089/152460902753668466.

Eduardo, C., Falang, B.M., Gómez-Laguna, L., Gutiérrez, G.G., Miguel, J., Uribe, M., Cruz, O. and Sveinung Wergeland Sørbye (2024). Enhancing Cervical Cancer Screening with 7-Type HPV mRNA E6/E7 Testing on Self-Collected Samples: Multicentric Insights from Mexico. *Cancers*, 16(13), pp.2485–2485. https://doi.org/10.3390/cancers16132485.

Ellis, R.J.B., Wilson, S., Holder, R.L. and McManus, R.J. (2007). Different faecal sampling methods alter the acceptability of faecal occult blood testing: A cross sectional community survey. *European Journal of Cancer*, 43(9), pp.1437–1444. https://doi.org/10.1016/j.ejca.2007.03.019.

Elmasry, K., Davies, A.J., Evans, D.G., Seif, M.N. and Reynolds, K. (2009). Strategies for endometrial screening in the Lynch syndrome population: a patient acceptability study. *Familial Cancer*, [online] 8(4), pp.431–439. https://doi.org/10.1007/s10689-009-9259-3.

Eperon, I., Vassilakos, P., Navarria, I., Menoud, P.-A., Gauthier, A., Pache, J.-C., Boulvain, M., Untiet, S. and Petignat, P. (2013). Randomized comparison of vaginal self-sampling by standard vs. dry swabs for Human papillomavirus testing. *BMC Cancer*, 13(1). https://doi.org/10.1186/1471-2407-13-353.

Ertik, F.C., Kampers, J., Hülse, F., Stolte, C., Böhmer, G., Hillemanns, P. and Jentschke, M. (2021). CoCoss-Trial: Concurrent Comparison of Self-Sampling Devices for HPV-Detection. *International Journal of Environmental Research and Public Health*, 18(19), p.10388. https://doi.org/10.3390/ijerph181910388.

Esber, A., McRee, A.-L., Norris Turner, A., Phuka, J. and Norris, A. (2016). Factors influencing Malawian women’s willingness to self-collect samples for human papillomavirus testing. *Journal of Family Planning and Reproductive Health Care*, 43(2), pp.135–141. https://doi.org/10.1136/jfprhc-2015-101305.

Esber, A. and Norris, A. (2018). Feasibility, validity and acceptability of self-collected samples for human papillomavirus (HPV) testing in rural Malawi. *Malawi Medical Journal*, 30(2), p.61. https://doi.org/10.4314/mmj.v30i2.2.

Eshete, M., Abdulwuhab Atta, M. and Yeshita, H.Y. (2020). Cervical Cancer Screening Acceptance among Women in Dabat District, Northwest Ethiopia, 2017: An Institution-Based Cross-Sectional Study. *Obstetrics and Gynecology International*, 2020, pp.1–7. https://doi.org/10.1155/2020/2805936.

Esquivel-Sada, D., Lévesque, E., Hagan, J., Knoppers, B. and Simard, J. (2019). Envisioning Implementation of a Personalized Approach in Breast Cancer Screening Programs: Stakeholder Perspectives. *Healthcare Policy | Politiques de Santé*, 15(2), pp.39–54. https://doi.org/10.12927/hcpol.2019.26072.

Ezechi, O.C., Gab-Okafor, C.V., Ostergren, P.O. and Odberg Pettersson, K. (2013). Willingness and acceptability of cervical cancer screening among HIV positive Nigerian women. *BMC Public Health*, 13(1). <https://doi.org/10.1186/1471-2458-13-46>.

Faivre, J., Dancourt, V., Lejeune, C., Tazi, M. A., Lamour, J., Gerard, D., Dassonville, F., & Bonithon-Kopp, C. (2004). Reduction in colorectal cancer mortality by fecal occult blood screening in a French controlled study. *Gastroenterology*, *126*(7), 1674–1680. <https://doi.org/10.1053/j.gastro.2004.02.018>

Fall, N.S., Tamalet, C., Diagne, N., Fenollar, F., Raoult, D., Sokhna, C. and Lagier, J.-C. (2019). Feasibility, Acceptability, and Accuracy of Vaginal Self-Sampling for Screening Human Papillomavirus Types in Women from Rural Areas in Senegal. *The American Journal of Tropical Medicine and Hygiene*, 100(6), pp.1552–1555. https://doi.org/10.4269/ajtmh.19-0045.

Fallala, M.S. and Mash, R. (2015). Cervical cancer screening: Safety, acceptability, and feasibility of a single-visit approach in Bulawayo, Zimbabwe. *African Journal of Primary Health Care & Family Medicine*, 7(1). https://doi.org/10.4102/phcfm.v7i1.742.

Filade, T.E., Dareng, E.O., Olawande, T., Fagbohun, T.A., Adebayo, A.O. and Adebamowo, C.A. (2017). Attitude to Human Papillomavirus Deoxyribonucleic Acid-Based Cervical Cancer Screening in Antenatal Care in Nigeria: A Qualitative Study. *Frontiers in public health*, [online] 5, p.226. https://doi.org/10.3389/fpubh.2017.00226.

Flores, Y., Bishai, D., Lazcano, E., Shah, K., Lörincz, A., Hernández, M., Salmerón, J., Ferris, D., Hernández, P., Sherman, M.E., Ronnett, B.M., Carmona, E., Antúnez, A., Manzanares, H., Uribe, M., Pérez-Cuevas, R., Leyva, A. and Yunes, E. (2003). Improving cervical cancer screening in Mexico: results from the Morelos HPV Study. *Salud Pública de México*, 45, pp.388–398. https://doi.org/10.1590/s0036-36342003000900013.

Forbes, G.M., Mendelson, R.M., Edwards, J.T., Foster, N.M., Pawlik, J.Z., Bampton, P.A., Voyvodic, F., Upton, J., Macrae, F.A., Stella, D., Viney, B., Pizzey, C.J., Fritschi, L. and Heyworth, J. (2006). A comparison of colorectal neoplasia screening tests: a multicentre community‐based study of the impact of consumer choice. *Medical Journal of Australia*, 184(11), pp.546–550. https://doi.org/10.5694/j.1326-5377.2006.tb00377.x.

Freeman, M., Waller, J., Sasieni, P., Lim, A.W. and Marlow, L.A. (2018). Acceptability of non-speculum clinician sampling for cervical screening in older women: A qualitative study. *Journal of Medical Screening*, 25(4), pp.205–210. https://doi.org/10.1177/0969141318756452.

French, D.P., Woof, V.G., Ruane, H., Evans, D.G., Ulph, F. and Donnelly, L.S. (2022). The feasibility of implementing risk stratification into a national breast cancer screening programme: a focus group study investigating the perspectives of healthcare personnel responsible for delivery. *BMC Women’s Health*, 22(1). https://doi.org/10.1186/s12905-022-01730-0.

Fujita, M., Nagashima, K., Shimazu, M., Suzuki, M., Tauchi, I., Sakuma, M., Yamamoto, S., Hanaoka, H., Shozu, M., Tsuruoka, N., Kasai, T. and Hata, A. (2023). Acceptability of self-sampling human papillomavirus test for cervical cancer screening in Japan: A questionnaire survey in the ACCESS trial. *PloS One*, [online] 18(6), p.e0286909. https://doi.org/10.1371/journal.pone.0286909.

Gaba, F., Blyuss, O., Liu, X., Goyal, S., Lahoti, N., Chandrasekaran, D., Kurzer, M., Kalsi, J., Sanderson, S., Lanceley, A., Ahmed, M., Side, L., Gentry-Maharaj, A., Wallis, Y., Wallace, A., Waller, J., Luccarini, C., Yang, X., Dennis, J. and Dunning, A. (2020). Population Study of Ovarian Cancer Risk Prediction for Targeted Screening and Prevention. *Cancers*, 12(5), p.1241. https://doi.org/10.3390/cancers12051241.

Gaffikin, L., Blumenthal, P.D., Emerson, M. and Limpaphayon, K. (2003). Safety, acceptability, and feasibility of a single-visit approach to cervical-cancer prevention in rural Thailand: a demonstration project. *The Lancet*, 361(9360), pp.814–820. <https://doi.org/10.1016/s0140-6736(03)12707-9>.

Gao, Y., Xin, L., Feng, Y. D., Yao, B., Lin, H., Sun, C., An, W., Li, Z. S., Shi, R. H., & Wang, L. W. (2021). Feasibility and Accuracy of Artificial Intelligence-Assisted Sponge Cytology for Community-Based Esophageal Squamous Cell Carcinoma Screening in China. *The American journal of gastroenterology*, *116*(11), 2207–2215. <https://doi.org/10.14309/ajg.0000000000001499>

Garcia, P.D., Snoeckx, A., Van, J.P. and Hal, G.V. (2022). A Cross-Sectional Study on the Acceptability of Implementing a Lung Cancer Screening Program in Belgium. *Cancers*, 15(1), pp.278–278. https://doi.org/10.3390/cancers15010278.

Gentry‐Maharaj, A., Sharma, A., Burnell, M., Ryan, A., Amso, N.N., Seif, M.W., Turner, G., Brunell, C., Fletcher, G., Rangar, R., Fallowfield, L., Campbell, S., Jacobs, I. and Menon, U. (2012). Acceptance of transvaginal sonography by postmenopausal women participating in the United Kingdom Collaborative Trial of Ovarian Cancer Screening. *Ultrasound in Obstetrics & Gynecology*, 41(1), pp.73–79. https://doi.org/10.1002/uog.12262.

Ghanouni, A., Sanderson, S.C., Pashayan, N., Renzi, C., von Wagner, C. and Waller, J. (2019a). Attitudes towards risk-stratified breast cancer screening among women in England: A cross-sectional survey. *Journal of Medical Screening*, 27(3), pp.138–145. https://doi.org/10.1177/0969141319883662.

Ghanouni, A., Waller, J., Stoffel, S.T., Vlaev, I. and von Wagner, C. (2019b). Acceptability of risk-stratified breast screening: Effect of the order of presenting risk and benefit information. *Journal of Medical Screening*, 27(1), pp.52–56. https://doi.org/10.1177/0969141319877669.

Gillis, J.L., Grennan, T., Grewal, R., Ogilvie, G., Gaspar, M., Grace, D., Lofters, A., Raboud, J.M., Saarela, O., MacPherson, P., Rosenes, R., Salit, I.E., Burchell, A.N., Kroch, A., Burchell, A., Rueda, S., Arbess, G., Cohen, J., Cooper, C. and Lavoie, E. (2022). Influence of previous experience with and beliefs regarding anal cancer screening on willingness to be screened among men living with HIV. *BMC Public Health*, 22(1). https://doi.org/10.1186/s12889-022-14471-4.

Glass-Riveros, E., Baumann, K., Craemer, K., Geller, S., Frenier, M.N., McDonald, J. and Holt, H.K. (2024). The Acceptability and Feasibility of Self-Collected HPV Testing for Cervical Cancer Screening Among Black and Latinx Women in Chicago: Perspectives from the Community. *Women s Health Reports*, 5(1), pp.735–743. https://doi.org/10.1089/whr.2024.0102.

Godtman, R.A., Pettersson, C., Svensson, L., Kimia Kohestani, Karin Stinesen Bratt, Wallström, J., Månsson, M., Mikael Hellström and Hugosson, J. (2023). Men’s Acceptance of Screening for Prostate Cancer with Prostate-specific Antigen, Magnetic Resonance Imaging, and Prostate Biopsy. *European Urology Oncology*. https://doi.org/10.1016/j.euo.2023.11.003.

Goel, V., Gray, R., Chart, P., Fitch, M., Saibil, F. and Zdanowicz, Y. (2004). Perspectives on colorectal cancer screening: a focus group study. *Health Expectations*, 7(1), pp.51–60. https://doi.org/10.1046/j.1369-6513.2003.00252.x.

Gottschlich, A., Nuntadusit, T., Zarins, K.R., Hada, M., Chooson, N., Bilheem, S., Navakanitworakul, R., Nittayaboon, K., Virani, S., Rozek, L., Sriplung, H. and Meza, R. (2019). Barriers to cervical cancer screening and acceptability of HPV self-testing: a cross-sectional comparison between ethnic groups in Southern Thailand. *BMJ Open*, 9(11), p.e031957. https://doi.org/10.1136/bmjopen-2019-031957.

Gottschlich, A., Rivera-Andrade, A., Grajeda, E., Alvarez, C., Mendoza Montano, C. and Meza, R. (2017). Acceptability of Human Papillomavirus Self-Sampling for Cervical Cancer Screening in an Indigenous Community in Guatemala. *Journal of global oncology*, [online] 3(5), pp.444–454. https://doi.org/10.1200/JGO.2016.005629.

Greer, K.B., Blum, A.E., Faulx, A.L., Deming, E.M., Hricik, L.L., Siddiqui, H., Wilson, B.M. and Chak, A. (2024). Non-endoscopic screening for Barrett’s esophagus and Esophageal Adenocarcinoma in at risk Veterans. *The American Journal of Gastroenterology*. https://doi.org/10.14309/ajg.0000000000002962.

Grigore, M., Vasilache, I.-A., Cianga, P., Constantinescu, D., Duma, O., Matasariu, R.D. and Scripcariu, I.-S. (2022). Acceptability of Human Papilloma Virus Self-Sampling for Cervical Cancer Screening in a Cohort of Patients from Romania (Stage 2). *Journal of Clinical Medicine*, 11(9), p.2503. https://doi.org/10.3390/jcm11092503.

Gutierrez, F. and Lopez, L. (2025). Early Detection of Cancer and Precancerous Lesions in Persons With HIV Through a Comprehensive Cancer Screening Protocol. *Clinical Infectious Diseases*, *80*(2), 371-380.

Haarsager, J., Legerton, P., DeBats, C., Austin, G. and Vardon, P. (2023). Perceptions and implications for cervical screening and self‐collection: A qualitative exploration of never and under‐screened Queensland women. *Health Promotion Journal of Australia*. https://doi.org/10.1002/hpja.708.

Hahn, E.E., Munoz-Plaza, C., Altman, D.E., Hsu, C., Cannizzaro, N.T., Ngo-Metzger, Q., Wride, P., Gould, M.K., Mittman, B.S., Hodeib, M., Tewari, K.S., Ajamian, L.H., Eskander, R.N., Tewari, D. and Chao, C.R. (2021). De-implementation and substitution of clinical care processes: stakeholder perspectives on the transition to primary human papillomavirus (HPV) testing for cervical cancer screening. *Implementation Science Communications*, 2(1). https://doi.org/10.1186/s43058-021-00211-z.

Haile, E.L., Cindy, S., Ina, B., Belay, G., Jean-Pierre, V. geertruyden, Sharon, R., Lisbeth, L.R. and Paul, B.J. (2019). HPV testing on vaginal/cervical nurse-assisted self-samples versus clinician-taken specimens and the HPV prevalence, in Adama Town, Ethiopia. *Medicine*, 98(35), p.e16970. https://doi.org/10.1097/md.0000000000016970.

Halligan, S., Dadswell, E., Wooldrage, K., Wardle, J., von Wagner, C., Lilford, R., Yao, G.L., Zhu, S. and Atkin, W. (2015). Computed tomographic colonography compared with colonoscopy or barium enema for diagnosis of colorectal cancer in older symptomatic patients: two multicentre randomised trials with economic evaluation (the SIGGAR trials). *Health Technology Assessment*, [online] 19(54), pp.1–134. https://doi.org/10.3310/hta19540.

Hang, S., Haruyama, R., Uy, K., Fujita, N., Kimura, T., Kanal Koum and Kawana, K. (2023). Feasibility, accuracy and acceptability of self-sampled human papillomavirus testing using careHPV in Cambodia: a cross-sectional study. *Journal of Gynecologic Oncology*, [online] 35. <https://doi.org/10.3802/jgo.2024.35.e6>.

Hanley, S. J., Fujita, H., Yokoyama, S., Kunisawa, S., Tamakoshi, A., Dong, P., Kobayashi, N., Watari, H., Kudo, M., & Sakuragi, N. (2016). HPV self-sampling in Japanese women: A feasibility study in a population with limited experience of tampon use. *Journal of medical screening*, *23*(3), 164–170. <https://doi.org/10.1177/0969141315625702>

Harvey-Kelly, L.L.W., Harrison, H., Rossi, S.H., Griffin, S.J., Stewart, G.D. and Usher-Smith, J.A. (2020). Public attitudes towards screening for kidney cancer: an online survey. *BMC Urology*, 20(1). https://doi.org/10.1186/s12894-020-00724-0.

Hawkins, R., McWilliams, L., Ulph, F., Evans, D.G. and French, D.P. (2022). Healthcare professionals’ views following implementation of risk stratification into a national breast cancer screening programme. *BMC Cancer*, 22(1). https://doi.org/10.1186/s12885-022-10134-0.

He, L. and He, J. (2020). Attitudes towards HPV self-sampling among women in Chengdu, China: A cross-sectional survey. *Journal of Medical Screening*, 27(4), pp.201–206. https://doi.org/10.1177/0969141319895543.

He, X., Schifferdecker, K.E., Ozanne, E.M., Tosteson, A.N.A., Woloshin, S. and Schwartz, L.M. (2018). How Do Women View Risk-Based Mammography Screening? A Qualitative Study. *Journal of General Internal Medicine*, 33(11), pp.1905–1912. https://doi.org/10.1007/s11606-018-4601-9.

Heid-Picard, B., Cochand-Priollet, B., Rozenberg, F., Giang-Phang, D., Viard, J.-P., La Torre, V. and Ghosn, J. (2021). Ambulatory anal self-sampling in MSM living with HIV, an acceptable and reliable screening method. *PLOS ONE*, 16(2), p.e0246338. https://doi.org/10.1371/journal.pone.0246338.

Hernández-López, R., Hermosillo, L.J., León-Maldonado, L., Velázquez-Cruz, R., Torres-Ibarra, L., Lazcano-Ponce, E., Lorincz, A.T., Wheeler, C.M., Bosch, F., Cuzick, J., Rivera-Paredez, B., Nedjai, B. and Salmerón, J. (2021). Performance of an affordable urine self-sampling method for human papillomavirus detection in Mexican women. *PLOS One*, 16(7), pp.e0254946–e0254946. https://doi.org/10.1371/journal.pone.0254946.

Hill, E., Nemec, M., Marlow, L., Sherman, S.M. and Waller, J. (2020). Maximising the acceptability of extended time intervals between screens in the NHS Cervical Screening Programme: An online experimental study. *Journal of Medical Screening*, 28(3), pp.333–340. https://doi.org/10.1177/0969141320970591.

Hinkes, S., Ciraldo, K., Kobetz, E., Bartholomew, T.S., Rinehart, S., Siringo, N., Barnett, R., Godbole, N., Jeanty, F., Frederick, M. and Tookes, H.E. (2024). Opportunities for cancer prevention at syringe services programs: acceptability of HPV self-sampling and vaccination among people who inject drugs. *Harm reduction journal*, 21(1). https://doi.org/10.1186/s12954-024-00982-3.

Hol, L., de Jonge, V., van Leerdam, M.E., van Ballegooijen, M., Looman, C.W.N., van Vuuren, A.J., Reijerink, J.C.I.Y., Habbema, J.D.F., Essink-Bot, M.L. and Kuipers, E.J. (2010). Screening for colorectal cancer: Comparison of perceived test burden of guaiac-based faecal occult blood test, faecal immunochemical test and flexible sigmoidoscopy. *European Journal of Cancer*, 46(11), pp.2059–2066. https://doi.org/10.1016/j.ejca.2010.03.022.

Holman, L.L., Lu, K.H., Bast, R.C., Hernandez, M.A., Bodurka, D.C., Skates, S. and Sun, C.C. (2014). Risk perception, worry, and test acceptance in average-risk women who undergo ovarian cancer screening. *American Journal of Obstetrics and Gynecology*, [online] 210(3), pp.257.e1–257.e6. https://doi.org/10.1016/j.ajog.2013.11.022.

Horo, A.G., Didi-Kouko Coulibaly, J., Koffi, A., Tchounga, B., Seni, K., Aka, K.E. and Kone, M. (2015). Cervical Cancer Screening Program by Visual Inspection: Acceptability and Feasibility in Health Insurance Companies. *Obstetrics and Gynecology International*, 2015, pp.1–4. https://doi.org/10.1155/2015/798453.

Horsham, C., Snoswell, C., Vagenas, D., Loescher, Lois J., Gillespie, N., Soyer, H. Peter and Janda, M. (2020). Is Teledermoscopy Ready to Replace Face-to-Face Examinations for the Early Detection of Skin Cancer? Consumer Views, Technology Acceptance, and Satisfaction with Care. *Dermatology*, 236(2), pp.90–96. https://doi.org/10.1159/000506154.

Howard, M., Lytwyn, A., Lohfeld, L., Redwood-Campbell, L., Fowler, N. and Karwalajtys, T. (2009). Barriers to Acceptance of Self-sampling for Human Papillomavirus across Ethnolinguistic Groups of Women. *Canadian Journal of Public Health*, 100(5), pp.365–369. https://doi.org/10.1007/bf03405272.

Huang, M., Sun, C., Boyd-Rogers, S., Burzawa, J., Milbourne, A., Keeler, E., Yzquierdo, R., Lynch, P., Peterson, S.K. and Lu, K. (2011). Prospective Study of Combined Colon and Endometrial Cancer Screening in Women With Lynch Syndrome: A Patient-Centered Approach. *Journal of Oncology Practice*, 7(1), pp.43–47. https://doi.org/10.1200/jop.2010.000038.

Ibáñez, R., Roura, E., Acera, A., Andújar, M., Pavón, M.À., Bruni, L. and de Sanjosé, S. (2023). HPV self-sampling among cervical cancer screening users in Spain: A randomized clinical trial of on-site training to increase the acceptability. *Preventive Medicine*, [online] 173, p.107571. https://doi.org/10.1016/j.ypmed.2023.107571.

Ibrahim, A., Rasch, V., Pukkala, E. and Aro, A.R. (2011). Cervical cancer risk factors and feasibility of visual inspection with acetic acid screening in Sudan. *International Journal of Women’s Health*, p.117. https://doi.org/10.2147/ijwh.s14189.

Igidbashian, S., Boveri, S., Spolti, N., Radice, D., Sandri, M.T. and Sideri, M. (2011). Self-Collected Human Papillomavirus Testing Acceptability: Comparison of Two Self-Sampling Modalities. *Journal of Women’s Health*, 20(3), pp.397–402. https://doi.org/10.1089/jwh.2010.2189.

Ilangovan, K., Kobetz, E., Koru-Sengul, T., Marcus, E.N., Rodriguez, B., Alonzo, Y. and Carrasquillo, O. (2016). Acceptability and Feasibility of Human Papilloma Virus Self-Sampling for Cervical Cancer Screening. *Journal of Women’s Health*, 25(9), pp.944–951. https://doi.org/10.1089/jwh.2015.5469.

Jagatap, M., Maurya, A., Pandya, B., Brahmachari, S. and Singh, R. (2025). Acceptability and Determinants of Opportunistic Screening for Breast Cancer in Indian Women. *Asian Pacific Journal of Cancer Prevention*, 26(1), pp.43–47. https://doi.org/10.31557/apjcp.2025.26.1.43.

Jenkinson, G.P., Houghton, N., Zalk, N. van, Waller, J., Bello, F. and Tzemanaki, A. (2023). Acceptability of Automated Robotic Clinical Breast Examination: Survey Study. *Journal of Participatory Medicine*, [online] 15(1), p.e42704. https://doi.org/10.2196/42704.

Ji, X., Hao, M., Wang, Y., Pan, Z., Yang, R., Wang, X., Wang, H., Zhang, C., Zhang, Y., Zhang, X., Zhang, Y., Yang, G., Sarendalai, Tunala and Miao, J. (2024). HPV self-sampling for cervical cancer screening in China: A multi-center study. *Heliyon*, 10(21), p.e39544. https://doi.org/10.1016/j.heliyon.2024.e39544.

Jones, H.E., Brudney, K., Sawo, D.J., Lantigua, R. and Westhoff, C.L. (2012). The Acceptability of a Self-Lavaging Device Compared to Pelvic Examination for Cervical Cancer Screening Among Low-Income Women. *Journal of Women s Health*, 21(12), pp.1275–1281. https://doi.org/10.1089/jwh.2012.3512.

Joseph, J., Demke, O., Ameyan, L., Bitilinyu-Bango, J., Bourgoin, B., Diop, M., Guèye, B., Kama, J., Lubega, M., Madzima, B., Maparo, T., Mhizha, T., Musoke, A., Nabadda, S., Phiri, T., Tchereni, T., Wiwa, O., Hariharan, K., Peter, T. and Khan, S. (2023). Human papillomavirus testing using existing nucleic acid testing platforms to screen women for cervical cancer: implementation studies from five sub-Saharan African countries. *BMJ Open*, [online] 13(1), p.e065074. https://doi.org/10.1136/bmjopen-2022-065074.

Joseph, N.T., Namuli, A., Kakuhikire, B., Baguma, C., Juliet, M., Ayebare, P., Ahereza, P., Tsai, A.C., Siedner, M.J., Randall, T.R., Ngonzi, J. and Boatin, A.A. (2021). Implementing community-based human papillomavirus self-sampling with SMS text follow-up for cervical cancer screening in rural, southwestern Uganda. *Journal of Global Health*, 11. https://doi.org/10.7189/jogh.11.04036.

Joshi, S., Kumar, B.K., Ramshankar, V., Maitra, N., Palit, S.P., Ravindran, S. and Shyam, T.S. (2024). Performance of HPV Self-Sample Collected by a Novel Kit in Comparison with Clinician Collected Sample for Cervical Cancer Screening. *Asian Pacific Journal of Cancer Prevention*, 25(12), pp.4211–4216. https://doi.org/10.31557/apjcp.2024.25.12.4211.

Kahn, J.A. (2005). Acceptability of human papillomavirus self testing in female adolescents. *Sexually Transmitted Infections*, 81(5), pp.408–414. https://doi.org/10.1136/sti.2004.012047.

Kaoma, M., Olayemi, O., Mwaba, M.H. and Sikwewa, K. (2023). Utilizing first void urine for high-risk HPV testing for cervical cancer screening in HIV-positive women in Katete, Zambia. *BMC Women s Health*, 23(1). https://doi.org/10.1186/s12905-023-02212-7.

Karjalainen, L., Anttila, A., Nieminen, P., Luostarinen, T. and Virtanen, A. (2016). Self-sampling in cervical cancer screening: comparison of a brush-based and a lavage-based cervicovaginal self-sampling device. *BMC Cancer*, 16(1). https://doi.org/10.1186/s12885-016-2246-9.

Katanga, J., Rasch, V., Manongi, R., Pembe, A.B., Mwaiselage, J. and Kjaer, S.K. (2021). Concordance in HPV Detection Between Self-Collected and Health Provider–Collected Cervicovaginal Samples Using careHPV in Tanzanian Women. (7), pp.985–991. https://doi.org/10.1200/go.20.00598.

Kaufman, E., Castro, C. de, Williamson, T., Lessard, B., Munoz, M., Mayrand, M.H., Burchell, A.N., Klein, M.B., Charest, L., Auger, M., Marcus, V., F. Coutlée and A. de Pokomandy (2020). Acceptability of Anal Cancer Screening Tests for Women Living with HIV in the EVVA Study. *Current Oncology*, 27(1), pp.19–26. https://doi.org/10.3747/co.27.5401.

Kellen, E., Gabriels, S., Van Hal, G. and Goossens, M.C. (2020). Lung cancer screening: intention to participate and acceptability among Belgian smokers. *European Journal of Cancer Prevention*, 30(6), pp.457–461. https://doi.org/10.1097/cej.0000000000000656.

Kelley-Jones, C., Scott, S. and Waller, J. (2021). UK Women’s Views of the Concepts of Personalised Breast Cancer Risk Assessment and Risk-Stratified Breast Screening: A Qualitative Interview Study. *Cancers*, 13(22), p.5813. https://doi.org/10.3390/cancers13225813.

Kelley-Jones, C., Scott, S.E. and Waller, J. (2024). Acceptability of de-intensified screening for women at low risk of breast cancer: a randomised online experimental survey. *BMC Cancer*, 24(1). https://doi.org/10.1186/s12885-024-12847-w.

Kelley-Jones, C., Scott, S., Pashayan, N., Morris, S., Yasmina Okan and Waller, J. (2024). Risk-Adapted Breast Screening for Women at Low Predicted Risk of Breast Cancer: An Online Discrete Choice Experiment. *Medical Decision Making*, 44(5), pp.586–600. https://doi.org/10.1177/0272989x241254828.

Ketelaars, P.J.W., Bosgraaf, R.P., Siebers, A.G., Massuger, L.F.A.G., van der Linden, J.C., Wauters, C.A.P., Rahamat-Langendoen, J.C., van den Brule, A.J.C., IntHout, J., Melchers, W.J.G. and Bekkers, R.L.M. (2017). High-risk human papillomavirus detection in self-sampling compared to physician-taken smear in a responder population of the Dutch cervical screening: Results of the VERA study. *Preventive Medicine*, [online] 101, pp.96–101. https://doi.org/10.1016/j.ypmed.2017.05.021.

Kew, F.M., Ashton, V.J. and Cruickshank, D.J. (2004). Tolerability of Transvaginal Ultrasonography as an Ovarian Cancer Screening Test. *Journal of Medical Screening*, 11(1), pp.45–47. https://doi.org/10.1177/096914130301100110.

Khoja, L., Torres, H.G., Hak, L., Manar Aljebori, Minh Tung Phung, Sakleh, A., Stiffler, M., Tariq, M. and Celeste Leigh Pearce (2024a). Cervical cancer screening and vaccination acceptability and attitudes among Arab American women in Southeastern Michigan: a qualitative study. *Scientific reports*, 14(1). https://doi.org/10.1038/s41598-024-64462-1.

Khoja, L., Wang, Y., Haque, S.E., Ahsan, H., Islam, T., Munshi, S.U., Hasan, A.K.M.R., Islam, M.T., Jharna, A.B. and Pearce, C.L. (2024b). Understanding of cervical cancer, acceptability of HPV self-collection, and prevalence of HPV in a semi-urban setting in Bangladesh. *PLOS global public health*, [online] 4(4), p.e0003157. https://doi.org/10.1371/journal.pgph.0003157.

Khoo, S.P., Lim, W.T., Rajasuriar, R., Nasir, N.H., Gravitt, P.E. and Yin Ling Woo (2021). The Acceptability and Preference of Vaginal Self-sampling for Human Papillomavirus (HPV) Testing among a Multi-ethnic Asian Female Population. *Cancer Prevention Research*, 14(1), pp.105–112. https://doi.org/10.1158/1940-6207.capr-20-0280.

Khuhaprema, T., Sangrajrang, S., Lalitwongsa, S., Chokvanitphong, V., Raunroadroong, T., Ratanachu-ek, T., Muwonge, R., Lucas, E., Wild, C.P. and Rengaswamy Sankaranarayanan (2014). Organised colorectal cancer screening in Lampang Province, Thailand: preliminary results from a pilot implementation programme. *BMJ Open*, 4(1), pp.e003671–e003671. https://doi.org/10.1136/bmjopen-2013-003671.

KIRBY, R.S., KIRBY, M.G., FENELEY, M.R., McNICHOLAS, T., McLEAN, A. and WEBB, J.A.W. (1994). Screening for carcinoma of the prostate: a GP based study. *British Journal of Urology*, 74(1), pp.64–71. https://doi.org/10.1111/j.1464-410x.1994.tb16549.x.

Kjome, R.L.S., Wright, D.J., Bjaaen, A.-K.B., Garstad, K.W. and Valeur, M. (2017). Dermatological cancer screening: Evaluation of a new community pharmacy service. *Research in Social and Administrative Pharmacy*, 13(6), pp.1214–1217. https://doi.org/10.1016/j.sapharm.2016.12.001.

Klaaborg, K., Madsen, M.S., Søndergaard, O. and Kronborg, O. (1986). Participation in Mass Screening for Colorectal Cancer with Fecal Occult Blood Test. *Scandinavian Journal of Gastroenterology*, 21(10), pp.1180–1184. https://doi.org/10.3109/00365528608996440.

Kluhsman, B., Lengerich, E., Fleisher, L., Paskett, E., Miller-Halegoua, S. and Balshem, A. (2012). A Pilot Study for Using Fecal Immunochemical Testing to Increase Colorectal Cancer Screening in Appalachia, 2008-2009. *Preventing Chronic Disease*. https://doi.org/10.5888/pcd9.110160.

Kohler, R.E., Elliott, T., Monare, B., Moshashane, N., Ramontshonyana, K., Chatterjee, P., Ramogola‐Masire, D. and Morroni, C. (2019). HPV self‐sampling acceptability and preferences among women living with HIV in Botswana. *International Journal of Gynecology & Obstetrics*, 147(3), pp.332–338. https://doi.org/10.1002/ijgo.12963.

Koitsalu, M., Sprangers, M.A.G., Eklund, M., Czene, K., Hall, P., Grönberg, H. and Brandberg, Y. (2015). Public interest in and acceptability of the prospect of risk-stratified screening for breast and prostate cancer. *Acta Oncologica*, 55(1), pp.45–51. https://doi.org/10.3109/0284186x.2015.1043024.

Kola‐Palmer, S., Rogers, M., Halliday, A. and Rickford, R. (2022). ‘A lot can happen in five years’: Women’s attitudes to extending cervical screening intervals. *European Journal of Cancer Care*. https://doi.org/10.1111/ecc.13655.

Lack, N., West, B. and Jeffries, D. (2005). Comparison of non-invasive sampling methods for detection of HPV in rural African women. *Sexually Transmitted Infections*, 81(3), pp.239–241. https://doi.org/10.1136/sti.2004.010413.

Lam, J., Barnell, G., Merchant, M., Ellis, C. and Silverberg, M. (2018). Acceptability of high‐resolution anoscopy for anal cancer screening in HIV‐infected patients. *HIV Medicine*, 19(10), pp.716–723. https://doi.org/10.1111/hiv.12663.

Landy, R., Hollingworth, T., Waller, J., Laura A.V. Marlow, Rigney, J., Round, T., Sasieni, P. and Anita (2022). Non-speculum clinician-taken samples for human papillomavirus testing: a cross-sectional study in older women. 72(721), pp.e538–e545. https://doi.org/10.3399/bjgp.2021.0708.

Laskow, B., Figueroa, R., Alfaro, K.M., Scarinci, I.C., Conlisk, E., Maza, M., Chang, J.C. and Cremer, M. (2017). A pilot study of community-based self-sampling for HPV testing among non-attenders of cervical cancer screening programs in El Salvador. *International Journal of Gynecology & Obstetrics*, 138(2), pp.194–200. https://doi.org/10.1002/ijgo.12204.

Lathrop, E., Chissano, M., Tevir, G., Chongo, Y., Cambe, E., Edson Chivambo, Hoover, H. and Bouanchaud, P. (2023). Self-sampling for human papilloma virus DNA as a strategy to increase access to cervical cancer screening: patients’ and providers’ perspectives on facilitators and barriers to scale. *PubMed*, 27(12), pp.106–111. https://doi.org/10.29063/ajrh2023/v27i12.13.

Laza-Vásquez, C., Hernández-Leal, M.J., Carles-Lavila, M., Pérez-Lacasta, M.J., Cruz-Esteve, I. and Rué, M. (2022). Barriers and Facilitators to the Implementation of a Personalized Breast Cancer Screening Program: Views of Spanish Health Professionals. *International Journal of Environmental Research and Public Health*, 19(3), p.1406. https://doi.org/10.3390/ijerph19031406.

Le Goff, J., Le Duc-Banaszuk, A.-S., Lefeuvre, C., Pivert, A., Ducancelle, A., De Pauw, H., Arbyn, M., Vinay, A. and Rexand-Galais, F. (2023). Acceptability to Healthcare Professionals of Home-Based HPV Self-Sampling for Cervical Screening: A French Qualitative Study Conducted in an Area with Low Access to Health Services. *Cancers*, [online] 15(21), p.5163. https://doi.org/10.3390/cancers15215163.

Le Goff, J., Le Duc-Banaszuk, A.-S., Lefeuvre, C., Pivert, A., Ducancelle, A., Hélène de Pauw, Arbyn, M., Aubeline Vinay and Franck Rexand-Galais (2024). Acceptance of Self-Sampling by Women Not Regularly Participating in Cervical Cancer Screening in Areas with Low Medical Density: A Qualitative Study within the French CapU4 Trial. *Cancers*, 16(11), pp.2066–2066. https://doi.org/10.3390/cancers16112066.

Le, A., Rohweder, C., Wheeler, S.B., Jennifer Elston Lafata, Teal, R., Giannone, K., Zaffino, M. and Smith, J.S. (2023). Self-Collection for Primary HPV Testing: Perspectives on Implementation From Federally Qualified Health Centers. *Preventing Chronic Disease*, 20. https://doi.org/10.5888/pcd20.230056.

Le, D., Coriolan Ciceron, A., Jeon, M.J., Gonzalez, L.I., Jordan, J.A., Bordon, J. and Long, B. (2022). Cervical Cancer Prevention and High-Risk HPV Self-Sampling Awareness and Acceptability among Women Living with HIV: A Qualitative Investigation from the Patients’ and Providers’ Perspectives. *Current Oncology*, 29(2), pp.516–533. https://doi.org/10.3390/curroncol29020047.

Lechner, S.C., Pereira, L., Reategui, E., Gordon, C., Byrne, M., Hooper, M.W., Lee, D.J., Abouyared, M. and Franzmann, E. (2014). Acceptability of a Rinse Screening Test for Diagnosing Head and Neck Squamous Cell Carcinoma Among Black Americans. *Journal of Racial and Ethnic Health Disparities*, 2(1), pp.62–67. https://doi.org/10.1007/s40615-014-0048-9.

Lee, F., Bula, A., Chapola, J., Mapanje, C., Phiri, B., Kamtuwange, N., Tsidya, M., Tang, J. and Chinula, L. (2021). Women’s experiences in a community-based screen-and-treat cervical cancer prevention program in rural Malawi: a qualitative study. *BMC Cancer*, 21(1). https://doi.org/10.1186/s12885-021-08109-8.

Leng, J., Li, R., Lui, F. and Gany, F. (2020). Healthcare Professionals’ Perspectives on Adapting a Community Health Worker Model to Facilitate Lung Cancer Screening for Chinese For-Hire Vehicle Drivers. *Journal of Cancer Education*. https://doi.org/10.1007/s13187-020-01813-2.

León-Maldonado, L., Cabral, A., Brown, B., Ryan, G., Maldonado, A., Salmerón, J., Allen-Leigh, B. and Lazcano-Ponce, E. (2019). Feasibility of a combined strategy of HPV vaccination and screening in Mexico: the FASTER-Tlalpan study experience. *Human Vaccines & Immunotherapeutics*, 15(7-8), pp.1986–1994. https://doi.org/10.1080/21645515.2019.1619401.

Levin, J.R., Hirsch, S.H., Bastani, R., Ganz, P.A., Lovett, M.L. and Reuben, D.B. (1997). Acceptability of Mobile Mammography Among Community-Dwelling Older Women. *Journal of the American Geriatrics Society*, 45(11), pp.1365–1370. https://doi.org/10.1111/j.1532-5415.1997.tb02938.x.

Levinson, K.L., Jernigan, A.M., Flocke, S.A., Tergas, A.I., Gunderson, C.C., Huh, W.K., Wilkinson-Ryan, I., Lawson, P.J., Fader, A.N. and Belinson, J.L. (2016). Intimate Partner Violence and Barriers to Cervical Cancer Screening. *Journal of Lower Genital Tract Disease*, 20(1), pp.47–51. https://doi.org/10.1097/lgt.0000000000000153.

Li, J., Wu, R., Qu, X., Huang, X., Li, L., Lin, Z., Zhang, Z., Deng, J., Liu, R., Zhao, X., Zhang, S., Lin, B., An, R., Zhao, C., Li, M., Zhao, Y. and Wei, L. (2022). Effectiveness and feasibility of self-sampling for human papillomavirus testing for internet-based cervical cancer screening. *Frontiers in Public Health*, 10. https://doi.org/10.3389/fpubh.2022.938272.

Li, M., Nyabigambo, A., Navvuga, P., Nuwamanya, E., Nuwasiima, A., Kaganda, P., Asiimwe, F.T., Vodicka, E., Mugisha, N.M., Mukose, A., Kwesiga, D.K., Lubinga, S.J., Garrison, L.P. and Babigumira, J.B. (2017). Acceptability of cervical cancer screening using visual inspection among women attending a childhood immunization clinic in Uganda. *Papillomavirus research (Amsterdam, Netherlands)*, [online] 4, pp.17–21. https://doi.org/10.1016/j.pvr.2017.06.004.

Lichtenfels, M., Lorenzi, N.P.C., Tacla, M., Yokochi, K., Frustockl, F., Silva, C.A., Silva, A.L. da, Termini, L. and Farias, C.B. (2023). A New Brazilian Device for Cervical Cancer Screening: Acceptability and Accuracy of Self-sampling. *Revista Brasileira de Ginecologia e Obstetrícia*, [online] 45, pp.235–241. <https://doi.org/10.1055/s-0043-1770134>.

Liebermann, E., Van Devanter, N., Frías Gúzman, N., Hammer, M. J., & Ompad, D. (2021). Dominican Provider Attitudes Towards HPV Testing for Cervical Cancer Screening and, Current Challenges to Cervical Cancer Prevention in the Dominican Republic: a Mixed Methods Study. *Journal of cancer education*, *36*(6), 1170–1185. <https://doi.org/10.1007/s13187-020-01746-w>

Lim, A.W., Hollingworth, A., Kalwij, S., Curran, G. and Sasieni, P. (2016). Offering self-sampling to cervical screening non-attenders in primary care. *Journal of Medical Screening*, 24(1), pp.43–49. <https://doi.org/10.1177/0969141316639346>.

Liow, J. J. K., Lim, Z. L., Sim, T. M. Y., Ho, P. J., Goh, S. A., Choy, S. D., Chew, Y. J., Tan, B. K., Tan, V. K. M., Hartman, M., McCrickerd, K., & Li, J. (2022). *"It Will Lead You to Make Better Decisions about Your Health"*-A Focus Group and Survey Study on Women's Attitudes towards Risk-Based Breast Cancer Screening and Personalised Risk Assessments. *Current oncology (Toronto, Ont.)*, *29*(12), 9181–9198. <https://doi.org/10.3390/curroncol29120719>

Lippey, J., Keogh, L., Campbell, I., Mann, G.B. and Forrest, L.E. (2023). Impact of a risk based breast screening decision aid on understanding, acceptance and decision making. *npj Breast Cancer*, 9(1). https://doi.org/10.1038/s41523-023-00569-4.

Lippey, J., Keogh, L.A., Mann, G.B., Campbell, I.G. and Forrest, L.E. (2019). ‘A Natural Progression’: Australian Women’s Attitudes About an Individualized Breast Screening Model. *Cancer Prevention Research*, 12(6), pp.383–390. https://doi.org/10.1158/1940-6207.capr-18-0443.

Lofters, A., Vahabi, M., Fardad, M. and Raza, A. (2017). Exploring the acceptability of human papillomavirus self-sampling among Muslim immigrant women. *Cancer Management and Research*, Volume 9, pp.323–329. https://doi.org/10.2147/cmar.s139945.

López, D.D., Acosta Bedón, A., Rivas-Párraga, R., Heredia, M., Muñoz, C., Vega Crespo, B., Vermandere, H., Hendrickx, M., Gama, A. and Neira, V.A. (2025). Assessing the Acceptability of a Vaginal Self-Sampling Device in a Rural Parish of Cuenca, Ecuador. *Journal of Primary Care & Community Health*, [online] 16. https://doi.org/10.1177/21501319251320172.

Lorenzi, N.P.C., Termini, L., Longatto Filho, A., Tacla, M., de Aguiar, L.M., Beldi, M.C., Ferreira-Filho, E.S., Baracat, E.C. and Soares-Júnior, J.M. (2019). Age-related acceptability of vaginal self-sampling in cervical cancer screening at two university hospitals: a pilot cross-sectional study. *BMC Public Health*, 19(1). https://doi.org/10.1186/s12889-019-7292-1.

Lowe, J.B. (2004). Acceptability and feasibility of a community-based screening programme for melanoma in Australia. *Health Promotion International*, 19(4), pp.437–444. https://doi.org/10.1093/heapro/dah405.

Luckett, R., Ramogola-Masire, D., Harris, D.A., Gompers, A., Gaborone, K., Mochoba, L., Ntshese, L., Mathoma, A., Kula, M., Shapiro, R. and Larson, E. (2024). Feasibility and acceptability of an HPV self-testing strategy: lessons from a research context to assess for ability to implement into primary care at a national level in Botswana. *Frontiers in Global Women’s Health*, 4. https://doi.org/10.3389/fgwh.2023.1300788.

Luu, X.Q., Lee, K., Lee, Y.Y., Suh, M., Kim, Y. and Choi, K.S. (2020). Acceptance on colorectal cancer screening upper age limit in South Korea. *World Journal of Gastroenterology*, 26(27), pp.3963–3974. https://doi.org/10.3748/wjg.v26.i27.3963.

MacLaughlin, K.L., Jenkins, G.D., St Sauver, J., Fan, C., Miller, N.E., Meyer, A.F., Jacobson, R.M. and Finney Rutten, L.J. (2024). Primary human papillomavirus testing by clinician- versus self-collection: Awareness and acceptance among cervical cancer screening-eligible women. *Journal of Medical Screening*, 31(4), pp.223–231. https://doi.org/10.1177/09691413241260019.

Madding, R.A., Currier, J.J., Yanit, K., Hedges, M. and Bruegl, A. (2024). HPV self-collection for cervical cancer screening among survivors of sexual trauma: a qualitative study. *BMC Women s Health*, 24(1). https://doi.org/10.1186/s12905-024-03301-x.

Madhivanan, P., Nishimura, H., Ravi, K., Pope, B., Coudray, M., Arun, A., Krupp, K., Jayakrishna, P. and Srinivas, V. (2021). Acceptability and Concordance of Self- Versus Clinician- Sampling for HPV Testing among Rural South Indian Women. *Asian Pacific Journal of Cancer Prevention*, 22(3), pp.971–976. https://doi.org/10.31557/apjcp.2021.22.3.971.

Mahande, M.J., Oneko, O., Amour, C., Pollie, M., Smith, C., Mboya, I.B. and Finkel, M. (2021). Feasibility and acceptability of human papillomavirus self‐sampling in a semi‐urban area in northern Tanzania. *International Journal of Gynecology & Obstetrics*, 154(1), pp.113–118. https://doi.org/10.1002/ijgo.13579.

Mahomed, K., Evans, D., Sauls, C., Richter, K., Smith, J. and Firnhaber, C. (2014). Human papillomavirus (HPV) testing on self-collected specimens: perceptions among HIV positive women attending rural and urban clinics in South Africa. *Pan African Medical Journal*, 17. https://doi.org/10.11604/pamj.2014.17.189.3454.

Maitanmi, J.O., Fabiyi, T.E., Oluwadara Eniola, Sansi, T.O., Blessing, J.O., Maitanmi, B.T., Ojewale, M.O., Dairo, A.A., Adebiyi, D.A. and Oluwadamilare Akingbade (2023). Knowledge and acceptability of cervical cancer screening among female undergraduates in Babcock University Ilishan-Remo, Ogun State, Nigeria. [online] 17, pp.1502–1502. https://doi.org/10.3332/ecancer.2023.1502.

Mao, C., Kulasingam, S.L., Whitham, H.K., Hawes, S.E., Lin, J. and Kiviat, N.B. (2017). Clinician and Patient Acceptability of Self-Collected Human Papillomavirus Testing for Cervical Cancer Screening. *Journal of Women’s Health*, [online] 26(6), pp.609–615. https://doi.org/10.1089/jwh.2016.5965.

Marandu, G., Laisser, R., Malale, K. and Rambau, P. (2024). Factors Associated With Uptake of Breast Cancer Screening Among Catholic Nuns in Lake Zone, Tanzania. *International Journal of Breast Cancer*, 2024(1). https://doi.org/10.1155/2024/5024392.

Maricela, G., Augusto, O., Gil, M., Sarahí, D. and Champion, J.D. (2021). Attitude and Acceptability of the Self-Sampling in HPV Carrier Women. *Hispanic Health Care International*, 20(1), pp.40–43. https://doi.org/10.1177/15404153211001577.

Marjoram, J., Strachan, R., Allan, A. and Allan, E. (1996). Screening for colorectal cancer: a general-practice-based study. *PubMed*, 46(406), pp.283–86.

Marlow, L., Nemec, M., Barnes, J. and Waller, J. (2022). Testing key messages about extending cervical screening intervals. *Patient Education and Counseling*, 105(8), pp.2757–2762. https://doi.org/10.1016/j.pec.2022.04.006.

Martello, G., Gori, S., Frayle, H., Franceschi, S., Zorzi, M. and Annarosa Del Mistro (2023). Acceptability of deferring the start of cervical cancer screening to age 30 for women vaccinated against human papillomavirus. *Preventive Medicine Reports*, [online] 36, pp.102438–102438. https://doi.org/10.1016/j.pmedr.2023.102438.

Martin, J.A., Crotty, B., Barbaro, D., Higlett, T. and Zalcberg, J. (2001). Training general practitioners in flexible sigmoidoscopy to screen for colorectal cancer. *ANZ Journal of Surgery*, 71(12), pp.715–719. https://doi.org/10.1046/j.1445-1433.2001.02273.x.

Maza, M., Melendez, M., Masch, R., Alfaro, K., Chacon, A., Gonzalez, E., Soler, M., Conzuelo-Rodriguez, G., Gage, J.C., Alonzo, T.A., Castle, P.E., Felix, J.C. and Cremer, M. (2018). Acceptability of self-sampling and human papillomavirus testing among non-attenders of cervical cancer screening programs in El Salvador. *Preventive Medicine*, 114, pp.149–155. https://doi.org/10.1016/j.ypmed.2018.06.017.

McFarlane, S.J., Morgan, S.E. and Schlumbrecht, M. (2021). Acceptability of a multicomponent, community-based, HPV self-test intervention among Jamaican women. *Cancer Causes & Control*, 32(5), pp.547–554. https://doi.org/10.1007/s10552-021-01406-4.

McGill, F., Fields, P.J., Aví Bahadoor-Yetman, Manglardi, E.S., Bailey, R., Kritika Padala, Lendore, J., John-Ballantyne, T. and Lake, S. (2024). Investigating under-reported human papillomavirus genotypes in Grenadian women through self-sampling for cervical cancer screening. *Revista Panamericana de Salud Pública*, [online] 48, pp.1–1. https://doi.org/10.26633/rpsp.2024.62.

McRae, J., Martin, C., O’Leary, J. and Sharp, L. (2014). ‘If you can’t treat HPV, why test for it?’ Women’s attitudes to the changing face of cervical cancer prevention: a focus group study. *BMC Women’s Health*, 14(1). https://doi.org/10.1186/1472-6874-14-64.

McWilliams, L., Ruane, H., Ulph, F., Woof, V.G., Harrison, F., D. Gareth Evans and French, D.P. (2023). What do women think about having received their breast cancer risk as part of a risk-stratified NHS Breast Screening Programme? A qualitative study. *British Journal of Cancer*, 129(2), pp.356–365. https://doi.org/10.1038/s41416-023-02268-0.

McWilliams, L., Woof, V.G., Donnelly, L.S., Howell, A., Evans, D.G. and French, D.P. (2020). Risk stratified breast cancer screening: UK healthcare policy decision-making stakeholders’ views on a low-risk breast screening pathway. *BMC Cancer*, 20(1). https://doi.org/10.1186/s12885-020-07158-9.

McWilliams, L., Woof, V.G., Donnelly, L.S., Howell, A., Evans, D.G. and French, D.P. (2021). Extending screening intervals for women at low risk of breast cancer: do they find it acceptable? *BMC Cancer*, 21(1). https://doi.org/10.1186/s12885-021-08347-w.

Meisel, S.F., Pashayan, N., Rahman, B., Side, L., Fraser, L., Gessler, S., Lanceley, A. and Wardle, J. (2015). Adjusting the frequency of mammography screening on the basis of genetic risk: Attitudes among women in the UK. *Breast (Edinburgh, Scotland)*, [online] 24(3), pp.237–241. https://doi.org/10.1016/j.breast.2015.02.001.

Mensah, K., Assoumou, N., Duchesne, V., Dolorès Pourette, Pierre De Beaudrap and Dumont, A. (2020). Acceptability of HPV screening among HIV-infected women attending an HIV-dedicated clinic in Abidjan, Côte d’Ivoire. *BMC Women’s Health*, 20(1). https://doi.org/10.1186/s12905-020-01021-6.

Mensah, K., Kaboré, C., Salifou Zeba, Bouchon, M., Duchesne, V., Dolorès Pourette, DeBeaudrap, P. and Dumont, A. (2021). Implementation of HPV-based screening in Burkina Faso: lessons learned from the PARACAO hybrid-effectiveness study. *BMC Women s Health*, 21(1). https://doi.org/10.1186/s12905-021-01392-4.

Mishra, G.A., Pimple, S.A., Anand, K.V., Kulkarni, V.Y., Patil, A.S. and Biswas, S.K. (2024). Acceptability and validity of HPV self‐sampling for cervical cancer screening among women living in different ecological settings in India. *International Journal of Cancer*. https://doi.org/10.1002/ijc.35222.

Mitchell, E.M., Hall, K.M., Doede, A., Rong, A., Estrada, M.M., Granera, O.B., Maldonado, F., Kallas, H.A., Bravo-Rodriguez, C., Forero, M., Tchuisseu, Y.P. and Dillingham, R.A. (2023). Feasibility and acceptability of self-collection of Human Papillomavirus samples for primary cervical cancer screening on the Caribbean Coast of Nicaragua: A mixed-methods study. *Frontiers in Oncology*, 12. https://doi.org/10.3389/fonc.2022.1020205.

Mitchell, E.M., Lothamer, H., Garcia, C., Marais, A.D., Camacho, F., Poulter, M., Bullock, L. and Smith, J.S. (2020). Acceptability and Feasibility of Community-Based, Lay Navigator-Facilitated At-Home Self-Collection for Human Papillomavirus Testing in Underscreened Women. *Journal of Women’s Health*, 29(4), pp.596–602. https://doi.org/10.1089/jwh.2018.7575.

Mkinen, T., Auvinen, A., Hakama, M., Stenman, Ulf-H. åkan and Tammela, T.L.J. (2002). Acceptability and complications of prostate biopsy in population-based PSA screening versus routine clinical practice: a prospective, controlled study. *Urology*, 60(5), pp.846–850. https://doi.org/10.1016/s0090-4295(02)01864-2.

Mkuu, R.S., Staras, S.A., Chakrabarti, C., Hall, J., Harvey, I., Salloum, R.G., Barrow, S., Ortega, S., Woodard, J., Seals, K., Rawls, A., Meduri, Y., Donahoo, W.T., Goede, D.L. and Shenkman, E.A. (2024). Acceptability of HPV self-collection: A qualitative study of Black women living with type II diabetes and social vulnerability. *Journal of Clinical & Translational Endocrinology*, [online] 35, p.100331. <https://doi.org/10.1016/j.jcte.2024.100331>.

Molokwu, J. C., Penaranda, E., Dwivedi, A., Mallawaarachchi, I., & Shokar, N. (2018). Effect of Educational Intervention on Self-Sampling Acceptability and Follow-Up Paps in Border Dwelling Hispanic Females. *Journal of lower genital tract disease*, *22*(4), 295–301. <https://doi.org/10.1097/LGT.0000000000000424>

Montealegre, J.R., Landgren, R.M., Anderson, M.L., Hoxhaj, S., Williams, S., Robinson, D.J., Scheurer, M.E. and Ramondetta, L.M. (2015). Acceptability of self-sample human papillomavirus testing among medically underserved women visiting the emergency department. *Gynecologic Oncology*, 138(2), pp.317–322. https://doi.org/10.1016/j.ygyno.2015.05.028.

Montealegre, J.R., Mullen, P.D., L. Jibaja-Weiss, M., Vargas Mendez, M.M. and Scheurer, M.E. (2014). Feasibility of Cervical Cancer Screening Utilizing Self-sample Human Papillomavirus Testing Among Mexican Immigrant Women in Harris County, Texas: A Pilot Study. *Journal of Immigrant and Minority Health*, 17(3), pp.704–712. https://doi.org/10.1007/s10903-014-0125-5.

Montgomery, M.P., Dune, T., Shetty, P.K. and Shetty, A.K. (2014). Knowledge and Acceptability of Human Papillomavirus Vaccination and Cervical Cancer Screening among Women in Karnataka, India. *Journal of Cancer Education*, 30(1), pp.130–137. https://doi.org/10.1007/s13187-014-0745-4.

Morling, J., Barke, A., Chapman, C. and Logan, R. (2018). Could stool collection devices help increase uptake in bowel cancer screening programmes? *Journal of Medical Screening*, 25(4), pp.174–177. https://doi.org/10.1177/0969141317753463.

Moukam, A.M.D., Owono, M.S.D., Kenfack, B., Vassilakos, P., Petignat, P., Sormani, J. and Schmidt, N.C. (2021). ‘Cervical cancer screening: awareness is not enough’. Understanding barriers to screening among women in West Cameroon-a qualitative study using focus groups. *Reproductive Health*, [online] 18(1), p.147. https://doi.org/10.1186/s12978-021-01186-9.

Mremi, A., Linde, D.S., Mchome, B., Mlay, J., Schledermann, D., Blaakaer, J. and Rasch, V. (2021). Acceptability and feasibility of self-sampling and follow-up attendance after text message delivery of human papillomavirus results: A cross-sectional study nested in a cohort in rural Tanzania. *Acta Obstetricia Et Gynecologica Scandinavica*, [online] 100(4), pp.802–810. https://doi.org/10.1111/aogs.14117.

Mungo, C., Osongo, C.O., Ambaka, J., Randa, M.A., Samba, B., Ochieng, C.A., Barker, E., Guliam, A., Omoto, J. and Cohen, C.R. (2021). Feasibility and Acceptability of Smartphone-Based Cervical Cancer Screening Among HIV-Positive Women in Western Kenya. *JCO Global Oncology*, (7), pp.686–693. https://doi.org/10.1200/go.21.00013.

Murchland, A.R., Gottschlich, A., Bevilacqua, K., Pineda, A., Sandoval-Ramírez, B.A., Alvarez, C.S., Ogilvie, G.S., Carey, T.E., Prince, M., Dean, M., Mendoza Montano, C., Rivera-Andrade, A. and Meza, R. (2019). HPV self-sampling acceptability in rural and indigenous communities in Guatemala: a cross-sectional study. *BMJ Open*, 9(10), p.e029158. https://doi.org/10.1136/bmjopen-2019-029158.

Naseri, S., Young, S., Cruz, G. and Blumenthal, P.D. (2022). Screening for High-Risk Human Papillomavirus Using Passive, Self-Collected Menstrual Blood. *Obstetrics & Gynecology*, 140(3), pp.470–476. https://doi.org/10.1097/aog.0000000000004904.

Nelson, E.J., Hughes, J., Oakes, J.M., Thyagarajan, B., Pankow, J.S. and Kulasingam, S.L. (2014). Human Papillomavirus Infection in Women Who Submit Self-collected Vaginal Swabs After Internet Recruitment. *Journal of Community Health*, 40(3), pp.379–386. https://doi.org/10.1007/s10900-014-9948-1.

Němec, M., Waller, J., Barnes, J. and Laura A.V. Marlow (2022). Acceptability of extending HPV-based cervical screening intervals from 3 to 5 years: an interview study with women in England. *BMJ Open*, [online] 12(5), pp.e058635–e058635. https://doi.org/10.1136/bmjopen-2021-058635.

Ngelangel, C.A., Limson, G.M., Cordero, C.P., Abelardo, A.D., Avila, J.M. and Festin, M.R. (2003). Acetic-acid guided visual inspection vs. cytology-based screening for cervical cancer in the Philippines. *International Journal of Gynecology & Obstetrics*, 83(2), pp.141–150. https://doi.org/10.1016/s0020-7292(03)00265-0.

Ngu, S.-F., Lau, L.S., Ching Yin Chan, Hextan YS Ngan, Annie NY Cheung and Karen KL Chan (2024). Acceptability of self-collected vaginal samples for human papillomavirus testing for primary cervical cancer screening: comparison of face-to-face and online recruitment modes. *BMC public health*, 24(1). https://doi.org/10.1186/s12889-024-18551-5.

Nicholls, S.G., Etchegary, H., Carroll, J.C., Castle, D., Lemyre, L., Potter, B.K., Craigie, S. and Wilson, B.J. (2016). Attitudes to incorporating genomic risk assessments into population screening programs: the importance of purpose, context and deliberation. *BMC Medical Genomics*, 9(1). https://doi.org/10.1186/s12920-016-0186-5.

Nicholson, F.B. and Korman, M.G. (2005). Acceptance of flexible sigmoidoscopy and colonoscopy for screening and surveillance in colorectal cancer prevention. *Journal of Medical Screening*, 12(2), pp.89–95. https://doi.org/10.1258/0969141053908294.

Ninsiima, M., Nyabigambo, A. and Kagaayi, J. (2023). Acceptability of integration of cervical cancer screening into routine HIV care, associated factors and perceptions among HIV-infected women: a mixed methods study at Mbarara Regional Referral Hospital, Uganda. *BMC Health Services Research*, 23(1). https://doi.org/10.1186/s12913-023-09326-6.

Niv, Y., Bogolavski, I., Sagi Ilani, Irit Avni, Gal, E., Vilkin, A. and Levi, Z. (2012). Impact of colonoscopy on quality of life. *European Journal of Gastroenterology & Hepatology*, 24(7), pp.781–786. https://doi.org/10.1097/meg.0b013e328352deff.

Northridge, M.E., Weiserbs, K.F., Sabounchi, S.S., Torroni, A., Mohadjeri-Franck, N.S., Gargano, S., George, E., Littlejohn, T.C., Troxel, A.B., Wu, Y., Testa, P.A., Wismer, J., Zaremba, K., Tylawsky, P. and Bina, B. (2022). A feasibility and acceptability study of using an intra-oral camera and an asynchronous tele-mentoring protocol to detect and identify oral lesions. *Deleted Journal*, 11(3). https://doi.org/10.1177/22799036221115778.

O’Sullivan, E.M. (2011). Prevalence of oral mucosal abnormalities in addiction treatment centre residents in Southern Ireland. *Oral Oncology*, 47(5), pp.395–399. https://doi.org/10.1016/j.oraloncology.2011.03.003.

O’Sullivan, I. and Orbell, S. (2004). Self-Sampling in Screening to Reduce Mortality from Colorectal Cancer: A Qualitative Exploration of the Decision to Complete a Faecal Occult Blood Test (FOBT). *Journal of Medical Screening*, 11(1), pp.16–22. https://doi.org/10.1177/096914130301100105.

Obiri-Yeboah, D., Adu-Sarkodie, Y., Djigma, F., Hayfron-Benjamin, A., Abdul, L., Simpore, J. and Mayaud, P. (2017). Self-collected vaginal sampling for the detection of genital human papillomavirus (HPV) using careHPV among Ghanaian women. *BMC Women’s Health*, 17(1). https://doi.org/10.1186/s12905-017-0448-1.

Ocasio-Villa, F., Morales-Torres, L., Velez-Medina, N., Cubano, L.A., Orengo, J.C. and Edu (2022). Evaluation of the Pink Luminous Breast LED-Based Technology Device as a Screening Tool for the Early Detection of Breast Abnormalities. *Frontiers in Medicine*, 8. https://doi.org/10.3389/fmed.2021.805182.

Ogilvie, G., Smith, L.G., Niekerk, D. van , Khurshed, F., Pedersen, H., Taylor, D., Thomson, K., Greene, S.B., Babich, S.M., Franco, E.L. and Coldman, A.J. (2016). Correlates of women’s intentions to be screened for human papillomavirus for cervical cancer screening with an extended interval. 16(1). https://doi.org/10.1186/s12889-016-2865-8.

Ogilvie, G.S., Mitchell, S., Sekikubo, M., Biryabarema, C., Byamugisha, J., Jeronimo, J., Miller, D., Steinberg, M. and Money, D.M. (2013). Results of a community-based cervical cancer screening pilot project using human papillomavirus self-sampling in Kampala, Uganda. *International Journal of Gynecology & Obstetrics*, 122(2), pp.118–123. https://doi.org/10.1016/j.ijgo.2013.03.019.

Ojidu, H., Palmer, H., Lewandowski, J., Hampton, J., Blakeborough, T., Epstein, O. and McAlindon, M.E. (2018). Patient tolerance and acceptance of different colonic imaging modalities. *European Journal of Gastroenterology & Hepatology*, 30(5), pp.520–525. https://doi.org/10.1097/meg.0000000000001090.

Oketch, S.Y., Kwena, Z., Choi, Y., Adewumi, K., Moghadassi, M., Bukusi, E.A. and Huchko, M.J. (2019). Perspectives of women participating in a cervical cancer screening campaign with community-based HPV self-sampling in rural western Kenya: a qualitative study. *BMC Women’s Health*, [online] 19(1). https://doi.org/10.1186/s12905-019-0778-2.

Ong, J.J., Temple-Smith, M., Chen, M., Walker, S., Grulich, A. and Fairley, C.K. (2014). Exploring anal self-examination as a means of screening for anal cancer in HIV positive men who have sex with men: a qualitative study. *BMC Public Health*, 14(1). https://doi.org/10.1186/1471-2458-14-1257.

Opito, R., Tiyo Ayikobua, E., Akurut, H., Alwedo, S., Ssentongo, S.M., Erabu, W.D., Oucul, L., Kirya, M., Bukenya, L.L., Ekwamu, E., Oluka, A.I., Kabwigu, S., Othieno, E. and Mwaka, A.D. (2025). Factors associated with uptake and acceptability of cervical cancer screening among female sex workers in Northeastern Uganda: A cross-sectional study. *PLOS ONE*, 20(1), p.e0312988. https://doi.org/10.1371/journal.pone.0312988.

Oranratanaphan, S., Termrungruanglert, W. and Khemapech, N. (2014). Acceptability of Self-Sampling HPV Testing Among Thai Women for Cervical Cancer Screening. *Asian Pacific Journal of Cancer Prevention*, 15(17), pp.7437–7441. https://doi.org/10.7314/apjcp.2014.15.17.7437.

Ørnskov, D., Jochumsen, K., Steiner, P.H., Grunnet, I.M., Lykkebo, A.W. and Waldstrøm, M. (2021). Clinical performance and acceptability of self-collected vaginal and urine samples compared with clinician-taken cervical samples for HPV testing among women referred for colposcopy. A cross-sectional study. *BMJ Open*, 11(3), p.e041512. https://doi.org/10.1136/bmjopen-2020-041512.

Ortiz, A.P., Alejandro, N., Pérez, C.M., Otero, Y., Marievelisse Soto-Salgado, Palefsky, J.M., Tortolero-Luna, G. and Romaguera, J. (2012). Acceptability of cervical and anal HPV self-sampling in a sample of Hispanic women in Puerto Rico. *PubMed*.

Osborne, J.M., Flight, I., Wilson, C.J., Chen, G., Ratcliffe, J. and Young, G.P. (2018). The impact of sample type and procedural attributes on relative acceptability of different colorectal cancer screening regimens. *Patient preference and adherence*, [online] 12, pp.1825–1836. https://doi.org/10.2147/PPA.S172143.

Pan, X.-F., Zhao, Z.-M., Sun, J., Chen, F., Wen, Q.-L., Liu, K., Song, G.-Q., Zhang, J.-J., Wen, Y., Fu, C.-J. and Yang, C.-X. (2014). Acceptability and Correlates of Primary and Secondary Prevention of Cervical Cancer among Medical Students in Southwest China: Implications for Cancer Education. *PLoS ONE*, 9(10), p.e110353. https://doi.org/10.1371/journal.pone.0110353.

Pantano, N. de P., Fregnani, J.H., Resende, J.C., Zeferino, L.C., Fonseca, B. de O., Antoniazzi, M., Oliveira, C.M. de, Sant’ana, G. da R. and Longatto-Filho, A. (2020). Evaluation of human papillomavirus self-collection offered by community health workers at home visits among under-screened women in Brazil. *Journal of Medical Screening*, 28(2), pp.163–168. https://doi.org/10.1177/0969141320941056.

Parker, S.L., Amboree, T.L., Bulsara, S., Daheri, M., Anderson, M.L., Hilsenbeck, S.G., Jibaja-Weiss, M.L., Zare, M., Schmeler, K.M., Deshmukh, A.A., Chiao, E.Y., Scheurer, M.E. and Montealegre, J.R. (2023). Self-Sampling for Human Papillomavirus Testing: Acceptability in a U.S. Safety Net Health System. *American Journal of Preventive Medicine*, [online] 66(3), pp.540–547. <https://doi.org/10.1016/j.amepre.2023.10.020>.

Passamonti, B., Malaspina, M., Fraser, C. G., Tintori, B., Carlani, A., D'Angelo, V., Galeazzi, P., Di Dato, E., Mariotti, L., Bulletti, S., D'Amico, M. R., Gustinucci, D., Martinelli, N., Spita, N., Cesarini, E., Rubeca, T., Giaimo, M., Segnan, N., & Senore, C. (2018). A comparative effectiveness trial of two faecal immunochemical tests for haemoglobin (FIT). Assessment of test performance and adherence in a single round of a population-based screening programme for colorectal cancer. *Gut*, *67*(3), 485–496. <https://doi.org/10.1136/gutjnl-2016-312716>

Patel, D., Akporobaro, A., Chinyanganya, N., Hackshaw, A., Seale, C., Spiro, S.G. and Griffiths, C. (2011). Attitudes to participation in a lung cancer screening trial: a qualitative study. *Thorax*, 67(5), pp.418–425. https://doi.org/10.1136/thoraxjnl-2011-200055.

Patel, H., Moss, E.L. and Sherman, S.M. (2018). HPV primary cervical screening in England: Women’s awareness and attitudes. *Psycho-Oncology*, 27(6), pp.1559–1564. https://doi.org/10.1002/pon.4694.

Peery, A.F., Hoppo, T., Garman, K.S., Dellon, E.S., Daugherty, N., Bream, S., Sanz, A.F., Davison, J., Spacek, M., Connors, D., Faulx, A.L., Chak, A., Luketich, J.D., Shaheen, N.J. and Jobe, B.A. (2012). Feasibility, safety, acceptability, and yield of office-based, screening transnasal esophagoscopy (with video). *Gastrointestinal Endoscopy*, 75(5), pp.945-953.e2. https://doi.org/10.1016/j.gie.2012.01.021.

Peitzmeier, S.M., Agénor, M., Bernstein, I.M., McDowell, M., Alizaga, N.M., Reisner, S.L., Pardee, D.J. and Potter, J. (2017). ‘It Can Promote an Existential Crisis’: Factors Influencing Pap Test Acceptability and Utilization Among Transmasculine Individuals. *Qualitative Health Research*, 27(14), pp.2138–2149. https://doi.org/10.1177/1049732317725513.

Penaranda, E., Molokwu, J., Flores, S., Byrd, T., Brown, L. and Shokar, N. (2015). Women’s Attitudes Toward Cervicovaginal Self-Sampling for High-Risk HPV Infection on the US-Mexico Border. *Journal of Lower Genital Tract Disease*, 19(4), pp.323–328. https://doi.org/10.1097/lgt.0000000000000134.

Peremiquel‐Trillas, P., Martínez, J.M., Sònia Paytubi, Frias‐Gomez, J., Pelegrina, B., Marin, F., Benavente, Y., Ibáñez, R., Barahona, M., Sergi Fernandez‐Gonzalez, Cárdenas, L., Taltavull, A., Darder, E., Sanjosé, S. de, Widschwendter, M., Matias‐Guiu, X., Bosch, X., Pineda, M., Ponce, J. and Brunet, J. (2025). Acceptability and somatic mutations in cervicovaginal self‐sampling for early endometrial cancer detection in women with Lynch syndrome. *International Journal of Cancer*. https://doi.org/10.1002/ijc.35368.

Perera, K.C., Mapitigama, K.N. and Abeysena, T.C. (2022). Acceptability, simplicity, and relevance of the new human papillomavirus/DNA test among 35-year-old ever-married women in a district of Sri Lanka: focus group discussions. *BMC Women s Health*, 22(1). https://doi.org/10.1186/s12905-022-01712-2.

Petrik, A.F., Rivelli, J.S., Firemark, A.J., Johnson, C.A., Locher, B.W., Gille, S., Najarian, M.J., Varga, A.M., Schneider, J.L., Green, B. and Winer, R.L. (2024). A qualitative assessment of the acceptability of human papillomavirus self-sampling and informational materials among diverse populations. *Cancer medicine*, [online] 13(14), p.e70033. https://doi.org/10.1002/cam4.70033.

Pham, R., Cross, S., Fernandez, B., Corson, K., Dillon, K., Yackley, C. and Davis, M.M. (2017). ‘Finding the Right FIT’: Rural Patient Preferences for Fecal Immunochemical Test (FIT) Characteristics. *The Journal of the American Board of Family Medicine*, 30(5), pp.632–644. https://doi.org/10.3122/jabfm.2017.05.170151.

Phoolcharoen, N., Kantathavorn, N., Krisorakun, W., Taepisitpong, C., Krongthong, W. and Saeloo, S. (2018). Acceptability of Self-Sample Human Papillomavirus Testing Among Thai Women Visiting a Colposcopy Clinic. *Journal of Community Health*, [online] 43(3), pp.611–615. https://doi.org/10.1007/s10900-017-0460-2.

Pierz, A.J., Ajeh, R., Fuhngwa, N., Nasah, J., Dzudie, A., Nkeng, R., Anastos, K.M., Castle, P.E. and Adedimeji, A. (2021). Acceptability of Self-Sampling for Cervical Cancer Screening Among Women Living With HIV and HIV-Negative Women in Limbé, Cameroon. *Frontiers in Reproductive Health*, 2. https://doi.org/10.3389/frph.2020.561202.

Ploysawang, P., Pitakkarnkul, S., Kolaka, W., Panwad Ratanasrithong, Uraiwan Khomphaiboonkij, Chonticha Tipmed, Kanda Seeda, Parinda Pangmuang and Suleeporn Sangrajrang (2023). Acceptability and Preference for Human Papilloma Virus Self-Sampling among Thai Women Attending National Cancer Institute. *Asian Pacific journal of cancer prevention*, 24(2), pp.607–612. https://doi.org/10.31557/apjcp.2023.24.2.607.

Podolak, I., Kisia, C., Omosa-Manyonyi, G. and Cosby, J. (2017). Using a multimethod approach to develop implementation strategies for a cervical self-sampling program in Kenya. *BMC Health Services Research*, 17(1). https://doi.org/10.1186/s12913-017-2160-0.

Pourette, D., Cripps, A., Guerrien, M., Desprès, C., Opigez, E., Bardou, M. and Dumont, A. (2022). Assessing the Acceptability of Home-Based HPV Self-Sampling: A Qualitative Study on Cervical Cancer Screening Conducted in Reunion Island Prior to the RESISTE Trial. *Cancers*, 14(6), p.1380. https://doi.org/10.3390/cancers14061380.

Proctor, L., Grennan, T., Albert, A., Miller, D., Sadownik, L. and Lee, M. (2019). Screening for Anal Cancer in Women With a History of Vulvar High-Grade Squamous Intraepithelial Lesions. *Journal of Lower Genital Tract Disease*, 23(4), pp.265–271. https://doi.org/10.1097/lgt.0000000000000490.

Qu, S.-X., Ni, Y.-H., Qin, J.-F., Chen, X.-Y., Wu, W.-L. and Zhang, W.-C. (2023). Experience and acceptability for HPV self-sampling among women in Jiangsu province, China: a cross-sectional survey. *Journal of Obstetrics and Gynaecology: The Journal of the Institute of Obstetrics and Gynaecology*, [online] 43(1), p.2204942. https://doi.org/10.1080/01443615.2023.2204942.

Racey, C.S. and Gesink, D.C. (2015). Barriers and Facilitators to Cervical Cancer Screening Among Women in Rural Ontario, Canada: The Role of Self-Collected HPV Testing. *The Journal of Rural Health*, 32(2), pp.136–145. https://doi.org/10.1111/jrh.12136.

Racey, C.S., Gesink, D.C., Burchell, A.N., Trivers, S., Wong, T. and Rebbapragada, A. (2016). Randomized Intervention of Self-Collected Sampling for Human Papillomavirus Testing in Under-Screened Rural Women: Uptake of Screening and Acceptability. *Journal of women’s health (2002)*, [online] 25(5), pp.489–97. https://doi.org/10.1089/jwh.2015.5348.

Rainey, L., Jervaeus, A., Donnelly, L.S., Evans, D.G., Hammarström, M., Hall, P., Wengström, Y., Broeders, M.J.M. and Waal, D. (2019). Women’s perceptions of personalized risk‐based breast cancer screening and prevention: An international focus group study. *Psycho-Oncology*, 28(5), pp.1056–1062. https://doi.org/10.1002/pon.5051.

Rainey, L., van der Waal, D., & Broeders, M. J. M. (2020). Dutch women's intended participation in a risk-based breast cancer screening and prevention programme: a survey study identifying preferences, facilitators and barriers. *BMC cancer*, *20*(1), 965. <https://doi.org/10.1186/s12885-020-07464-2>

Rainey, L., van der Waal, D., Donnelly, L.S., Evans, D.G., Wengström, Y. and Broeders, M. (2018). Women’s decision-making regarding risk-stratified breast cancer screening and prevention from the perspective of international healthcare professionals. *PLOS ONE*, 13(6), p.e0197772. <https://doi.org/10.1371/journal.pone.0197772>.

Rana, S.K., Perkins, R.B., Carr, D., Feldman, S., Welch, K., Duffey-Lind, E. and Villa, A. (2024). Evaluation of Human Papillomavirus Vaccination and Cancer Prevention Behaviors among LGBTQI + Individuals: A Cross-Sectional Study. *Journal of Community Health*. https://doi.org/10.1007/s10900-024-01401-1.

Rawat, A., Sanders, C., Mithani, N., Amuge, C., Pedersen, H., Namugosa, R., Payne, B., Mitchell‐Foster, S., Orem, J., Ogilvie, G. and Nakisige, C. (2020). Acceptability and preferences for self‐collected screening for cervical cancer within health systems in rural Uganda: A mixed‐methods approach. *International Journal of Gynecology & Obstetrics*, 152(1), pp.103–111. https://doi.org/10.1002/ijgo.13454.

Raz, D.J., Nehoray, B., Ceniceros, A., Motarjem, P., Landau, S., Nelson, R.A. and Gray, S.W. (2025). Feasibility of a cancer screening program using multicancer early detection testing and whole‐body magnetic resonance imaging in a high‐risk population. *Cancer*, 131(1). https://doi.org/10.1002/cncr.35709.

Read, T., Vodstrcil, L., Grulich, A., Farmer, C., Bradshaw, C., Chen, M., Tabrizi, S., Hocking, J., Anderson, J. and Fairley, C. (2013). Acceptability of digital anal cancer screening examinations in HIV-positive homosexual men. *HIV Medicine*, 14(8), pp.491–496. https://doi.org/10.1111/hiv.12035.

Redwood, D.G., Blake, I.D., Provost, E.M., Kisiel, J.B., Sacco, F.D. and Ahlquist, D.A. (2019). Alaska Native Patient and Provider Perspectives on the Multitarget Stool DNA Test Compared With Colonoscopy for Colorectal Cancer Screening. *Journal of Primary Care & Community Health*, 10, p.215013271988429. <https://doi.org/10.1177/2150132719884295>.

Reisner, S. L., Deutsch, M. B., Peitzmeier, S. M., White Hughto, J. M., Cavanaugh, T. P., Pardee, D. J., McLean, S. A., Panther, L. A., Gelman, M., Mimiaga, M. J., & Potter, J. E. (2018). Test performance and acceptability of self- versus provider-collected swabs for high-risk HPV DNA testing in female-to-male trans masculine patients. *PloS one*, *13*(3), e0190172. <https://doi.org/10.1371/journal.pone.0190172>

Reiter, P.L. and McRee, A.-L. (2014). Cervical cancer screening (Pap testing) behaviours and acceptability of human papillomavirus self-testing among lesbian and bisexual women aged 21–26 years in the USA. *Journal of Family Planning and Reproductive Health Care*, [online] 41(4), pp.259–264. https://doi.org/10.1136/jfprhc-2014-101004.

Remmel, M.-L., Kadri Suija, Riina Raudne, Tisler, A., Anda Ķīvīte-Urtāne, Mindaugas Stankūnas, Nygård, M., Gunvor Aasbø, Maļina, L. and Anneli Uusküla (2024). Women’s perspectives on the acceptability of risk-based cervical cancer screening. *BMC Cancer*, 24(1). https://doi.org/10.1186/s12885-024-13050-7.

Resong, P.J., Niu, J., Duhon, G.F., Foxhall, L.E., Shete, S., Volk, R.J. and Toumazis, I. (2024). Acceptability of Personalized Lung Cancer Screening Program Among Primary Care Providers. *Cancer Prevention Research*, 17(2), pp.51–57. https://doi.org/10.1158/1940-6207.capr-23-0359.

Rezq, K.A., Algamdi, M.M., Rehab Alanazi, Sarah Hatab Alanazi, Fatmah Alhujairy, Radwa Albalawi and Wafa Al-Zamaa (2023). Knowledge, Perception, and Acceptance of HPV Vaccination and Screening for Cervical Cancer among Saudi Females: A Cross-Sectional Study. *Vaccines*, 11(7), pp.1188–1188. https://doi.org/10.3390/vaccines11071188.

Ridgeway, J.L., Albertie, M.L., Williams, S., Pantoja, E., Stephenson, N., Pacheco-Spann, L., Ball, C.T., Breitkopf, C.R., Bakkum-Gamez, J.N., Chase, L., DeStephano, C.C. and Sherman, M.E. (2021). Development of novel home-based tampon sampling for endometrial cancer: findings from community-based focus groups with Black and White women. *Journal of Clinical and Translational Science*, 5(1). https://doi.org/10.1017/cts.2021.787.

Rodrigues, L.L.S., Morgado, M.G., Sahasrabuddhe, V.V., De Paula, V.S., Oliveira, N.S., Chavez-Juan, E., Da Silva, D.M., Kast, W.M., Nicol, A.F. and Pilotto, J.H. (2018). Cervico-vaginal self-collection in HIV-infected and uninfected women from Tapajós region, Amazon, Brazil: High acceptability, hrHPV diversity and risk factors. *Gynecologic Oncology*, 151(1), pp.102–110. https://doi.org/10.1016/j.ygyno.2018.08.004.

Rodriguez, N.M., Brennan, L.P., Claure, L., Balian, L.N., Champion, V.L. and Forman, M.R. (2023). Leveraging COVID-era innovation for cervical cancer screening: Clinician awareness and attitudes toward self-sampling and rapid testing for HPV detection. *PloS One*, [online] 18(3), p.e0282853. https://doi.org/10.1371/journal.pone.0282853.

Rodriguez, S.A., Higashi, R.T., Betts, A.C., Ortiz, C., Tiro, J.A., Luque, A.E. and Barnes, A. (2020). Anal Cancer and Anal Cancer Screening Knowledge, Attitudes, and Perceived Risk Among Women Living With HIV. *Journal of Lower Genital Tract Disease*, 25(1), pp.43–47. https://doi.org/10.1097/lgt.0000000000000578.

Rohner, E., McGuire, F.H., Liu, Y., Li, Q., Miele, K., Desai, S.A., Schmitt, J.W., Knittel, A., Nelson, J.A.E., Edelman, C., Sivaraman, V., Baker, A., Romocki, L.S., Rahangdale, L. and Smith, J.S. (2020). Racial and Ethnic Differences in Acceptability of Urine and Cervico-Vaginal Sample Self-Collection for HPV-Based Cervical Cancer Screening. *Journal of Women’s Health*, 29(7), pp.971–979. https://doi.org/10.1089/jwh.2019.8132.

Rosenbaum, A.J., Gage, J.C., Alfaro, K.M., Ditzian, L.R., Maza, M., Scarinci, I.C., Felix, J.C., Castle, P.E., Villalta, S., Miranda, E. and Cremer, M.L. (2014). Acceptability of self-collected versus provider-collected sampling for HPV DNA testing among women in rural El Salvador. *International Journal of Gynecology & Obstetrics*, 126(2), pp.156–160. https://doi.org/10.1016/j.ijgo.2014.02.026.

Ross, M.W., Bennis, S.L., Zoschke, N., Rosser, B.R.S., Stull, C.L., Nyitray, A.G., Khariwala, S.S., Nichols, M., Flash, C. and Wilkerson, M. (2023). Screening for HPV-Related Oropharyngeal Cancer in Gay and Bisexual Men: Acceptability and Predicting Possible Use of ‘Oral Selfies’ by Smartphone as a Secondary Prevention Approach. *Venereology*, [online] 2(4), pp.180–193. https://doi.org/10.3390/venereology2040016.

Rosser, J.I., Njoroge, B. and Huchko, M.J. (2015). Changing knowledge, attitudes, and behaviors regarding cervical cancer screening: The effects of an educational intervention in rural Kenya. *Patient Education and Counseling*, 98(7), pp.884–889. https://doi.org/10.1016/j.pec.2015.03.017.

Roux, A.N., Kenfack, B., Ndjalla, A., Sormani, J., Wisniak, A., Tatrai, K., Vassilakos, P., Petignat, P. and Schmidt, N. (2021). Barriers to cervical cancer prevention in rural Cameroon: a qualitative study on healthcare providers’ perspective. *BMJ Open*, [online] 11(6), p.e043637. https://doi.org/10.1136/bmjopen-2020-043637.

Ruge, A., Lee, C. and Brown, W.J. (1995). Inpatient cervical screening: a survey of patient acceptability. *Australian Journal of Public Health*, 19(1), pp.96–97. https://doi.org/10.1111/j.1753-6405.1995.tb00306.x.

Sachdeva, M., Datchoua, A.M., Virginie Flore Yakam, Kenfack, B., Jonnalagedda-Cattin, M., Jean-Philippe Thiran, Petignat, P. and Schmidt, N.C. (2024). Acceptability of artificial intelligence for cervical cancer screening in Dschang, Cameroon: a qualitative study on patient perspectives. *Reproductive Health*, 21(1). https://doi.org/10.1186/s12978-024-01828-8.

Saengow, U., Chongsuwiwatvong, V., Geater, A. and Birch, S. (2015). Preferences and Acceptance of Colorectal Cancer Screening in Thailand. *Asian Pacific Journal of Cancer Prevention*, 16(6), pp.2269–2276. https://doi.org/10.7314/apjcp.2015.16.6.2269.

Samimi, G., Temkin, Sarah M., Weil, Carol J., Han, Paul K. J., LeeVan, E., Rubinstein, Wendy S., Swigart, Tessa M., Caban, S., Dent, K. and Minasian, Lori M. (2024). Perceptions of Multicancer Detection Tests Among Primary Care Physicians and Laypersons: A Qualitative Study. *Cancer Medicine*, 13(21). https://doi.org/10.1002/cam4.70281.

Sanchaisuriya, P., Pengsaa, P., Sriamporn, S., Schelp, F.P., Onanong Kritpetcharat, Krittika Suwanrungruang, Wongsa Laohasiriwong, Noda, S. and Kato, S. (2004). Experience with a self-administered device for cervical cancer screening by Thai women with different educational backgrounds. *PubMed*, 5(2), pp.144–50.

Sangster, J.F., Gerace, T.M. and Bass, M.J. (1986). Acceptability of a Faecal Occult Blood Screening Protocol for Carcinoma of the Colon in Family Practice. *Family Practice*, 3(4), pp.246–250. https://doi.org/10.1093/fampra/3.4.246.

Sankaranarayanan, R., Rajkumar, R., Esmy, P.O., Fayette, J.M., Shanthakumary, S., Frappart, L., Thara, S. and Cherian, J. (2007). Effectiveness, safety and acceptability of ‘see and treat’ with cryotherapy by nurses in a cervical screening study in India. *British Journal of Cancer*, 96(5), pp.738–743. https://doi.org/10.1038/sj.bjc.6603633.

Sargent, A., Fletcher, S., Bray, K., Kitchener, H.C. and Crosbie, E.J. (2019). Cross-sectional study of HPV testing in self-sampled urine and comparison with matched vaginal and cervical samples in women attending colposcopy for the management of abnormal cervical screening. *BMJ Open*, 9(4), p.e025388. https://doi.org/10.1136/bmjopen-2018-025388.

Sauvageau, C., Duval, B., Gilca, V., Lavoie, F. and Ouakki, M. (2007). Human Papilloma Virus vaccine and cervical cancer screening acceptability among adults in Quebec, Canada. *BMC Public Health*, 7(1). https://doi.org/10.1186/1471-2458-7-304.

Saville, M., Hawkes, D., Mclachlan, E., Anderson, S. and Arabena, K. (2018). Self-collection for under-screened women in a National Cervical Screening Program: pilot study. *Current Oncology*, 25(1), p.27. https://doi.org/10.3747/co.25.3915.

Scarinci, I.C., Litton, A.G., Garcés-Palacio, I.C., Partridge, E.E. and Castle, P.E. (2013). Acceptability and Usability of Self-Collected Sampling for HPV Testing Among African-American Women Living in the Mississippi Delta. *Women’s Health Issues*, 23(2), pp.e123–e130. https://doi.org/10.1016/j.whi.2012.12.003.

Scepanovic, M., Jovanovic, O., Keber, D., Jovanovic, I., Dragan Miljus, Nikolic, G., Kovacevic, B., Pavlovic, A., Predrag Dugalic, Aleksandar Nagorni, Sasa Grgov, Knezevic, S., Milenkovic, P., Perisic, N. and Faivre, J. (2016). Faecal occult blood screening for colorectal cancer in Serbia: a pilot study. *European Journal of Cancer Prevention*, 26(3), pp.195–200. https://doi.org/10.1097/cej.0000000000000247.

Schliemann, D., Ramanathan, K., Nor, Ciarán O’Neill, Cardwell, C., Ismail, R., Kassim, Z., Kee, F., Tin Tin Su and Donnelly, M. (2023). Implementation of a home-based colorectal cancer screening intervention in Malaysia (CRC-SIM). *BMC Cancer*, 23(1). https://doi.org/10.1186/s12885-022-10487-6.

Schmeising-Barnes, N., Waller, J. and Marlow (2024). Attitudes to multi-cancer early detection (MCED) blood tests for population-based screening: A qualitative study in Great Britain. *Social science & medicine*, pp.116762–116762. https://doi.org/10.1016/j.socscimed.2024.116762.

Schofield, A.M., Sadler, L., Nelson, L., Gittins, M., Desai, M., Sargent, A., McMahon, R.F.T., Hill, J., Crosbie, E.J., Patnick, J. and Kitchener, H.C. (2016). A prospective study of anal cancer screening in HIV-positive and negative MSM. *AIDS (London, England)*, [online] 30(9), pp.1375–1383. https://doi.org/10.1097/QAD.0000000000001045.

Seamount, F., Sauvaget, C. and Dangbemey, D.P. (2023). Overcoming barriers to cancer screening in resource-constrained settings: Care4Afrique: a pilot cervical cancer screening programme in three African countries. [online] https://doi.org/10.1200/go.22.00051.

Sechi, I., Cocuzza, C., Martinelli, M., Muresu, N., Castriciano, S., Sotgiu, G. and Piana, A. (2022). Comparison of Different Self-Sampling Devices for Molecular Detection of Human Papillomavirus (HPV) and Other Sexually Transmitted Infections (STIs): A Pilot Study. *Healthcare*, 10(3), p.459. https://doi.org/10.3390/healthcare10030459.

Sellors, J.W., Lorincz, A.T., Mahony, J.B., Iwona Mielzynska, Lytwyn, A., Roth, P., Howard, M., Chong, S., Daya, D., Chapman, W. and Chernesky, M. (2000). Comparison of self-collected vaginal, vulvar and urine samples with physician-collected cervical samples for human papillomavirus testing to detect high-grade squamous intraepithelial lesions. *PubMed*, 163(5), pp.513–8.

Selmouni, F., Amrani, L., Sauvaget, C., Bakkar, M., El Khannoussi, B., Souadka, A., Benkabbou, A., Majbar, M.A., Belekhel, L., Lucas, E., Muwonge, R., Chami Khazraji, Y., Mohsine, R., Bennani, M., Sankaranarayanan, R., Bekkali, R. and Basu, P. (2022). Delivering colorectal cancer screening integrated with primary health care services in Morocco: Lessons learned from a demonstration project. *Cancer*, 128(6), pp.1219–1229. https://doi.org/10.1002/cncr.34061.

Selmouni, F., Sauvaget, C., Dangbemey, D. P., Kpebo, D. D. O., Dieng, N. M., Lucas, E., ... & Basu, P. (2022). Lessons learnt from pilot cervical cancer screening and treatment programmes integrated to routine primary health care services in Benin, Cote d'Ivoire, and Senegal. *JCO Global Oncology*, *8*, e2200051. doi: <https://doi.org/10.1200/go.22.00051>

Senore, C., Correale, L., Regge, D., Hassan, C., Iussich, G., Silvani, M., Arrigoni, A., Morra, L. and Segnan, N. (2018). Flexible Sigmoidoscopy and CT Colonography Screening: Patients’ Experience with and Factors for Undergoing Screening—Insight from the Proteus Colon Trial. *Radiology*, 286(3), pp.873–883. https://doi.org/10.1148/radiol.2017170228.

Senore, C., Ederle, A., Fantin, A., Andreoni, B., Bisanti, L., Grazzini, G., Zappa, M., Ferrero, F., Marutti, A., Giuliani, O., Armaroli, P. and Segnan, N. (2011). Acceptability and side-effects of colonoscopy and sigmoidoscopy in a screening setting. *Journal of Medical Screening*, 18(3), pp.128–134. https://doi.org/10.1258/jms.2011.010135.

Senore, C., Segnan, N., Rossini, F.P., Ferraris, R., Cavallero, M., Coppola, F., Pennazio, M. and Atkin, W.S. (1996). Screening for Colorectal Cancer by Once Only Sigmoidoscopy: A Feasibility Study in Turin, Italy. *Journal of Medical Screening*, 3(2), pp.72–78. <https://doi.org/10.1177/096914139600300205>.

Seow, A., Straughan, P. T., Ng, E. H., Emmanuel, S. C., Tan, C. H., & Lee, H. P. (1998). Population-based mammographic screening in Singapore: what are participants' views?. *Annals of the Academy of Medicine, Singapore*, *27*(2), 154–160.

Shaheen, N.J., Komanduri, S., Muthusamy, V.R., Wani, S., O’Donovan, M., Kaushal, R. and Haydek, J.M. (2021). Acceptability and Adequacy of a Non-endoscopic Cell Collection Device for Diagnosis of Barrett’s Esophagus: Lessons Learned. *Digestive Diseases and Sciences*, 67(1), pp.177–186. https://doi.org/10.1007/s10620-021-06833-6.

Sherman, S.M., Brewer, N., Bartholomew, K., Bromhead, C., Crengle, S., Cunningham, C., Jeroen Douwes, Sunia Foliaki, Grant, J., Maxwell, A., McPherson, G., Scott, N., Wihongi, H. and Potter, J.D. (2022). Human papillomavirus self‐testing among unscreened and under‐screened Māori, Pasifika and Asian women in Aotearoa New Zealand: A preference survey among responders and interviews with clinical‐trial nonresponders. *Health Expectations*, [online] 25(6), pp.2914–2923. https://doi.org/10.1111/hex.13599.

Shrestha, S., Thapa, S., Sims, P., Ardelean, A., Basu, A., Caws, M., Gurung, S.C. and Holdsworth, G. (2021). Feasibility of HPV self-sampling pathway in Kathmandu Valley, Nepal using a human-centred design approach. *Sexual and Reproductive Health Matters*, 29(3). https://doi.org/10.1080/26410397.2021.2005283.

Sierra, M.A., Wheeler, J.C.W., Devereux, L., Trainer, A.H. and Keogh, L. (2021). Exploring Implementation of Personal Breast Cancer Risk Assessments. *Journal of Personalized Medicine*, 11(10), p.992. https://doi.org/10.3390/jpm11100992.

Smit, A.K., Reyes-Marcelino, G., Keogh, L., Dunlop, K., Newson, A.J. and Cust, A.E. (2020). Implementation considerations for offering personal genomic risk information to the public: a qualitative study. *BMC Public Health*, 20(1). https://doi.org/10.1186/s12889-020-09143-0.

Smith, J.A., Des, A.C., Deal, A.M., Richman, A.R., Perez-Heydrich, C., Yen-Lieberman, B., Barclay, L., Belinson, J.L., Rinas, A.C. and Brewer, N.T. (2018). Mailed Human Papillomavirus Self-Collection With Papanicolaou Test Referral for Infrequently Screened Women in the United States. 45(1), pp.42–48. https://doi.org/10.1097/olq.0000000000000681.

Smith, L., Racey, C.S., Gondara, L., Krajden, M., Lee, M., Ruth Elwood Martin, Stuart, G., Peacock, S., Coldman, A.J., Franco, E.L., Dirk van Niekerk and Ogilvie, G. (2021). Women’s acceptability of and experience with primary human papillomavirus testing for cervix screening: HPV FOCAL trial cross-sectional online survey results. *BMJ Open*, 11(10), pp.e052084–e052084. https://doi.org/10.1136/bmjopen-2021-052084.

Smith, L.W., Booth, A., Racey, C.S., Smith, B., Prabhakaran, A., Smritee Dabee, Hong, Q., Niazi, N. and Ogilvie, G.S. (2024). Self-Screening for Cervical Cancer Offered through a Digital Platform in a Region of British Columbia with Lower Screening Rates. *Current Oncology*, 31(9), pp.5399–5411. https://doi.org/10.3390/curroncol31090399.

Sokale, I.O., Kuo, D.C., Hoppenot, C.M., Reitzel, L.R., Juarez, L.H., Hernandez, K.J., Parker, S.L., Amos, C., Daheri, M., Keene, K.R., Montealegre, J.R. and Thrift, A.P. (2024). Self-Collection for Primary HPV Testing: Acceptability in a Large Urban Emergency Department. *Annals of Emergency Medicine*. https://doi.org/10.1016/j.annemergmed.2024.10.002.

Song, J., Ni, Y.-H., Fang, J., Qu, S.-X., Chen, X.-Y., Wu, W.-L., Zhang, W.-C. and Qin, J.-F. (2024). The levels of women’s awareness, experience, acceptability and preference for Vaginal Human Papillomavirus (HPV) self-sampling in three provinces of China: a cross-sectional study. *BMC women’s health*, [online] 24(1), p.343. https://doi.org/10.1186/s12905-024-03186-w.

Sormani, J., Kenfack, B., Wisniak, A., Moukam Datchoua, A., Lemoupa Makajio, S., Schmidt, N.C., Vassilakos, P. and Petignat, P. (2021). Exploring Factors Associated with Patients Who Prefer Clinician-Sampling to HPV Self-Sampling: A Study Conducted in a Low-Resource Setting. *International Journal of Environmental Research and Public Health*, [online] 19(1), p.54. https://doi.org/10.3390/ijerph19010054.

Sossauer, G., Zbinden, M., Tebeu, P.-M., Fosso, G.K., Untiet, S., Vassilakos, P. and Petignat, P. (2014). Impact of an Educational Intervention on Women’s Knowledge and Acceptability of Human Papillomavirus Self-Sampling: A Randomized Controlled Trial in Cameroon. *PLoS ONE*, 9(10), p.e109788. https://doi.org/10.1371/journal.pone.0109788.

Sowah, L.A., Buchwald, U.K., Riedel, D.J., Gilliam, B.L., Khambaty, M., Fantry, L., Spencer, D.E., Weaver, J., Taylor, G., Skoglund, M., Amoroso, A. and Redfield, R.R. (2015). Anal Cancer Screening in an Urban HIV Clinic: Provider Perceptions and Practice. *Journal of the International Association of Providers of AIDS Care*, 14(6), pp.497–504. https://doi.org/10.1177/2325957415601504.

Stoltzfus, K.C., Popalis, M.L., Reiter, P.L. and Moss, J.L. (2021). Perspectives on self‐sampling for cancer screening among rural and urban women: Multilevel factors related to acceptability. *The Journal of Rural Health*. https://doi.org/10.1111/jrh.12590.

Sudhakaran, S., Bhatla, N., Mathur, S.R., Mahey, R., Vashist, S., Natarajan, J., Garima Kachhawa, Kumari, R. And Pranay Tanwar (2023). Evaluation of cervical cancer screening during pregnancy in India: Human papillomavirus testing can change the paradigm. *National medical journal of India*, 36, pp.17–21. https://doi.org/10.25259/nmji_768_20.

Sultana, F., Mullins, R., Murphy, M., English, D.R., Simpson, J.A., Drennan, K.T., Heley, S., Wrede, C.D., Brotherton, J.M.L., Saville, M. and Gertig, D.M. (2015). Women’s views on human papillomavirus self-sampling: focus groups to assess acceptability, invitation letters and a test kit in the Australian setting. *Sexual Health*, 12(4), p.279. https://doi.org/10.1071/sh14236.

Sy, A.U., Hernandez, B.Y., Tareg, A., Reichhardt, M. and Buenconsejo-Lum, L. (2017). Acceptability and feasibility of a community based participatory research project comparing cytology and urine HPV DNA testing for cervical cancer screening in Yap, Federated States of Micronesia. *Cancer epidemiology*, [online] 50(Pt B), pp.283–288. https://doi.org/10.1016/j.canep.2017.07.008.

Symmons, S.M., Drury, A. and De Brún, A. (2024). Stakeholder perceptions of cervical screening accessibility and attendance in Ireland: a qualitative study. *Health promotion international*, 39(3). https://doi.org/10.1093/heapro/daae072.

Szarewski, A., Cadman, L., Ashdown-Barr, L. and Waller, J. (2009). Exploring the acceptability of two self-sampling devices for human papillomavirus testing in the cervical screening context: a qualitative study of Muslim women in London. *Journal of Medical Screening*, 16(4), pp.193–198. https://doi.org/10.1258/jms.2009.009069.

Taku, O., Meiring, T.L., Gustavsson, I., Phohlo, K., Garcia-Jardon, M., Mbulawa, Z.Z.A., Businge, C.B., Gyllensten, U. and Williamson, A.-L. (2020). Acceptability of self- collection for human papillomavirus detection in the Eastern Cape, South Africa. *PLOS ONE*, 15(11), p.e0241781. https://doi.org/10.1371/journal.pone.0241781.

Tamalet, C., Ravaux, I., Dhiver, C., Menard, A., Colson, P. and Stein, A. (2016). Feasibility and Acceptability of Anal Self-Sampling for Human Papillomavirus Screening in HIV-Infected Patients. *Intervirology*, 59(2), pp.118–122. https://doi.org/10.1159/000452710.

Tan, W. K., Muldrew, B., Khan, Z., & Fitzgerald, R. C. (2019). A cross-sectional analysis of Facebook comments to study public perception of a new diagnostic test called the cytosponge. *Diseases of the Esophagus*, *32*(1), doy085.

Tassembedo, S., Winter, C.H., Traore, I.T., Adama Ouattara, Mamadou Sawadogo and Meda, N. (2023). Cervical pre-cancer screening by visual inspection of the cervix after application of acetic acid in rural Burkina Faso: evaluation of women’s knowledge, screening practice habits, acceptability and prevalence of risk factors and lesions in Boussé health District. *Pan African Medical Journal*, 45. https://doi.org/10.11604/pamj.2023.45.135.36933.

Taylor, G., McWilliams, L., Woof, V.G., Evans, D.G. and French, D.P. (2022). What are the views of three key stakeholder groups on extending the breast screening interval for low‐risk women? A secondary qualitative analysis. *Health Expectations*, 25(6), pp.3287–3296. https://doi.org/10.1111/hex.13637.

Taylor, L.C., Dennison, R.A., Griffin, S.J., John, S.D., Lansdorp-Vogelaar, I., Thomas, C.V., Thomas, R. and Usher-Smith, J.A. (2023). Implementation of risk stratification within bowel cancer screening: a community jury study exploring public acceptability and communication needs. *BMC Public Health*, [online] 23, p.1798. https://doi.org/10.1186/s12889-023-16704-6.

Taylor, L.C., Dennison, R.A. and Usher-Smith, J.A. (2024). Public acceptability and anticipated uptake of risk-stratified bowel cancer screening in the UK: An online survey. *Preventive Medicine Reports*, [online] 48, p.102927. https://doi.org/10.1016/j.pmedr.2024.102927.

Taylor, T., Williamson, S., Wardle, J., Borrill, J., Sutton, S. and Atkin, W. (2000). Acceptability of flexible sigmoidoscopy screening in older adults in the United Kingdom. *Journal of Medical Screening*, 7(1), pp.38–45. https://doi.org/10.1136/jms.7.1.38.

Thapa, S., Timilsina, A., Bucha, B., Shrestha, S., Safal Kunwar, Raghu Dhital and Holdsworth, G. (2025). Are we ready for self-sampling for cervical cancer screening? Insights from service providers and policy makers in Nepal. *PLOS Global Public Health*, 5(1), pp.e0004114–e0004114. https://doi.org/10.1371/journal.pgph.0004114.

Tonge, J.E., Atack, M., Crosbie, P.A., Barber, P.V., Booton, R. and Colligan, D. (2018). ‘To know or not to know…?’ Push and pull in ever smokers lung screening uptake decision‐making intentions. *Health Expectations*, 22(2), pp.162–172. https://doi.org/10.1111/hex.12838.

Torrado-García, L.M., Martínez-Vega, R. and Bladimiro Rincon-Orozco (2020). A Novel Strategy for Cervical Cancer Prevention Using Cervical-Vaginal Self-Collected Samples Shows High Acceptability in Women Living in Low-Income Conditions from Bucaramanga, Colombia. *International Journal of Women s Health*, Volume 12, pp.1197–1204. https://doi.org/10.2147/ijwh.s265130.

Torres, K.L., Mariño, J.M., Pires Rocha, D.A., de Mello, M.B., de Melo Farah, H.H., Reis, R. dos S., Alves, V. do C.R., Gomes, E., Martins, T.R., Soares, A.C., de Oliveira, C.M. and Levi, J.E. (2018). Self-sampling coupled to the detection of HPV 16 and 18 E6 protein: A promising option for detection of cervical malignancies in remote areas. *PLOS ONE*, 13(7), p.e0201262. https://doi.org/10.1371/journal.pone.0201262.

Tranberg, M., Jensen, J.S., Bech, B.H. and Andersen, B. (2020). Urine collection in cervical cancer screening – analytical comparison of two HPV DNA assays. *BMC Infectious Diseases*, 20(1). https://doi.org/10.1186/s12879-020-05663-7.

Trope, L.A., Chumworathayi, B. and Blumenthal, P.D. (2013). Feasibility of Community-Based careHPV for Cervical Cancer Prevention in Rural Thailand. *Journal of Lower Genital Tract Disease*, 17(3), pp.315–319. https://doi.org/10.1097/lgt.0b013e31826b7b70.

Tsedenbal, B., Enebish, G., Tserensodnom, B. and Saio, M. (2022). Results of Self-Sampling Methodology Impression for Cervical Cancer Screening in Mongolia. *Asian Pacific Journal of Cancer Prevention*, [online] 23(12), pp.4099–4107. https://doi.org/10.31557/apjcp.2022.23.12.4099.

Ugwu, E., Obi, S., Ezechukwu, P., Okafor, I. and Ugwu, A. (2013). Acceptability of human papilloma virus vaccine and cervical cancer screening among female health-care workers in Enugu, Southeast Nigeria. *Nigerian Journal of Clinical Practice*, 16(2), p.249. https://doi.org/10.4103/1119-3077.110141.

Usher-Smith, J.A., Harvey-Kelly, L.L.W., Rossi, S.H., Harrison, H., Griffin, S.J. and Stewart, G.D. (2021). Acceptability and potential impact on uptake of using different risk stratification approaches to determine eligibility for screening: A population-based survey. *Health expectations : an international journal of public participation in health care and health policy*, [online] 24(2), pp.341–351. https://doi.org/10.1111/hex.13175.

Usher-Smith, J.A., Masson, G., Godoy, A., Burge, S.W., Kitt, J., Farquhar, F., Cartledge, J., Kimuli, M., Burbidge, S., Philip, Eckert, C., Hancock, N., Iball, G.R., Rogerson, S., Rossi, S.H., Smith, A., Simmonds, I., Wallace, T., Ward, M. and Matthew (2024). Acceptability of adding a non-contrast abdominal CT scan to screen for kidney cancer and other abdominal pathology within a community-based CT screening programme for lung cancer: A qualitative study. *PLoS ONE*, 19(7), pp.e0300313–e0300313. https://doi.org/10.1371/journal.pone.0300313.

Vahabi, M. and Lofters, A. (2016). Muslim immigrant women’s views on cervical cancer screening and HPV self-sampling in Ontario, Canada. *BMC Public Health*, 16(1). https://doi.org/10.1186/s12889-016-3564-1.

Valdez, D., Cruz, T., Rania, S., Badowski, G., Cassel, K., Wolfgruber, T., Grosskreutz, S., Dulana, L.J., Adonay, R., Gertraud Maskarinec and Shepherd, J.A. (2022). Technical note: Low clinical efficacy, but good acceptability of a point‐of‐care electronic palpation device for breast cancer screening for a lower middle‐income environment. *Medical Physics*, 49(4), pp.2663–2671. https://doi.org/10.1002/mp.15499.

Vanderpool, R.C., Jones, M.G., Stradtman, L.R., Smith, J.S. and Crosby, R.A. (2014). Self-collecting a cervico-vaginal specimen for cervical cancer screening: An exploratory study of acceptability among medically underserved women in rural Appalachia. *Gynecologic Oncology*, 132, pp.S21–S25. https://doi.org/10.1016/j.ygyno.2013.10.008.

Vásquez, C.L., Martínez-Alonso, M., Carles Forné, Jordi Vilaplana, Inés Cruz-Esteve, Sánchez-López, I., Mercé Reñé-Reñé, Cazorla-Sánchez, C., Hernández-Andreu, M., Galindo-Ortego, G., Montserrat Llorens-Gabandé, Pons-Rodríguez, A. and Montserrat Rué (2022). Feasibility and Acceptability of Personalized Breast Cancer Screening (DECIDO Study): A Single-Arm Proof-of-Concept Trial. *International Journal of Environmental Research and Public Health*, 19(16), pp.10426–10426. https://doi.org/10.3390/ijerph191610426.

Virtanen, A., Nieminen, P., Niironen, M., Luostarinen, T. and Anttila, A. (2014). Self-sampling experiences among non-attendees to cervical screening. *Gynecologic Oncology*, 135(3), pp.487–494. https://doi.org/10.1016/j.ygyno.2014.09.019.

Voska, M., Zavoral, M., Grega, T., Majek, O., Martinek, J., Tacheci, I., Benes, M., Vojtechova, G., Drastich, P., Bures, J., Spicak, J., Buckova, B., Ngo, O. and Suchanek, S. (2019). Accuracy of Colon Capsule Endoscopy for Colorectal Neoplasia Detection in Individuals Referred for a Screening Colonoscopy. *Gastroenterology Research and Practice*, 2019, pp.1–8. https://doi.org/10.1155/2019/5975438.

Waller, J., Mccaffery, K., Forrest, S., Szarewski, A., Cadman, L., Austin, J. and Wardle, J. (2006). Acceptability of unsupervised HPV self-sampling using written instructions. *Journal of Medical Screening*, 13(4), pp.208–213. https://doi.org/10.1177/096914130601300409.

Wang, R., Lee, K., Gaydos, C.A., Anderson, J., Keller, J. and Coleman, J. (2020). Performance and acceptability of self-collected human papillomavirus testing among women living with HIV. *International Journal of Infectious Diseases*, 99, pp.452–457. https://doi.org/10.1016/j.ijid.2020.07.047.

Ward, J. E., Boyle, K., Redman, S., & Sanson-Fisher, R. W. (1991). Increasing women’s compliance with opportunistic cervical cancer screening: a randomized trial. *American Journal of Preventive Medicine*, *7*(5), 285-291.

Wassie, M.M., Dix, M., Laven-Law, G., Bulamu, N., Cock, C., Bampton, P., Fraser, R.J., Winter, J.M., Young, G.P. and Symonds, E.L. (2024). Accuracy, Acceptability, and Application: Fecal Immunochemical Tests for Early Detection of Advanced Neoplasia in Colonoscopy-Based Surveillance. *Digestive Diseases and Sciences*, 69(7), pp.2621–2630. https://doi.org/10.1007/s10620-024-08466-x.

Webb, S., Mat Ali, N., Sawyer, A., Clark, D.J., Brown, M.A., Augustin, Y., Woo, Y.L., Khoo, S.P., Hargreaves, S., Staines, H.M., Krishna, S. and Hayes, K. (2024). Patient preference and acceptability of self-sampling for cervical screening in colposcopy clinic attenders: A cross-sectional semi-structured survey. *PLOS global public health*, [online] 4(5), p.e0003186. https://doi.org/10.1371/journal.pgph.0003186.

Weissfeld, J.L., Ling, B.S., Schoen, R.E., Bresalier, R.S., Riley, T. and Prorok, P.C. (2002). Adherence to repeat screening flexible sigmoidoscopy in the Prostate, Lung, Colorectal, and Ovarian (PLCO) Cancer Screening Trial. *Cancer*, 94(10), pp.2569–2576. https://doi.org/10.1002/cncr.10538.

Weng, Q., Jiang, J., Haji, F.M., Nondo, L.H. and Zhou, H. (2020). Women’s knowledge of and attitudes toward cervical cancer and cervical cancer screening in Zanzibar, Tanzania: a cross-sectional study. *BMC Cancer*, 20(1). https://doi.org/10.1186/s12885-020-6528-x.

Winer, R.L., Gonzales, A.A., Noonan, C.J., Cherne, S.L. and Buchwald, D.S. (2016). Assessing Acceptability of Self-Sampling Kits, Prevalence, and Risk Factors for Human Papillomavirus Infection in American Indian Women. *Journal of Community Health*, 41(5), pp.1049–1061. https://doi.org/10.1007/s10900-016-0189-3.

Wong, B.C-Y., Chan, A.O.O., Wong, K.W., Ching, C.K., Wong, W.M., Tam, S., Lai, K.C., Chan, C.K., Yuen, M.F. and Lam, S.K. (2005). A pilot study of participation in faecal occult blood testing and screening colonoscopy after health education in Hong Kong. *European Journal of Cancer Prevention*, 14(2), pp.181–184. https://doi.org/10.1097/00008469-200504000-00015.

Wong, E.L.-Y., Cheung, A.W.-L., Wong, A.Y.-K. and Chan, P.K.-S. (2020). Acceptability and Feasibility of HPV Self-Sampling as an Alternative Primary Cervical Cancer Screening in Under-Screened Population Groups: A Cross-Sectional Study. *International Journal of Environmental Research and Public Health*, 17(17), p.6245. https://doi.org/10.3390/ijerph17176245.

Wong, E.L.Y., Chan, P.K.S., Chor, J.S.Y., Cheung, A.W.L., Huang, F. and Wong, S.Y.S. (2016). Evaluation of the Impact of Human Papillomavirus DNA Self-sampling on the Uptake of Cervical Cancer Screening. *Cancer Nursing*, 39(1), pp.E1–E11. https://doi.org/10.1097/ncc.0000000000000241.

Wong, E.L.Y., Cheung, A.W.L., Huang, F. and Chor, J.S.Y. (2018). Can Human Papillomavirus DNA Self-sampling be an Acceptable and Reliable Option for Cervical Cancer Screening in Female Sex Workers? *Cancer Nursing*, 41(1), pp.45–52. <https://doi.org/10.1097/ncc.0000000000000462>.

Wu, E., Tiggelaar, S. M., Jiang, T., Zhao, H., Wu, R., Wu, R., & Xu, F. (2017). Cervical cancer prevention-related knowledge and attitudes among female undergraduate students from different ethnic groups within China, a survey-based study. *Women & Health*, *58*(6), 661–684. <https://doi.org/10.1080/03630242.2017.1333076>

Yacouti, A., Baddou, R., El Mettat, K., Guennouni, M., El Got, A., Aquil, A., Ennaji, M., Luc, A., Benider, A. and Mouallif, M. (2023). Moroccan Women’s Attitudes Regarding Pap Smear Test and Adherence to Cervical Cancer Screening. *Journal of cancer education*, 38(5), pp.1649–1655. https://doi.org/10.1007/s13187-023-02317-5.

Yang, C., Chang, T., Chou, H., Chao, A., Hsu, S., Shih, Y., Huang, H., Lin, C., Chen, M., Sun, L., Huang, K., Wu, K., Hsieh, W., Huang, Y., Chen, L., Lu, C., Lin, H. and Cheng, C. (2024). Evaluation of a novel vaginal cells self‐sampling device for human papillomavirus testing in cervical cancer screening: A clinical trial assessing reliability and acceptability. *Bioengineering & Translational Medicine*, 9(4). https://doi.org/10.1002/btm2.10653.

Zammit, C.M., Brooks, A., M L Brotherton, J. and Nightingale, C.E. (2024). What young women (aged 24–29 years) in Australia think about self-collection for cervical screening: a brief report. *Sexual Health*, [online] 21(4). https://doi.org/10.1071/sh24033.

Zelli, J., Hum, S., Lofters, A. and Dunn, S. (2022). Clinician acceptability of self-collected human papillomavirus swabs as a primary cervical cancer screening method. *Canadian Family Physician*, 68(2), pp.e31–e38. https://doi.org/10.46747/cfp.6802e31.

Zhu, X., MacLaughlin, K.L., Fan, C., Jacobson, D.J., Jenkins, G.D., Jacobson, R.M. and Finney Rutten, L.J. (2021). Awareness of HPV Testing and Acceptability of Self-sampling for Cervical Cancer Screening Among Women in Minnesota. *Journal of General Internal Medicine*. https://doi.org/10.1007/s11606-021-06854-x.

# List of review studies that references were pulled from:

1. Aimagambetova G, Atageldiyeva K, Marat A, Suleimenova A, Issa T, Raman S, et al. Comparison of diagnostic accuracy and acceptability of self-sampling devices for human Papillomavirus detection: A systematic review. Preventive medicine reports. 2024;38(101643766):102590.
2. Ali O, Gupta S, Brain K, Lifford KJ, Paranjothy S, Dolwani S. Acceptability of alternative technologies compared with faecal immunochemical test and/or colonoscopy in colorectal cancer screening: A systematic review. Journal of Medical Screening. 2023;30(1):14-27.
1. 5.           Braz NSDF, Lorenzi NPC, Sorpreso ICE, Aguiar LMd, Baracat EC, Soares-Junior JM. The acceptability of vaginal smear self-collection for screening for cervical cancer: a systematic review. Clinics. 2017;72:183-187.
2. 6.           Cairns JM, Greenley S, Bamidele O, Weller D. A scoping review of risk-stratified bowel screening: current evidence, future directions. Cancer Causes & Control. 2022;33(5):653-685.
3. 7.           Chongsuwat T, Wang C, Sohn Y, Klump K. Digital cervicography for cervical cancer screening in low-resource settings: A scoping review. Gynecologic Oncology Reports. 2023;45:101130.
4. 8.           Cora-Cruz MS, Martinez O, Perez S, Fang CY. Evaluating human papillomavirus (HPV) self-sampling among Latinas in the United States: A systematic review. Cancer medicine. 2024;13(16):e70098.
5. 9.           Dannhauser FC, Taylor LC, Tung JSL, Usher-Smith JA. The acceptability and clinical impact of using polygenic scores for risk-estimation of common cancers in primary care: a systematic review. Journal of community genetics. 2024;15(3):217-234.
6. 10.        Di Gennaro G, Licata F, Trovato A, Bianco A. Does self-sampling for human papilloma virus testing have the potential to increase cervical cancer screening? An updated meta-analysis of observational studies and randomized clinical trials. Frontiers in Public Health. 2022;Volume 10 - 2022.
7. 11.        Dunlop KLA, Singh N, Robbins HA, Zahed H, Johansson M, Rankin NM, et al. Implementation considerations for risk-tailored cancer screening in the population: A scoping review. Preventive Medicine: An International Journal Devoted to Practice and Theory. 2024;181:1-12.
8. 12.        Dyer CEF, Jin F, Hillman RJ, Nyitray AG, Roberts JM, Law C, et al. Self-collected versus clinician-collected anal swabs for anal cancer screening: A systematic review and meta-analysis. International journal of cancer. 2025;156(1):79-90.
9. 13.        Dzobo M, Dzinamarira T, Jaya Z, Kgarosi K, Mashamba-Thompson T. Experiences and perspectives regarding human papillomavirus self-sampling in sub-Saharan Africa: A systematic review of qualitative evidence. Heliyon. 2024;10(12):e32926.
10. 14.        Dzobo M, Dzinamarira T, Maluleke K, Jaya ZN, Kgarosi K, Mashamba-Thompson TP. Mapping evidence on the acceptability of human papillomavirus self-sampling for cervical cancer screening among women in sub-Saharan Africa: a scoping review. BMJ Open. 2023;13(4):e062090.
11. 15.        Gezimu W, Demeke A, Bekele F, Alemu SS, Fikadu L, Demsash AW, et al. Overview of female healthcare providers' outlooks on cervical cancer screening: a narrative review. Frontiers in oncology. 2025;15(101568867):1496513.
12. 16.        Guittet L, Quipourt V, Aparicio T, Carola E, Seitz J-F, Paillaud E, et al. Should we screen for colorectal cancer in people aged 75 and over? A systematic review - collaborative work of the French geriatric oncology society (SOFOG) and the French federation of digestive oncology (FFCD). BMC Cancer. 2023;23(1):17.
13. 18.        Johnson LG, Armstrong A, Joyce CM, Teitelman AM, Buttenheim AM. Implementation strategies to improve cervical cancer prevention in sub-Saharan Africa: a systematic review. Implementation Science. 2018;13(1):28.
14. 19.        Madzima TR, Vahabi M, Lofters A. Emerging role of HPV self-sampling in cervical cancer screening for hard-to-reach women: Focused literature review. Can Fam Physician. 2017;63(8):597-601.
15. 20.        Mantula F, Toefy Y, Sewram V. Barriers to cervical cancer screening in Africa: a systematic review. BMC public health. 2024;24(1):525.
16. 21.        Marshall S, Vahabi M, Lofters A. Acceptability, Feasibility and Uptake of HPV Self-Sampling Among Immigrant Minority Women: a Focused Literature Review. Journal of Immigrant and Minority Health. 2019;21(6):1380-1393.
17. 24.        Morgan K, Azzani M, Khaing SL, Wong Y-L, Su TT. Acceptability of Women Self-Sampling versus Clinician-Collected Samples for HPV DNA Testing: A Systematic Review. Journal of Lower Genital Tract Disease. 2019;23(3):193-199.
18. 25.        Narvaez L, Viviano M, Dickson C, Jeannot E. The acceptability of HPV vaginal self-sampling for cervical cancer screening in Latin America: A systematic review. Public Health in Practice. 2023;6:100417.
19. 26.        Osei EA. Innovations in healthcare delivery: Human papilloma virus self sampling diagnostics and participatory innovations for CCS. Cancer Medicine. 2023;12(14):15544-15551.
20. 27.        Otieno JA, Were L, Nyanchoka M, Olwanda E, Mulaku M, Sem X, et al. Human papillomavirus self-sampling versus provider-sampling in low- and middle-income countries: a scoping review of accuracy, acceptability, cost, uptake, and equity. Frontiers in public health. 2024;12(101616579):1439164.
21. 28.        Pahwa M, Cernat A, Abelson J, Demers PA, Schwartz L, Shen K, et al. Public perspectives on the benefits and harms of lung cancer screening: A systematic review and mixed-method integrative synthesis. Journal of health services research & policy. 2024:13558196241288984.
22. 29.        Paudyal P, Flohr FD, Llewellyn CD. A systematic review of patient acceptance of screening for oral cancer outside of dental care settings. Oral Oncology. 2014;50(10):956-962.
23. 30.        Pittalis C, Panteli E, Schouten E, Magongwa I, Gajewski J. Breast and cervical cancer screening services in Malawi: a systematic review. BMC Cancer. 2020;20(1):1101.
24. 31.        Rainey L, van der Waal D, Wengström Y, Jervaeus A, Broeders MJM. Women's perceptions of the adoption of personalised risk-based breast cancer screening and primary prevention: a systematic review. Acta Oncol. 2018;57(10):1275-1283.
25. 32.        Sabeena S, Ravishankar N, Kalpana MM. Implementation strategies of cervical cancer screening in South Asia: A systematic review. International journal of gynaecology and obstetrics: the official organ of the International Federation of Gynaecology and Obstetrics. 2024;166(2):483-493.
26. 33.        Salisbury A, Ciardi J, Norman R, Smit AK, Cust AE, Low C, et al. Public Preferences for Genetic and Genomic Risk-Informed Chronic Disease Screening and Early Detection: A Systematic Review of Discrete Choice Experiments. Applied health economics and health policy. 2024(101150314).
27. 34.        Srinath A, van Merode F, Rao SV, Pavlova M. Barriers to cervical cancer and breast cancer screening uptake in low- and middle-income countries: a systematic review. Health Policy and Planning. 2022;38(4):509-527.
28. 35.        Stewart DE, Gagliardi A, Johnston M, Howlett R, Barata P, Lewis N, et al. Self-collected samples for testing of oncogenic human papillomavirus: a systematic review. J Obstet Gynaecol Can. 2007;29(10):817-828.
29. 36.        Tan NQP, Nargund RS, Douglas EE, Lopez-Olivo MA, Resong PJ, Ishizawa S, et al. Acceptability and perceptions of personalised risk-based cancer screening among health-care professionals and the general public: a systematic review and meta-analysis. The Lancet Public health. 2025;10(2):e85-e96.
30. 37.        Tatar O, Wade K, McBride E, Thompson E, Head KJ, Perez S, et al. Are Health Care Professionals Prepared to Implement Human Papillomavirus Testing? A Review of Psychosocial Determinants of Human Papillomavirus Test Acceptability in Primary Cervical Cancer Screening. Journal of Women's Health. 2020;29(3):390-405.
31. 38.        Taylor LC, Law K, Hutchinson A, Dennison RA, Usher-Smith JA. Acceptability of risk stratification within population-based cancer screening from the perspective of healthcare professionals: A mixed methods systematic review and recommendations to support implementation. PLoS One. 2023;18(2):e0279201.
32. 39.        Wade R, Nevitt S, Liu Y, Harden M, Khouja C, Raine G, et al. Multi-cancer early detection tests for general population screening: a systematic literature review. Health technology assessment (Winchester, England). 2025;29(2):1-105.
33. 40.        Wang X, Li Y-J, Zhou X-Y, Wu Y-J, Huang H-Y, Zhang L, et al. Evaluation indicators for access to cancer screening services: a scoping review. Public health. 2024;235:194-201.
34. 41.        Wong JPH, Vahabi M, Miholjcic J, Tan V, Owino M, Li ATW, et al. Knowledge of HPV/cervical cancer and acceptability of HPV self-sampling among women living with HIV: A scoping review. Curr Oncol. 2018;25(1):e73-e82.
